# Novel Use of Flu Surveillance Data: Evaluating Potential of Sentinel Populations for Early Detection of Influenza Outbreaks

**DOI:** 10.1371/journal.pone.0158330

**Published:** 2016-07-08

**Authors:** Ashlynn R. Daughton, Nileena Velappan, Esteban Abeyta, Reid Priedhorsky, Alina Deshpande

**Affiliations:** 1 Analytics, Intelligence and Technology Division, Los Alamos National Laboratory, Los Alamos, NM, United States of America; 2 Bioscience Division, Los Alamos National Laboratory, Los Alamos, NM, United States of America; 3 High Performance Computing Division, Los Alamos National Laboratory, Los Alamos, NM, United States of America; Monash University, Australia, AUSTRALIA

## Abstract

Influenza causes significant morbidity and mortality each year, with 2–8% of weekly outpatient visits around the United States for influenza-like-illness (ILI) during the peak of the season. Effective use of existing flu surveillance data allows officials to understand and predict current flu outbreaks and can contribute to reductions in influenza morbidity and mortality. Previous work used the 2009–2010 influenza season to investigate the possibility of using existing military and civilian surveillance systems to improve early detection of flu outbreaks. Results suggested that civilian surveillance could help predict outbreak trajectory in local military installations. To further test that hypothesis, we compare pairs of civilian and military outbreaks in seven locations between 2000 and 2013. We find no predictive relationship between outbreak peaks or time series of paired outbreaks. This larger study does not find evidence to support the hypothesis that civilian data can be used as sentinel surveillance for military installations. We additionally investigate the effect of modifying the ILI case definition between the standard Department of Defense definition, a more specific definition proposed in literature, and confirmed Influenza A. We find that case definition heavily impacts results. This study thus highlights the importance of careful selection of case definition, and appropriate consideration of case definition in the interpretation of results.

## Introduction

Each year, several hundred thousand Americans contract seasonal influenza [1]. Of these, roughly 130,000 cases are hospitalized, with elderly and very young persons disproportionately at risk of serious complications [2]. Because of the breadth and magnitude of these outbreaks, improved surveillance and control of influenza has wide reaching consequences.

Official CDC reports of flu in the United States lag behind the real-time outbreak by 1 to 2 weeks, and are based on a compilation of data from hundreds of laboratories, and thousands of healthcare providers in numerous cities across the country [3]. For new datastreams to improve real-time knowledge of flu outbreaks they have to provide actionable data a minimum of a week earlier than standard methods. Further, lags in data are due largely to processing and analysis time, not because of lags in acquiring data. Accordingly, more prompt use of existing data can potentially inform outbreak surveillance and control without additional public health infrastructure.

Similar to the CDC, the Armed Forces Health Surveillance Center (AFHSC) collects all reportable healthcare events for the United States military in the Defense Military Surveillance System (DMSS) database. The consistent manner with which data are reported results in a comparatively complete set of disease surveillance data, akin to official CDC reports.

Using these data sources, Peter Riley et al. analyzed similarities between military and civilian outbreaks of influenza-like-illness during the 2009 season, with the goal of understanding if one population could be used as sentinel surveillance for the other [4]. If true, this could provide a new, accurate method to improve influenza surveillance without additional surveillance streams.

This project aimed to reproduce previous conclusions on a broader dataset, and further understand the limitations of that data based on case definitions used. We specifically asked the following two research questions (RQs):

### RQ 1: Do these data support the hypothesis that civilian data can be used as sentinel surveillance?

For the 2009–2010 flu season Riley et al. found:

1. High similarity (i.e. good correlation) between time series of civilian and military outbreaks occurring in the same location.
2. That typical military outbreaks peaked roughly one week after civilian outbreaks.
3. Preliminary evidence that in a small subset of outbreaks, the trend is reversed and military outbreaks peak before xcivilian ones [4]

Riley et al. further suggest the “local civilian population is driving the timing of peak incidence in many military installations” [4]. We thus hypothesized that we would see similar trends when looking at more flu seasons than just the 2009–2010 season. To test this hypothesis, we examined military and civilian ILI and influenza outbreaks from 2000 to 2013 in 7 locations, domestically and globally, and quantified similarity and sentinel capacity. We looked at the relative peaks of paired outbreaks as well as general consistency in time series as metrics of the quality of any existing trends.

### RQ 2: How are results from RQ1 affected when testing the effect of varying military ILI case definitions?

The level of detail present in the military data allowed us to investigate the time series resulting from two different ILI definitions in addition to confirmed influenza A cases. We examined the standard, broad Department of Defense (DoD) definition for influenza-like-illness and, based on Eick-Cost et al., investigated another definition that was found to describe flu more specifically, and with higher positive predictive value [5]. We compared the resulting ILI outbreak time series to each other, and to the time series of confirmed influenza A in the military in order to understand how case definition affected outbreaks. These analyses were done using a Pearson correlation.

## Materials and Methods

### Datasets

Military ILI and influenza data were acquired through the Armed Forces Health Surveillance System’s Defense Military Surveillance System (DMSS) database. This database houses information on hospitalizations, ambulatory visits and reportable medical events including relevant ICD-9 codes and related laboratory records [6]. This database was used to identify datasets 1 and 2.

We obtained IRB (institutional review board) approval for use of this data. Both LANL and AFHSB’s IRBs reviewed the protocols of analysis and approved them. Additionally, AFHSC de-identified data prior to use and analysis by LANL, and data was aggregated before publication.

#### Dataset 1: Broad ILI

Influenza-like-illness data from January 2000 until December 2013 was obtained from the Armed Forces Health Surveillance Center (AFHSC). This dataset included any records that met the United States’ Department of Defense (DoD) influenza-like-illness (ILI) definition. This corresponds to ICD-9 codes 079.99, 382.9, 460, 461.9, 465.8, 465.9, 466.0, 486, 487.0, 487.1, 487.8, 488.xx, 490, 780.6, 786.2 (see Table 1 ‘Broad’ for additional details).

**Table 1:** Military ILI definition ICD-9 codes and associated case definitions.

ICD-9 Code	Description	Broad	Narrow
079.99	Unspecified viral infection	X	
382.9	Unspecified otitis media	X	
460	Acute nasopharyngitis [common cold]	X	
461.9	Acute sinusitis, unspecified	X	
465.8	Acute upper respiratory infections of other multiple sites	X	
465.9	Acute upper respiratory infections of unspecified site	X	
466.0	Acute bronchitis	X	
486	Pneumonia, organism unspecified	X	
487.0	Influenza with pneumonia	X	X
487.1	Influenza with other respiratory manifestations	X	X
487.8	Influenza with other manifestations	X	
488.xx	Influenza due to certain identified influenza viruses	X	
488.82	Influenza due to identified novel influenza A virus with other respiratory manifestations [8]		X
490	Bronchitis, not specified as acute or chronic	X	
780.6	Fever	X	
786.2	Cough	X	

#### Dataset 2: Narrow ILI

Eick-Cost et al. proposed a definition of ILI that resulted in a higher specificity and positive predictive value than the original DoD definition, but lower sensitivity [5]. The ICD-9 codes used in the narrower definition were 488.82, 487.0, 487.1 (see Table 1 ‘Narrow’ for additional details). This dataset included records from Dataset 1 that met the more narrow definition.

#### Dataset 3: Lab-confirmed Influenza A

Dataset 3 included a line list of records of influenza tests and their results, from January 2006 until September 2013. Additional information like gender, military status (via an individual’s ‘patcat code’), and age were also included. Test data was formatted in multiple ways. Some columns included free text information while other columns attempted to aggregate free text data into generalized categories (e.g. ‘A positive’). We specifically used a combination of free text and aggregated fields to identify positive influenza A cases, codes to identify active duty military members, and sample collection dates to identify the influenza A time series. Laboratory data was also provided by AFHSC.

Raw line list data for military ILI and laboratory-confirmed Influenza A were provided by AFHSC. Aggregated reports of that data are available through the AFHSC website [7]. Aggregated seasonal data used for these analyses are provided in S1 Appendix.

#### Dataset 4: MMWR Weeks

Data from AFHSC was categorized into weeks based on a SAS algorithm that did not correspond to the CDC’s epidemiological week used for the Morbidity and Mortality Weekly report (MMWR). This dataset was used to translate SAS weeks into MMWR weeks for appropriate comparison to civilian data, and was provided by AFHSC.

#### Civilian Datasets

Civilian influenza-like-illness data was obtained from peer reviewed literature and official public health reports including CDC’s MMWR and public health websites. A comprehensive list of sources used for raw data are available in Table 2. Civilian datasets are named based on the ISO 3166-1 alpha-2 country codes of the country [9].

**Table 2:** Civilian datasets.

Name	Location	Citation
gu.civilian	Guam	[10]
jp.civilian	Japan	[11]
kr.civilian	South Korea	[12]
us.ca.civilian	California	[13]
us.md.civilian	Maryland	[14]
us.nc.civilian	North Carolina	[15]
us.tx.civilian	Texas	[16]

The seasonal civilian data used for these analyses are also available in S1 Appendix.

### Data Processing and Analysis

**Fig 1:**
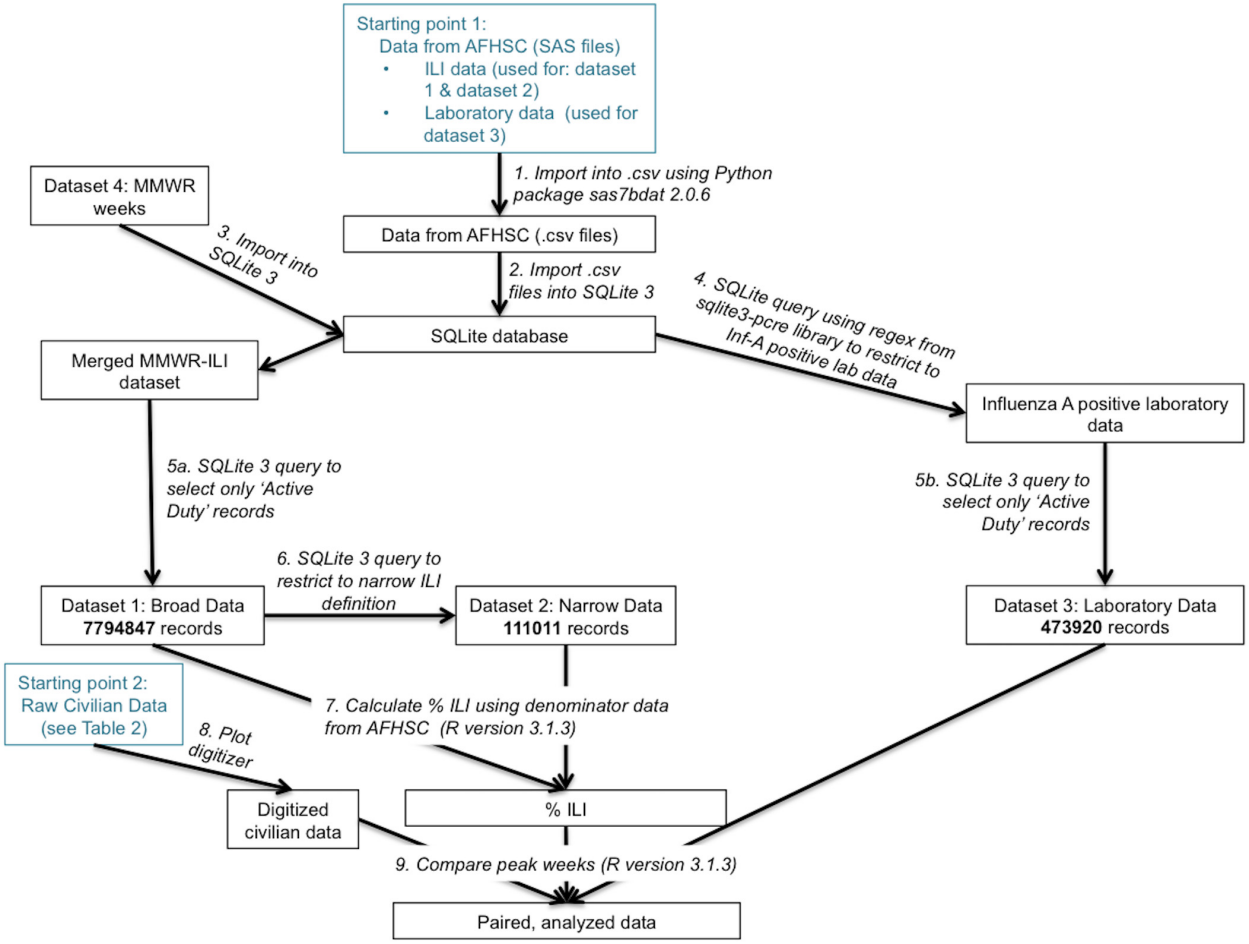
Data processing and related tools/ software. This figure illustrates how each dataset was transformed from the raw data to the processed data used in analyses. Starting points for raw data are shown in blue. Each box indicates a dataset at some stage of processing (e.g. a ‘noun’). Each arrow corresponds to the tools and actions used to transform one dataset into another (e.g. the ‘verb’ acting on the dataset ‘noun’) and is numbered to correspond with the text narrative. Dataset names correspond to those used above. Ultimately, all data was converted into time series of %ILI or confirmed cases. Then outbreaks from the same season were paired based on geographic proximity and analyzed.

Because data was accumulated from various sources and required different levels of processing, a visual representation of the procedure is given in Fig 1.

Data obtained from AFHSC was housed in SAS files. These files were converted to.csv files using the sas7bdat 2.0.6 Python package [17] (step 1) and then imported into a SQLite database (step 2). The ILI data were then merged with a dataset that converted SAS weeks to MMWR weeks for standardization (step 3). Records from this dataset that were active duty military members (step 5a) comprised the roughly 7.8 million records in Dataset 1. Dataset 2 was further restricted to include only 3 ICD-9 codes (see Table 1, step 6). This resulted in just over 1 million records in the narrow dataset. Using data from AFHSC regarding how many individuals visited each clinic site each week, we were able to convert these data to %ILI (step 7).

To process laboratory data, regular expressions were used to extract confirmed influenza A cases from the raw AFHSC laboratory data (step 4). We examined if free text fields reported that 1) a test for influenza A was conducted and 2) it was determined to be positive. A handful of examples illustrating ‘influenza A positive’ as well as examples for ‘not influenza A positive’ are included in Table 3. If a ‘positive’ word was qualified with another word like ‘presumptive’ or ‘weak’ we excluded the record from further analysis. These analyses were performed in SQLite using the sqlite3-pcre package which enables the use of Perl regular expressions [18]. Independently, AFHSC performed a similar analysis and grouped their data into ‘A Positive’, ‘B positive’ and ‘Both A and B positive’ records. Our final Influenza A positive dataset included records that either we or AFHSC defined as influenza A positive. This constituted just under 500 thousand records. The regular expressions used to derive our results, as well as a complete list of synonyms used for ‘Influenza A’, ‘Positive’ and ‘Negative’ are given in S2 Table. Any records that were not active duty military members were excluded (step 5b). The resulting dataset comprised Dataset 3.

**Table 3:** Examples of free text test result responses that were scored ‘Positive’ and ‘Not Positive’.

Positive	Not Positive
01 MAR 2011: POSITIVE FOR INFLUENZA A BY DFA.	“PRESUMPTIVE NEGATIVE” FOR INFLUENZA A/B
POSIITVE FOR INFLUENZA A AG [sic]	POSITIVE, PERFORMANCE CONTROLS VALID [No mention of Influenza A]
ATHE PATIENT HAS INFLUENZA A [sic]	TEST NEGATIVE FOR INFLUENZA A.
TYPE A POSITIVE	9/10 PRESUMPTIVE POSITIVE FOR INFLUENZA A. SENT TO REFERENCE FOR [sic; ‘presumptive’ and ‘probable’ positives were excluded]
13 MAY 09: SWINE INFLUENZA A (H1N1) DETECTED BY DOH RT-PCR.	NEG
H1	NOTDETECTED [sic]

Because of the known poor sensitivity of rapid influenza tests, and the resulting high rate of false-negatives, [19] we discarded non-positive results entirely rather than attempt to find a % positive influenza metric.

Raw civilian data were acquired through the data sources listed in Table 2. Any data in non-tabular format were converted to time series through the use of plot digitizer (step 8) [20]. Lastly, civilian outbreaks were paired with outbreaks from Datasets 1–3 from the same season, in the same geographic location. Military outbreak locations were identified using DMIS IDs, codes that correspond to each location in the military [21]. Each ‘season’ was defined from the 26th week of the first year until the 25th week of the following year. Using R, we identified the peak week as the week with the most cases, or highest percent of ILI and compared peaks among the various pairs (step 9). Peak difference is defined as the difference in number of weeks between civilian and military peaks or:

Peak Difference (# weeks) = Civilian Peak (week) − Military Peak (week)

A positive peak difference indicates that military data peaks first, while a negative peak difference indicates that civilian data peaks first. In addition to peak analysis, we explored general consistency between time series via correlation coefficients. Pearson correlations were used here.

Peak comparison was selected because (1) it was used by Riley et. al [4] and (2) measuring relative peaks is common in epidemiology studies (e.g. [22] [23]).

Our study included 91 broad military outbreaks, 88 narrow military outbreaks, 33 confirmed military influenza A outbreaks and 58 civilian ILI outbreaks between 2000 and 2013 in seven different locations (see Table 4), thus generating 58 military-civilian ILI outbreak pairs for both the ‘broad’ and ‘narrow’ groups (see Table 5). We excluded ILI seasons with less than or equal to 4 data points per season, as fewer data points resulted in epidemic curves that were too sparse to generate conclusions. This occurred in 3 ‘narrow’ military ILI outbreaks, all in Guam. We had no corresponding civilian data for these 3 seasons, so the number of civilian—military pairs were not affected. Additionally, we originally extracted confirmed influenza A data for 40 outbreaks from the U.S. military. Seven of the original outbreaks had only 1 case at the “peak” and were thus excluded, leaving 33 outbreaks in the analysis. To assess impacts of case definition (RQ2), we compared 87 pairs of broad and narrow military outbreaks occurring in the same season and location.

**Table 4:** Outbreak years and number outbreaks included per location Outbreak years (Number of outbreaks).

Location	Military Broad	Military Narrow	Military Confirmed	Civilian
California	2000–2013 (13)	2000–2013 (13)	2007–2013 (6)	2006–2013 (13)
Maryland	2000–2013 (13)	2000–2013 (13)	2007–2011, 2012–2013 (5)	2004–2010, 2012–2013 (7)
North Carolina	2000–2013 (13)	2000–2013 (13)	2007–2011, 2012–2013 (5)	2003–2013 (10)
Texas	2000–2013 (13)	2000–2013 (13)	2006–2013 (7)	2006–2013 (7)
Guam	2000–2013 (13)	2000–2013 (10)	2008–2010 (2)	2010–2013 (3)
Japan	2000–2013 (13)	2000–2013 (13)	2008–2010 (2)	2005–2013 (9)
South Korea	2000–2013 (13)	2000–2013 (13)	2007–2013 (6)	2000–2004, 2009–2013 (5)

**Table 5:** Number outbreaks in each set of pairs.

Pair	Outbreaks per pair
Civilian ILI/ Military Broad	58
Civilian ILI/ Military Narrow	58
Civilian ILI/ Military Confirmed	29
Military Broad/ Military Narrow	87

## Results

We compared ILI definitions at each location to verify that our paired outbreak analysis was valid (see Table 6). All definitions of influenza-like-illness between countries were similar. However, differences exist within definitions of epidemiological weeks. Locations within the United States and South Korea report data in weeks that end on Saturday while Japan and Guam reported data in weeks ending on Sunday. Further, both the United States and Japan occasionally have a 53rd week, however the rules used to determine these differ between the two countries, such that the United States included a 53rd week in 2003 and 2008, while Japan included a 53rd week in 2009. Because of this January 3, 2009 is included in different weeks based on location:

- United States: January 3, 2009 ended and was included the 53rd week of 2008.
- Japan and Guam: January 3, 2009 was included in the 1st week of 2009, which ended on January 4, 2009 [29].
- South Korea: January 3, 2009 ended the 1st week of 2009.

**Table 6:** Comparison of case definition, units and epidemiological week in each of the 7 locations included in analysis.

Location	Definition (quoted)	Unit	Week
California	“any illness with fever (≥100°F or 37.8°C) and cough and/or sore throat (in the absence of a known cause other than influenza).” [24]	% outpatient visits for ILI	Sunday—Saturday
Maryland	“ILI is defined as fever (temperature of 100°F [37.8°C] or greater) and a cough and/or a sore throat without a KNOWN cause other than influenza” [25]	% outpatient visits for ILI	Sunday—Saturday
North Carolina	“ILI case definition is fever (100 degrees F or higher, oral or equivalent) and cough or sore throat.” [26]	% outpatient visits for ILI	Sunday—Saturday
Texas	“Influenza-like Illness (ILI) Case Definition: Fever (≥100°F [37.8°C], oral or equivalent), Cough and/or sore throat, Without a known cause other than influenza” [27]	% outpatient visits for ILI	Sunday—Saturday
Guam	“Sudden onset of fever, a with cough and/or sore throat” [28]	raw case count	Saturday—Sunday
Japan	“ILI was defined as sudden onset of fever (38.0°C) with cough or sore throat in the absence of other diagnoses.” [29]	ILI cases per sentinel per week	Saturday—Sunday
South Korea	“presence of (1) body temperature ≥ 38°C and (2) cough or rhinorrhea” [30]	ILI cases per 1000 visits at sentinel locations	Sunday—Saturday

Even with these variances the actual differences in data groupings vary by, at most, a one week difference. The following is an example from our data to illustrate the potential resulting bias:

- Broad military outbreak in Japan (uses U.S. epidemiological week) peak: 2005 week 16
- Civilian peak in Japan (uses Japan epidemiological week) peak: 2005 week 10
- Peak difference = civilian peak—military peak = -6 weeks (i.e. the military outbreak peaked 6 weeks before its civilian pair)

However, because weeks are slightly different, if the difference of 1 day would have changed the peak of either the U.S. military or the Japanese civilian population, the peak difference could be off by a week. It is intuitively unlikely that shifting either dataset one day would change the peak week. However, data should be viewed with the possibility in mind. It should also be noted that the majority of peak differences were greater than the potential one-week discrepancy. Thus the authors are confident that the conclusions drawn from these comparisons are valid. It should also be noted that all outbreak pairs occurred in the same location, and thus the same time zone. Therefore no artifacts were introduced because of differences in time zones.

**Fig 2:**
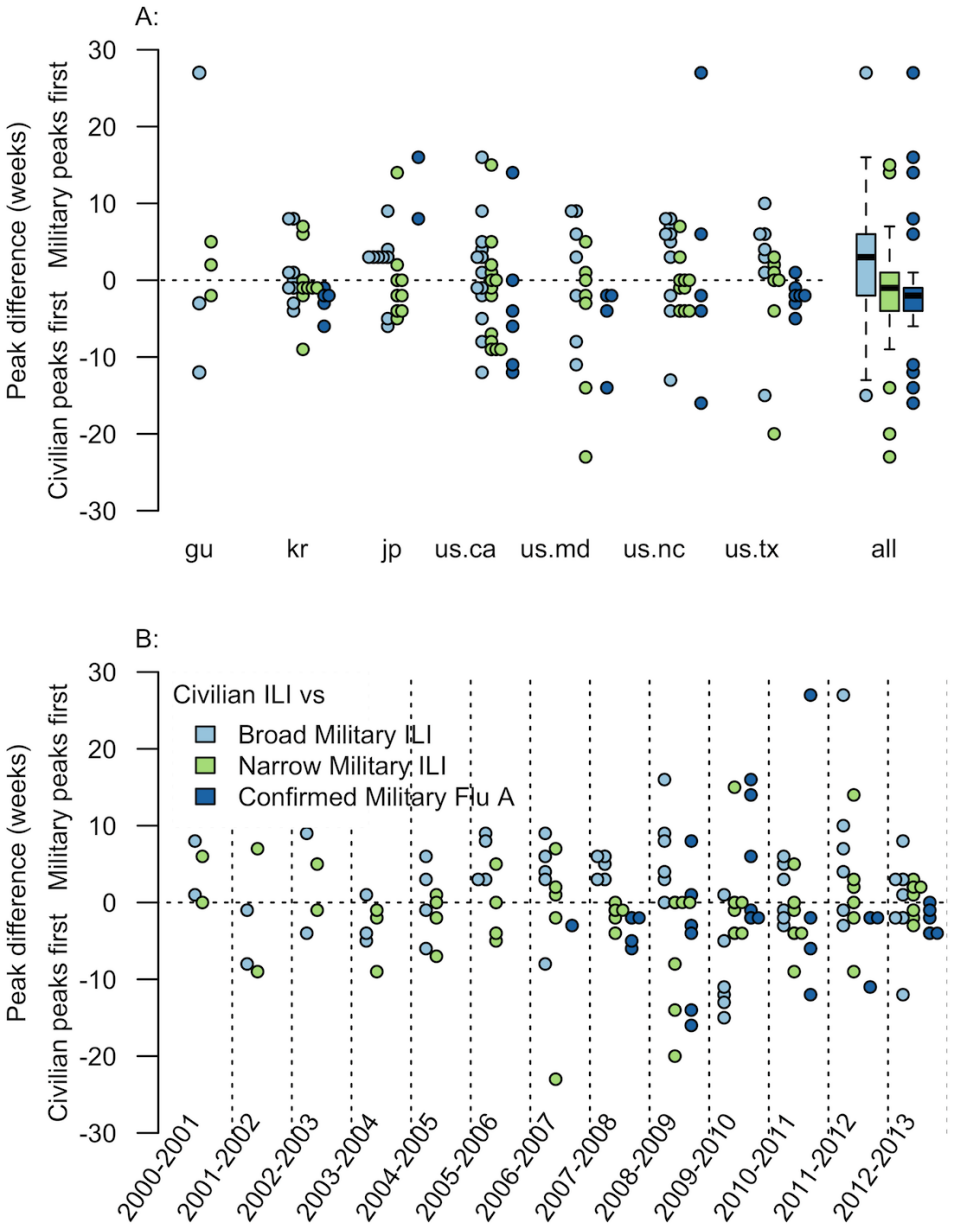
Peak Comparison by Location and Year. Civilian data was matched, based on year and location, to three military outbreak datasets: (1) Military ILI using the broad definition (dataset 1), (2) Military ILI using the narrow definition (dataset 2) and (3) confirmed military influenza A (dataset 3). Fig A: The right side of this graph shows the respective time between each individual civilian-military pairs’ peak with respect to each location analyzed. Multiple datapoints are included horizontally to better view depth in the data. Boxplots on the left side show aggregated sets of pairs, where the heavy dark line represents the median value, the top and bottom of the box are the first and third quartiles, the ends of the dashed lines represent the upper and lower ‘whiskers’, and the colored dots are outliers [31]. Labels in A are from the ISO 3166-1 alpha-2 country codes of the location of interest (see Table 2). The boxplots show that civilian outbreaks tend to peak first, however the military definition they are paired against heavily influences how much. Broad military ILI compared to civilian ILI has the greatest difference in point estimates of the peaks (see heavy black line in boxplot), while confirmed military influenza A outbreaks have the most similar peaks to civilian ILI data. Data are highly variable, as evidenced by both the scatterplots and boxplots. Fig B: This graph shows the respective time between each individual civilian-military pairs’ peak with respect to each year analyzed. Laboratory military data was only available post-2006 (dark blue). As with location data, there is large spread in the data, and no clear trends exist.

We compared each civilian outbreak to all corresponding military outbreaks, as described in Table 5. It should be noted that, per the analysis presented in Table 6, civilian ILI compared to the broad military ILI is the most ‘apples-to-apples’ comparison. We compared the lag in peak between military and civilian outbreaks aggregated by year, as well as location in order to assess both temporal and geographic trends (see Fig 2).

When considering the aggregate, civilian ILI outbreaks peak an average of 1.6 weeks (95% CI: -0.4 weeks to 3.6 weeks), and a median of 3 weeks after broad military outbreaks. When civilian ILI outbreaks are compared to narrow military ILI outbreaks or confirmed military influenza A outbreaks the trend is actually reversed, with military outbreaks peaking a median of 1 to 2 weeks after civilian outbreaks. Regardless of aggregation by location (Fig 2 top panel, S1 Table) or year (Fig 2 bottom panel, S1 Table), when comparing civilian ILI outbreak to broad outbreaks, there is a wide spread in relative peaks. This holds true regardless of the military definition used for comparison. Interestingly, when aggregating by year, civilian—broad pairs tend to have ‘civilian first’ peaks, while civilian—narrow pairs tend to have ‘military first’ peak, and civilian—military confirmed pairs seem to be predominately clustered around 0. However, when aggregating by location, all pairs seems to be distributed across ‘military first’ and ‘civilian first’ peaks. This is also reflected in the aggregate box plots.

Interestingly, the period and set of pairs analyzed by Riley et al. [4] seem to show similar conclusions. Riley et al. looked at pairs that correspond to our ‘civilian—broad military ILI’ pairs (light blue scatterplots), for 2009–2010. Our data for that season show that military outbreaks do tend to peak before civilian outbreaks, but with larger variation than reported by Riley et al. (see Fig 2)

**Fig 3:**
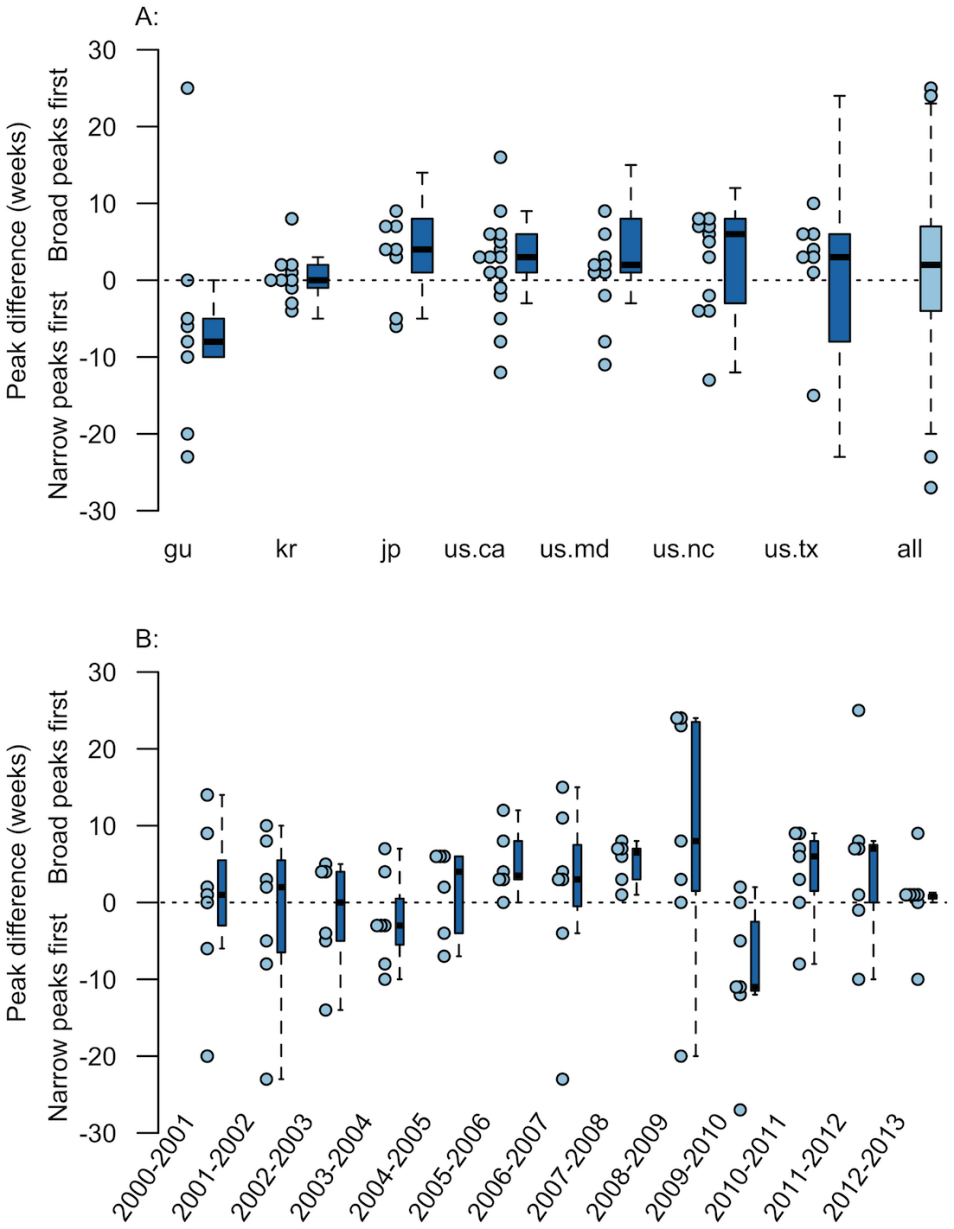
Narrow and Broad Military ILI Peak Comparison by Location and Year. Military broad (dataset 1) and military narrow (dataset 2) ILI outbreaks were paired based on year and location and the difference between their peaks was calculated. Panel A shows peak comparison by location using IISO 3166-1 alpha-2 country codes (see codes in Table 2) and the overall statistics (boxplot ‘all’). Panel B shows the same data aggregated by year. Raw data is shown in light blue points while corresponding boxplots (dark blue) are presented to show overall trends in data. Data are highly variable, indicating general dissimilarity when comparing outbreaks generated using the same set of data, but different case definitions. This highlights the need to carefully and select case definitions in epidemiological research.

We also compared peaks between the two military ILI definitions (Fig 3). Overall, the narrow military ILI definition generated outbreaks that peaked and average of 1.4 weeks after, and a median of 2 weeks after, the broad definition outbreaks (see S1 Table). The scatterplots aggregated by both year and location show, overall, a higher density of ‘narrow military definition peak first’ outbreaks, but there is still substantial variation over years (Fig 3 panel A) and in each location (Fig 3 panel B). These data indicate that the outbreaks produced by using different military ILI case definitions are visually different. If the outbreaks were identical or nearly so, we would see a relatively flat straight line around ‘0’.

In order to assess general trends in the relationship between these two time series we additionally compared the narrow and broad military ILI time series for all years, by each location (Table 7). Point estimates of the correlation between the two time series are all significant, but the relationships are modest at best (0.22–0.32). Taken together, this is evidence that these two definitions produce physically different outbreaks and emphasizes the importance of taking definitions into account when comparing outbreaks. Given that it is known that influenza-like-illness outbreaks commonly include a number of viruses other than the flu, including adenoviruses and rhinovirus [32–34], and that Eick-Cost et al. demonstrates low specificity within the DoD definition [6], it makes sense that the two definitions produce dissimilar outbreaks.

**Table 7:** Pearson Correlation: Military Narrow versus Broad ILI Definition.

Location	Correlation Estimate	P-value	95% CI
California	0.28	1.16E-13	0.21–0.35
Maryland	0.32	3.63E-12	0.24–0.40
North Carolina	0.31	5.42E-14	0.23–0.38
Texas	0.29	9.81E-14	0.22–0.36
Guam	0.26	8.42E-04	0.11–0.40
Japan	0.22	5.19E-07	0.30–0.13
South Korea	0.32	1.81E-11	0.23–0.40

## Discussion

### RQ 1: Do these data support the hypothesis that civilian data can be used as sentinel surveillance?

One original motivation of this study was to extend the Riley et al. hypothesis that either military or civilian data could be used as sentinel surveillance for flu outbreaks. Overall, we found that civilian outbreaks peak before military outbreaks in the 2009–2010 flu season, which corroborates the findings for the time period and pairs that Riley et al. analyzed. However, this trend did not extend to additional locations, years, or case definitions.

There are a few differences in between the Riley et al. methodology and our methodology that should be noted. Because we were not analyzing reproductive numbers during this study, we used the raw outbreak curves and did not fit SIR models to each outbreak. In addition, Riley et al. looked at local locations (e.g. specific DMISIDs), while our analyses occur at the state and country level. These differences might explain why we see a broader range of values than Riley et al. reported. However, when analyzing the 2009–2010 outbreak season our results are consistent with that of Riley et al. An additional discussion of the 2009–2010 outbreak season is given in the ‘Anomalous seasons’ section below.

### RQ 2: How are results from RQ1 affected when testing the effect of varying military ILI case definitions?

In addition to comparing civilian data to military data using the general Department of Defense ILI definition (dataset 1, “Broad” definition), we also wanted to assess trends when using a more narrow military ILI definition [5] and laboratory confirmed incidence of influenza A. The purpose here was to go from the broadest definition of ILI possible down to the most narrow influenza definition, while observing changes along the way. This is why we selected for influenza A specifically, instead of confirmed influenza more broadly. Interestingly, the two more narrow outbreak definitions created outbreaks that reversed the aggregate civilian—broad military ILI trend. Instead of observing the military outbreaks peaking first, the other two pairs result in the majority of civilian outbreaks peaking first. However, this observation is still caveated by the extremely high variation, both across locations, and through time (Fig 2).

The additional civilian—military pairs highlight the result that changes to case definition can heavily affect results and thus the conclusions reached. However, because they were from different populations, and because we were unable to modify civilian ILI definitions in a similar fashion, we chose to assess the impact of definition within the same population by comparing outbreaks generated through broad and narrow military ILI definitions (Fig 3). We found that narrow definition outbreaks peak a median of 2 weeks after broad outbreaks. Again, peak comparison varied extensively when stratifying by year and location. Correlations of the two time series were statistically significant, but small (Table 7). Overall, these results indicate that using the same set of data, but different case definitions produces dissimilar outbreaks. Given that previous work demonstrates the wide number of viruses responsible for ILI outbreaks, and the poor specificity of the DoD definition, the finding that the two sets of outbreaks appear to be different is expected. This data does indicate that, at minimum case definition needs to be carefully selected for epidemiological studies, and could also point towards a justification for modifying the U.S’s influenza-like-illness definition, to one that produces more specific results.

### Anomalous seasons

We also want to draw attention to the 2009–2010 outbreak season. Peak difference results from civilian vs broad military ILI and civilian vs confirmed military flu A pairs show that this season looks visually different than other seasons. This difference is expected due to the H1N1 pandemic, the resulting difference in health care usage, and the increase in public health publicity that year. Based on these differences, we postulate that outbreak conditions that are highly publicized are more likely to break trends. Thus, in order to assess outbreak trends generally, it is pertinent to use a dataset that combines heavily publicized outbreaks as well as more routine outbreaks. Epidemiological studies tend to focus on aberrant outbreaks (e.g. Haiti’s 2010 Cholera outbreak [35], West Africa’s ebola outbreak 2013–2015 [36], Mexico/ USA’s H1N1 outbreak in 2009 [37] etc). While these studies provide useful information on factors that lead to more extreme outbreaks, that data must also be situated with the ‘norm’ in order to effectively inform surveillance systems.

### Limitations

The lack of exact dates to use as comparisons between military and international civilian data should be noted when observing pairwise civilian and military results. Because all our analyses typically showed differences in peaks between civilian and military data of greater than the potential 1-week discrepancy, we are confident the differences we observe are real. However, we call attention to this point because it underlies the importance of understanding reporting specifics of epidemiological data when comparing across locations.

### Conclusions

Overall, this study serves to indicate that there are differences between military and civilian outbreaks, but it is difficult to find consistent trends in those differences. We did not find evidence for the hypothesis that civilian outbreaks might be useful as sentinel surveillance for military outbreaks. Further, and applicable to epidemiological work more broadly, case definitions used for comparison heavily affected the observed results. Moreover, the known fact that countries report data in inconsistent epidemiological weeks must be considered when comparing outbreaks in different locations. Overall, it is necessary that those in this field meticulously examine both case definition and reporting behaviors when comparing outbreaks, or performing studies to inform surveillance systems.

## Supporting Information

S1 Table Raw peak difference data: This data is available in the accompanying pdf ‘S1_Table’. The data used to produce Figs 2 and 3 are provided here. Peak differences equations are shown in the column headers.
(PDF)

S2 Table Regular expressions used: This data is available in the accompanying pdf ‘S2_Table’. Included are the seven regular expressions used to extract influenza A tests. Positive tests were determined by a flag included in the data. For reference, we include examples of both ‘positive’ and ‘influenza’ synonyms used to help identify regular expressions.
(PDF)

S1 Appendix Raw epidemiological curves: S1 Appendix information is available in the accompanying pdf ‘S1_Appendix’. This contains all raw data used in our peak analyses.
(PDF)
