# Supplementary material for: Novel Use of Flu Surveillance Data: Evaluating Potential of Sentinel Populations for Early Detection of Influenza Outbreaks
Source: PLoS One. 2016 Jul 8;11(7):e0158330. doi: 10.1371/journal.pone.0158330 (PMC4938434; doi:10.1371/journal.pone.0158330)
Supplement: S1 Table — The data used to produce Figs 2 and 3 are provided here. Peak differences equations are shown in the column headers. (PDF) [file pone.0158330.s001.pdf]

Location	Season	Narrow Mil - Broad Mil	Confirmed Mil - Broad Mil	Confirmed Mil - Narrow Mil	Civ - Mil broad	Civ - Confirmed Mil	Civ - Narrow Mil
Guam	2000-2001	-6					
Guam	2001-2002	-5					
Guam	2002-2003						
Guam	2003-2004	-10					
Guam	2004-2005						
Guam	2005-2006						
Guam	2006-2007	-23					
Guam	2007-2008						
Guam	2008-2009	-20	-4				
Guam	2009-2010	0	3	3			
Guam	2010-2011	-8			-3		5
Guam	2011-2012	25			27		2
Guam	2012-2013	-10			-12		-2
South Korea	2000-2001	2			8		6
South Korea	2001-2002	-8			-1		7
South Korea	2002-2003	-5			-4		-1
South Korea	2003-2004	7			1		-9
South Korea	2004-2005	-7					
South Korea	2005-2006	0					
South Korea	2006-2007	3					
South Korea	2007-2008	1	9	8			
South Korea	2008-2009	0	3	3	0	-3	0
South Korea	2009-2010	2	2	0	1	-1	-1
South Korea	2010-2011	0	5	5	-1	-6	-1
South Korea	2011-2012	-1	-1	0	-3	-2	-2
South Korea	2012-2013	9	6	-3	8	-2	-1
Japan	2000-2001	14					
Japan	2001-2002	10					
Japan	2002-2003	4					
Japan	2003-2004	4					
Japan	2004-2005	-4			-6		-2
Japan	2005-2006	8			3		-5
Japan	2006-2007	11			9		-2
Japan	2007-2008	7			3		-4
Japan	2008-2009	3	-5	-8	3	8	0
Japan	2009-2010	-5	-21	-16	-5	16	0
Japan	2010-2011	7			3		-4
Japan	2011-2012	-10			4		14
Japan	2012-2013	1			3		2
California	2000-2001	1			1		0
California	2001-2002	3			-8		-9
California	2002-2003	4			9		5

California	2003-2004	-3			-5		-2
California	2004-2005	6			-1		-7
California	2005-2006	3			3		0
California	2006-2007	3			4		1
California	2007-2008	6	11	5	5	-6	-1
California	2008-2009	24	20	-4	16	-4	-8
California	2009-2010	-27	-26	1	-12	14	15
California	2010-2011	9	10	1	-2	-12	-9
California	2011-2012	8	10	2	-1	-11	-9
California	2012-2013	1	3	2	3	0	2
Maryland	2000-2001	9					
Maryland	2001-2002	2					
Maryland	2002-2003	-14					
Maryland	2003-2004	-3					
Maryland	2004-2005	2			3		1
Maryland	2005-2006	4			9		5
Maryland	2006-2007	15			-8		-23
Maryland	2007-2008	8	8	0	6	-2	-2
Maryland	2008-2009	23	23	0	9	-14	-14
Maryland	2009-2010	-11	-9	2	-11	-2	0
Maryland	2010-2011	3	7	4			
Maryland	2011-2012	1					
Maryland	2012-2013	1	2	1	-2	-4	-3
North Carolina	2000-2001	0					
North Carolina	2001-2002	8					
North Carolina	2002-2003	-4					
North Carolina	2003-2004	-3			-4		-1
North Carolina	2004-2005	6			6		0
North Carolina	2005-2006	12			8		-4
North Carolina	2006-2007	-4			3		7
North Carolina	2007-2008	7	8	1	6	-2	-1
North Carolina	2008-2009	8	24	16	8	-16	0
North Carolina	2009-2010	-12	-22	-10	-13	6	-4
North Carolina	2010-2011	9	-22	-31	5	27	-4
North Carolina	2011-2012	7			7		0
North Carolina	2012-2013	1	2	1	-2	-4	3
Texas	2000-2001	-20					
Texas	2001-2002	-23					
Texas	2002-2003	5					
Texas	2003-2004	-8					
Texas	2004-2005	6					
Texas	2005-2006	3					
Texas	2006-2007	4	9	5 6	-3	2	
Texas	2007-2008	3	8	5 3	-5	0	
Texas	2008-2009	24	3	-21	4	1	-20
Texas	2009-2010	-11	13	-2	-15	-2	-4
Texas	2010-2011	6	8	2 6	-2	0	
Texas	2011-2012	7	12	5	10	-2	3
Texas	2012-2013	0	2	2	1	-1	1
