# Supplementary material for: Novel Use of Flu Surveillance Data: Evaluating Potential of Sentinel Populations for Early Detection of Influenza Outbreaks
Source: PLoS One. 2016 Jul 8;11(7):e0158330. doi: 10.1371/journal.pone.0158330 (PMC4938434; doi:10.1371/journal.pone.0158330)
Supplement: S2 Table — Included are the seven regular expressions used to extract influenza A tests. Positive tests were determined by a flag included in the data. For reference, we include examples of both ‘positive’ and ‘influenza’ synonyms used to help identify regular expressions. (PDF) [file pone.0158330.s002.pdf]

Supplementary Table S2 (Regular expressions used):

```
'.*FLU.*\WA\b'
'.*[\W\n]A ANTIGEN.*'
'H1'
'H3'
'.*TYPE.*A'
'A\W.*FLU.*'
'^A$'
```

**'Positive' synonyms**	**'Influenza' synonyms**
POSITIVE	FLU A
ISOLATED	INFLUENZA A
DETECTED	INFLUENZA VIRUS TYPE A
CONFIRMED	A ANTIGEN
PROBABLE	"A ANTIGEN"
POS	INFLUENZA H1
RECOVERED	PANDEMIC H1N1
WAS IDENTIFIED	INFLUENZA "A"
'ATHE PATIENT HAS INFLUENZA A' [sic]	A & B INFLUENZA
POSIITVE [sic]	A
	A/H1
	A/H1N1
	A:H1
	A/H3
	INFLUENZA VIRUS TYPES A
	INFLUENZA VIRUS GROUP A
	INFLUENZA VIRUS RNA TYPE A/H1
	INFLUENZA VIRUS RNA TYPE A/H3
	2009 H1N1
	INFLUENZA-A
	INFLUENZAE A
	INFLUENZAE TYPE A
	INFLUENZAE VIRUS TYPE A
	INFLUENZA TYPE "A"
	INFLUENZA-A
	INF-A
	TYPE A
	INFLU A
	INFLUENZE [sic]
	INFLUEZA A [sic]
	INLFLUENZA A [sic]
