# Supplementary material for: Novel Use of Flu Surveillance Data: Evaluating Potential of Sentinel Populations for Early Detection of Influenza Outbreaks
Source: PLoS One. 2016 Jul 8;11(7):e0158330. doi: 10.1371/journal.pone.0158330 (PMC4938434; doi:10.1371/journal.pone.0158330)
Supplement: S1 Appendix — This contains all raw data used in our peak analyses. (PDF) [file pone.0158330.s003.pdf]

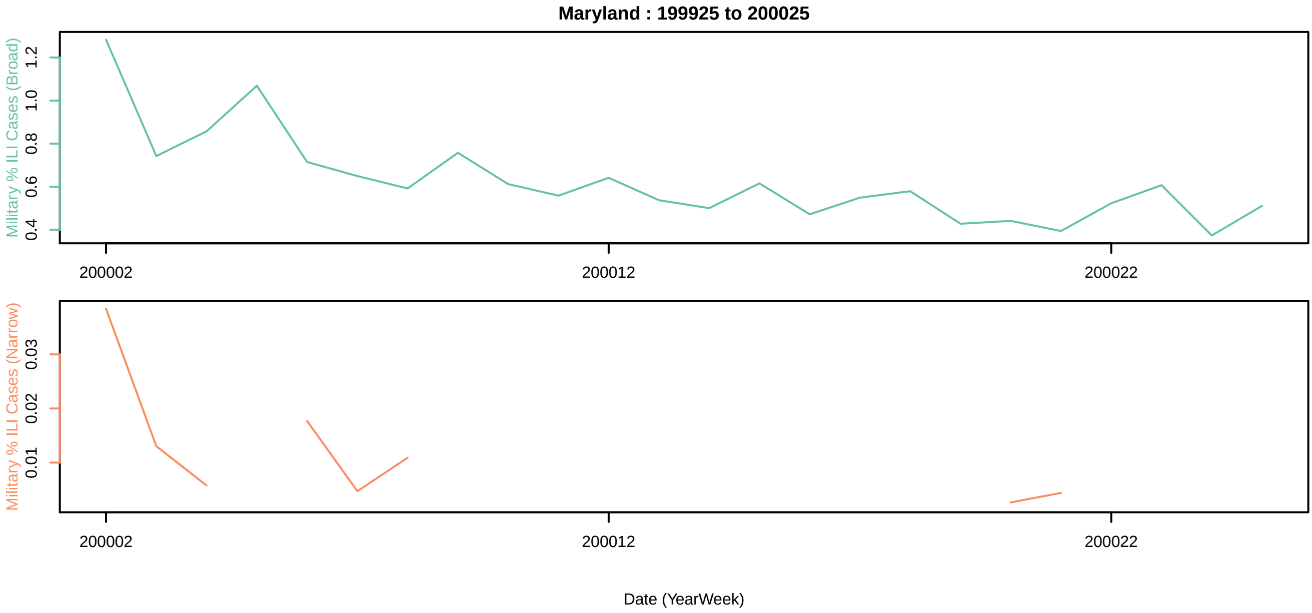
Maryland : 199925 to 200025
Military % ILI Cases (Broad)
0.4
0.6
0.8
1.0
1.2
200002
200012
200022
Military % ILI Cases (Narrow)
0.01
0.02
0.03
200002
200012
200022
Date (YearWeek)

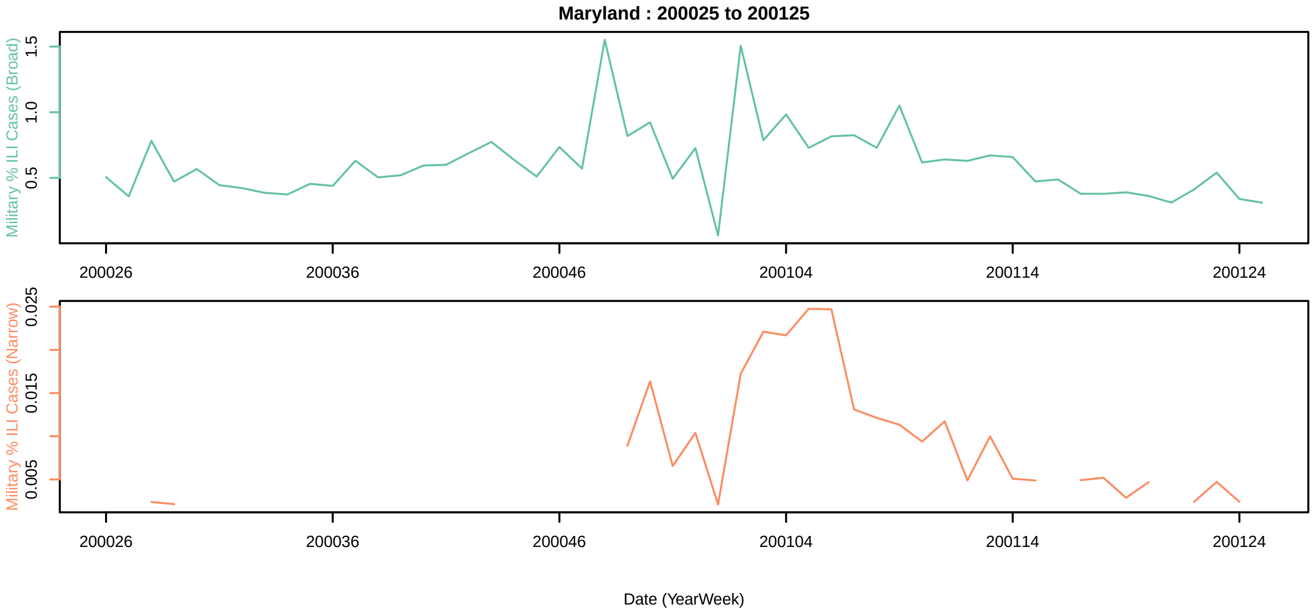
Maryland : 200025 to 200125
Military % ILI Cases (Broad)
0.5
1.0
1.5
Military % ILI Cases (Narrow)
0.005
0.015
0.025
200026
200036
200046
200104
200114
200124
Date (YearWeek)

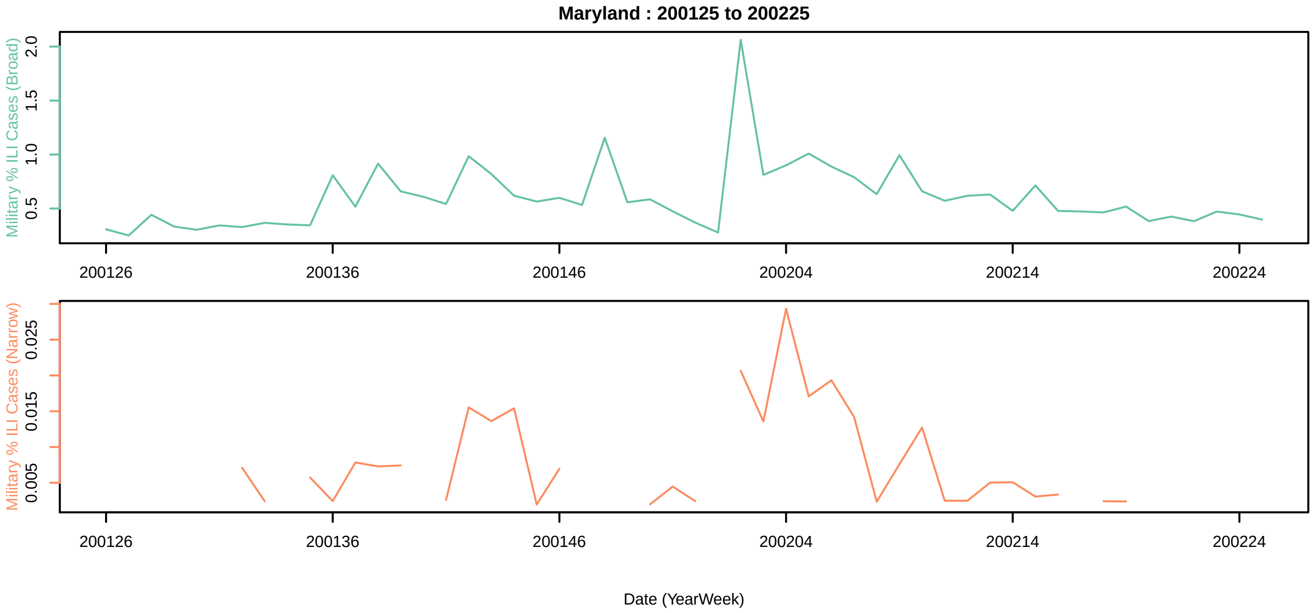

Maryland : 200125 to 200225
Military % ILI Cases (Broad)
0.5
1.0
1.5
2.0
200126
200136
200146
200204
200214
200224
Military % ILI Cases (Narrow)
0.005
0.015
0.025
200126
200136
200146
200204
200214
200224
Date (YearWeek)

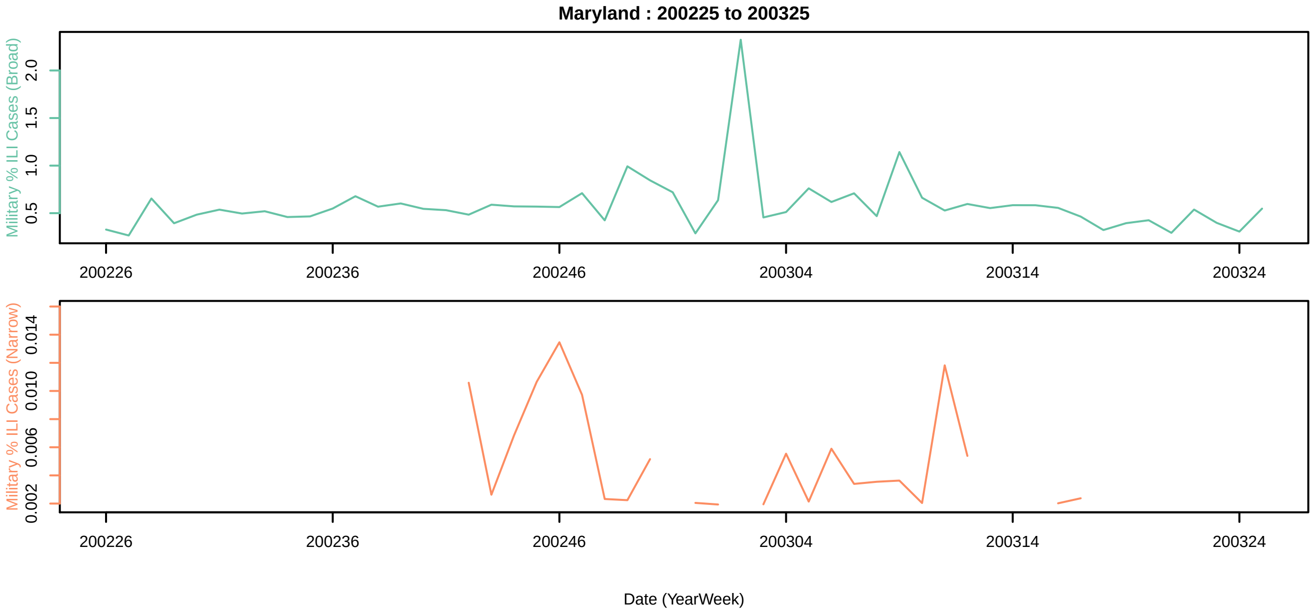

Maryland : 200225 to 200325
Military % ILI Cases (Broad)
0.5
1.0
1.5
2.0
200226
200236
200246
200304
200314
200324
Military % ILI Cases (Narrow)
0.002
0.006
0.010
0.014
200226
200236
200246
200304
200314
200324
Date (YearWeek)

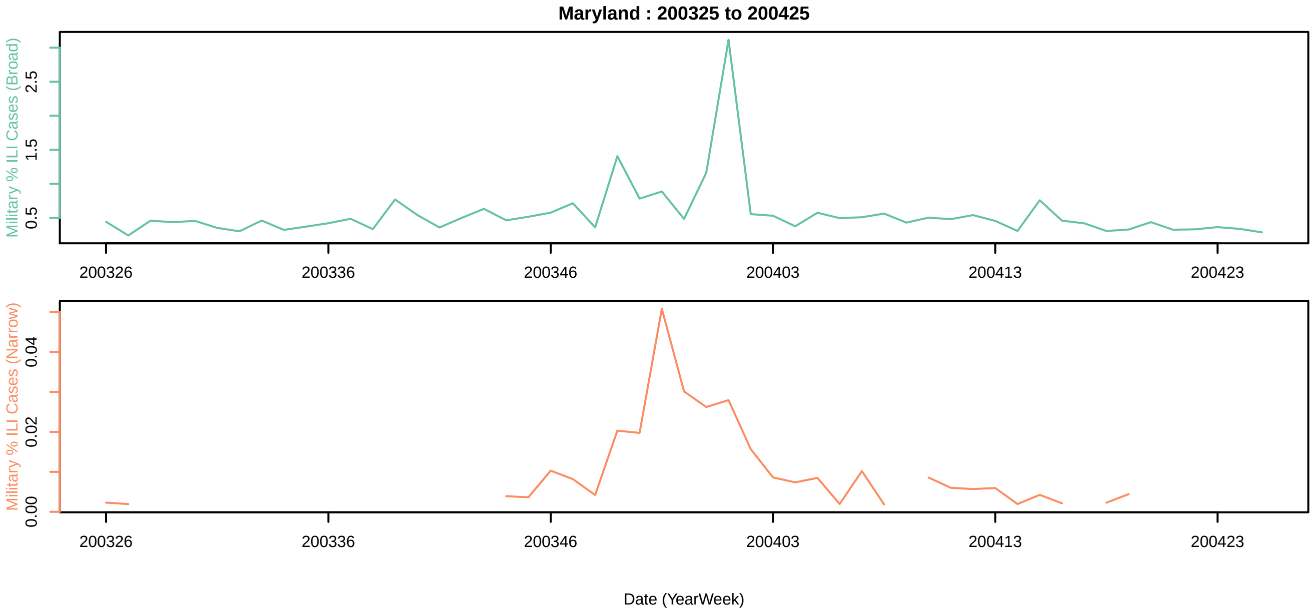

Maryland : 200325 to 200425
Military % ILI Cases (Broad)
0.5
1.5
2.5
200326
200336
200346
200403
200413
200423
Military % ILI Cases (Narrow)
0.00
0.02
0.04
200326
200336
200346
200403
200413
200423
Date (YearWeek)

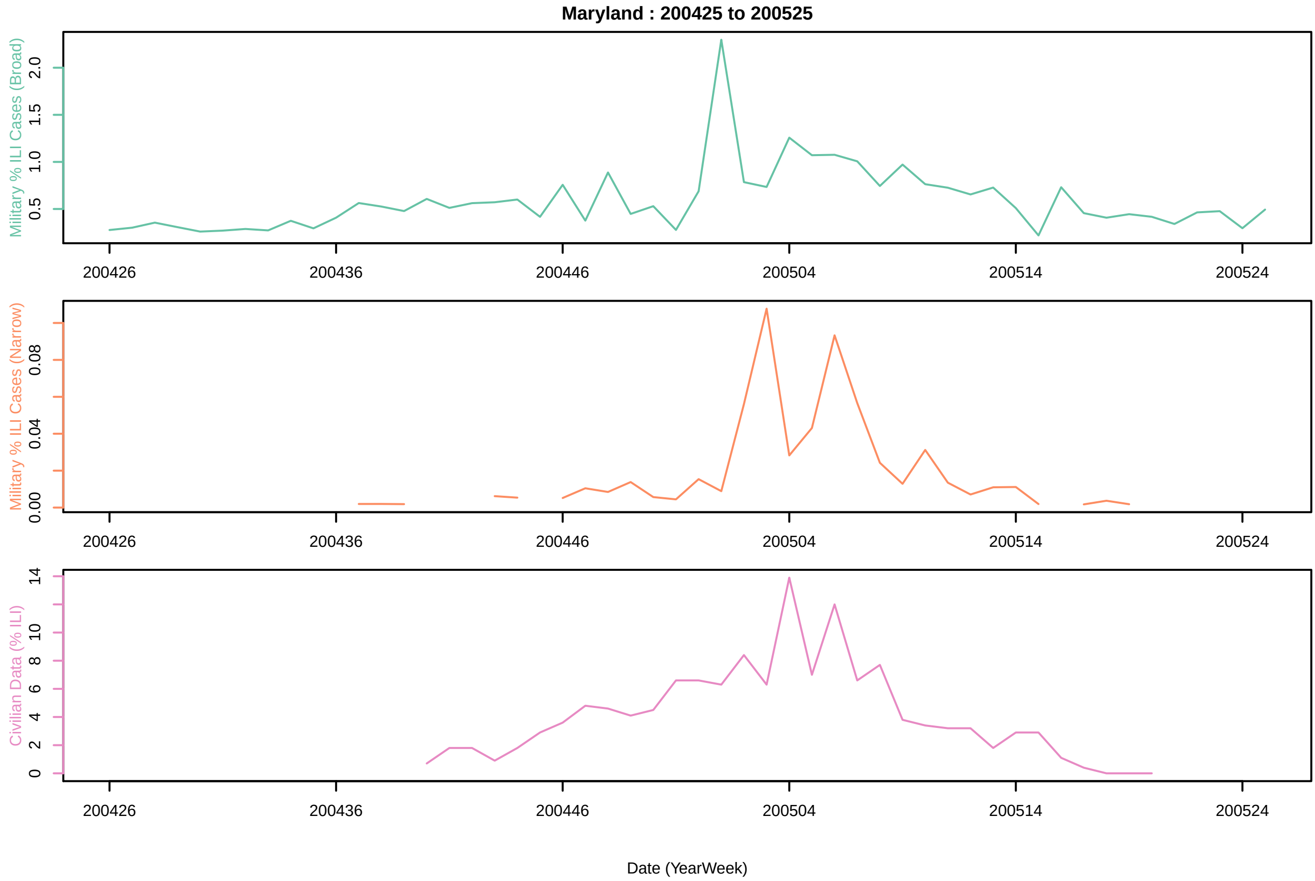

Maryland : 200425 to 200525
Military % ILI Cases (Broad)
0.5
1.0
1.5
2.0
Military % ILI Cases (Narrow)
0.00
0.04
0.08
Civilian Data (% ILI)
0
2
4
6
8
10
14
200426
200436
200446
200504
200514
200524
Date (YearWeek)

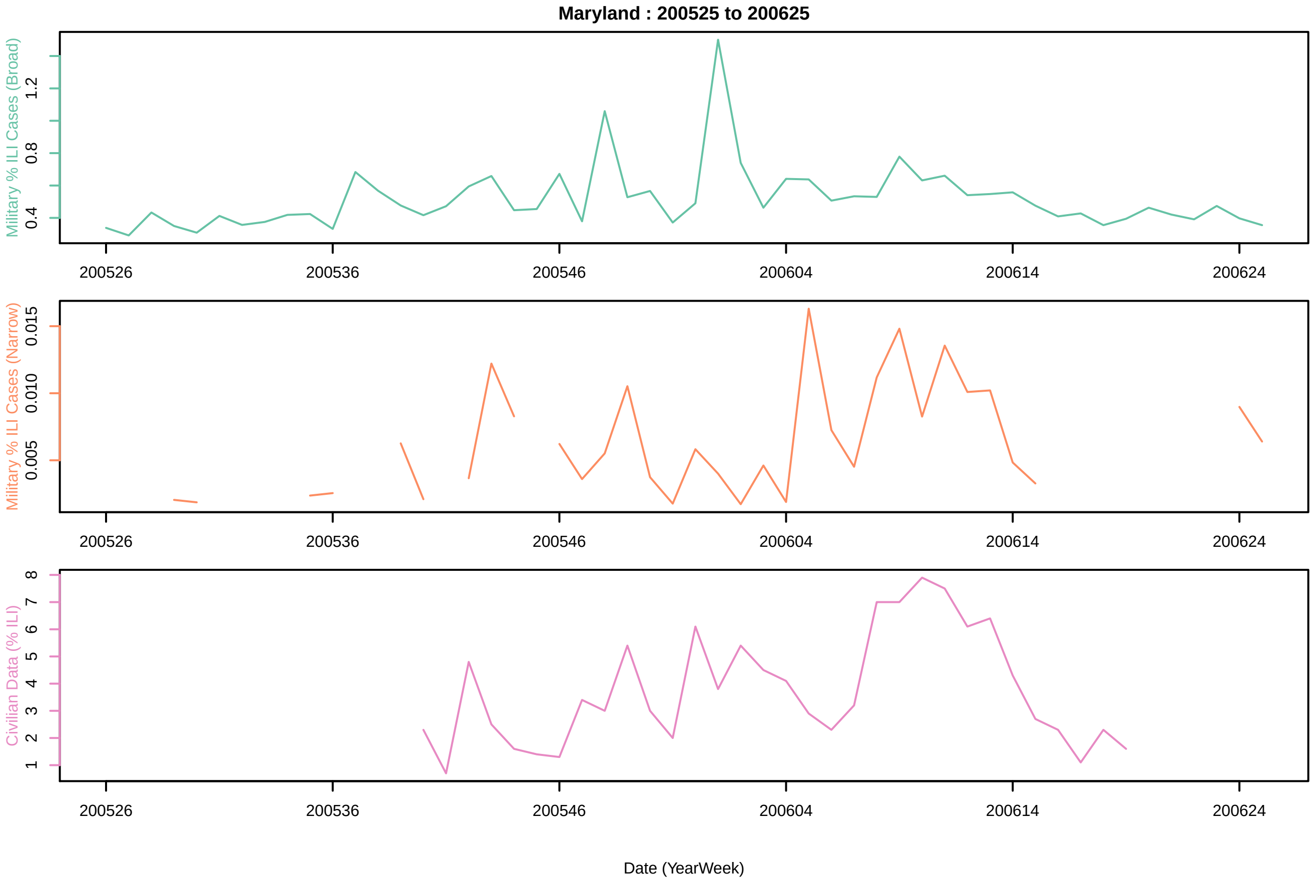

Maryland : 200525 to 200625
Military % ILI Cases (Broad)
Military % ILI Cases (Narrow)
Civilian Data (% ILI)
200526
200536
200546
200604
200614
200624
Date (YearWeek)

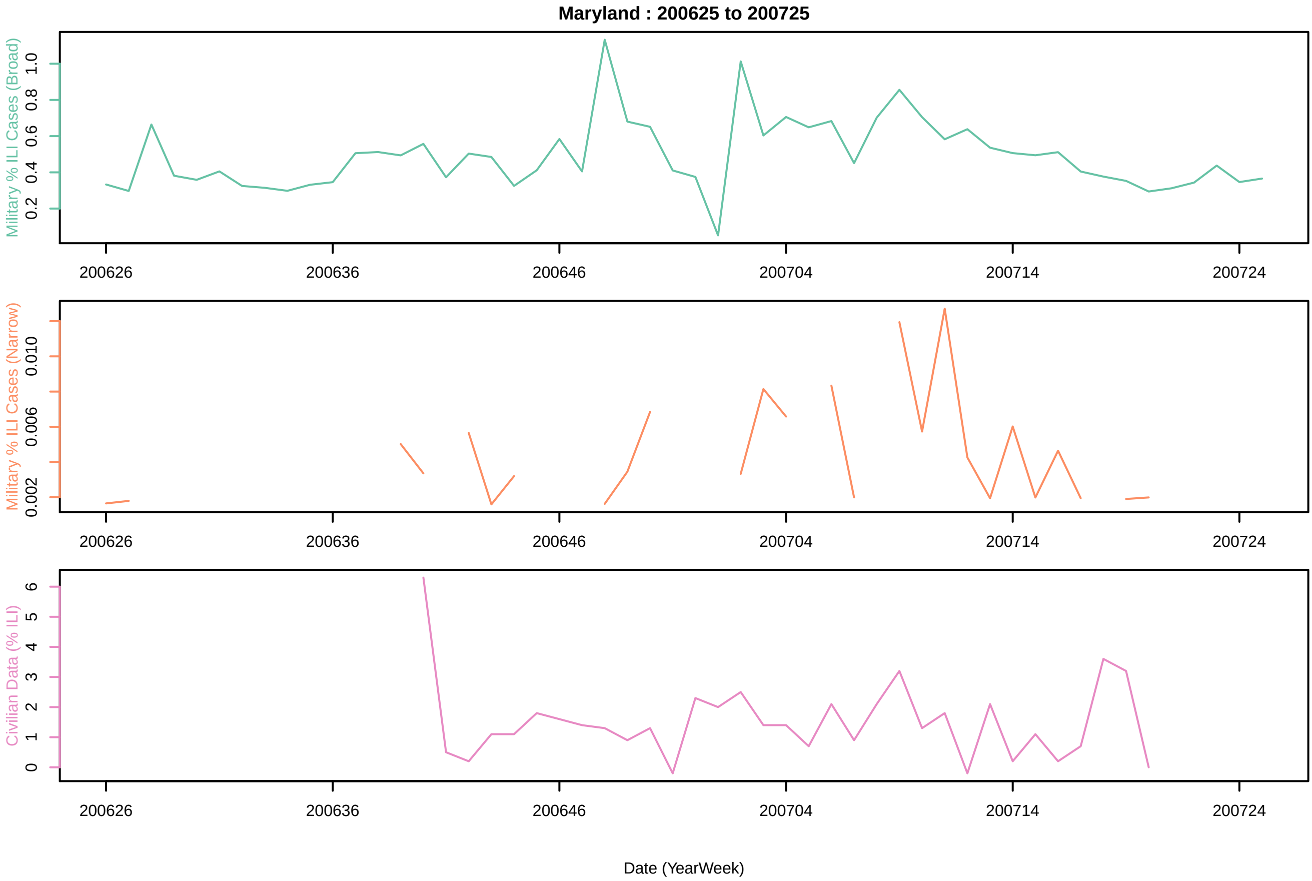

Maryland : 200625 to 200725
Military % ILI Cases (Broad)
0.2
0.4
0.6
0.8
1.0
200626
200636
200646
200704
200714
200724
Military % ILI Cases (Narrow)
0.002
0.006
0.010
200626
200636
200646
200704
200714
200724
Civilian Data (% ILI)
0
1
2
3
4
5
6
200626
200636
200646
200704
200714
200724
Date (YearWeek)

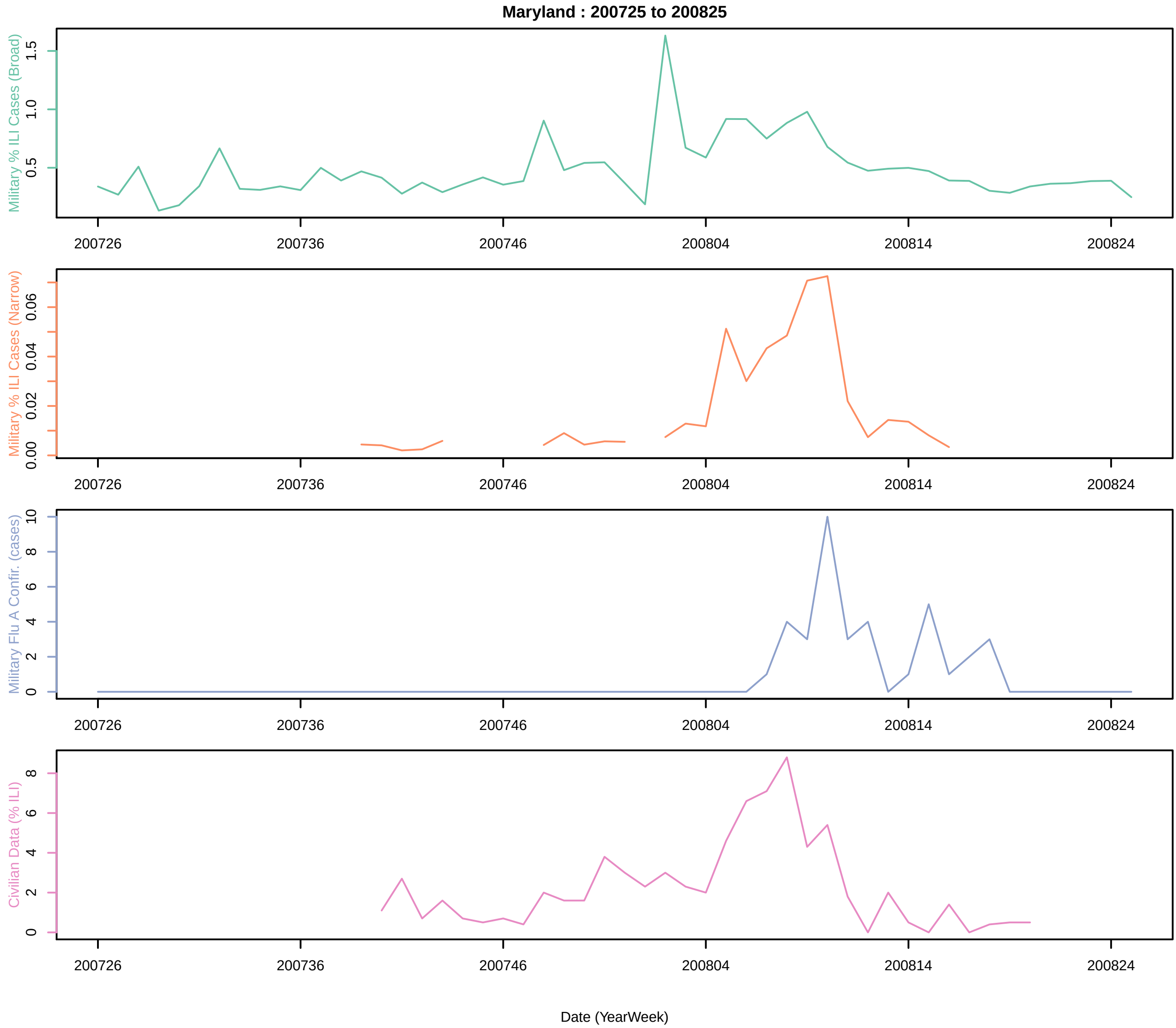
Maryland : 200725 to 200825
Military % ILI Cases (Broad)
Military % ILI Cases (Narrow)
Military Flu A Confir. (cases)
Civilian Data (% ILI)
200726
200736
200746
200804
200814
200824
Date (YearWeek)

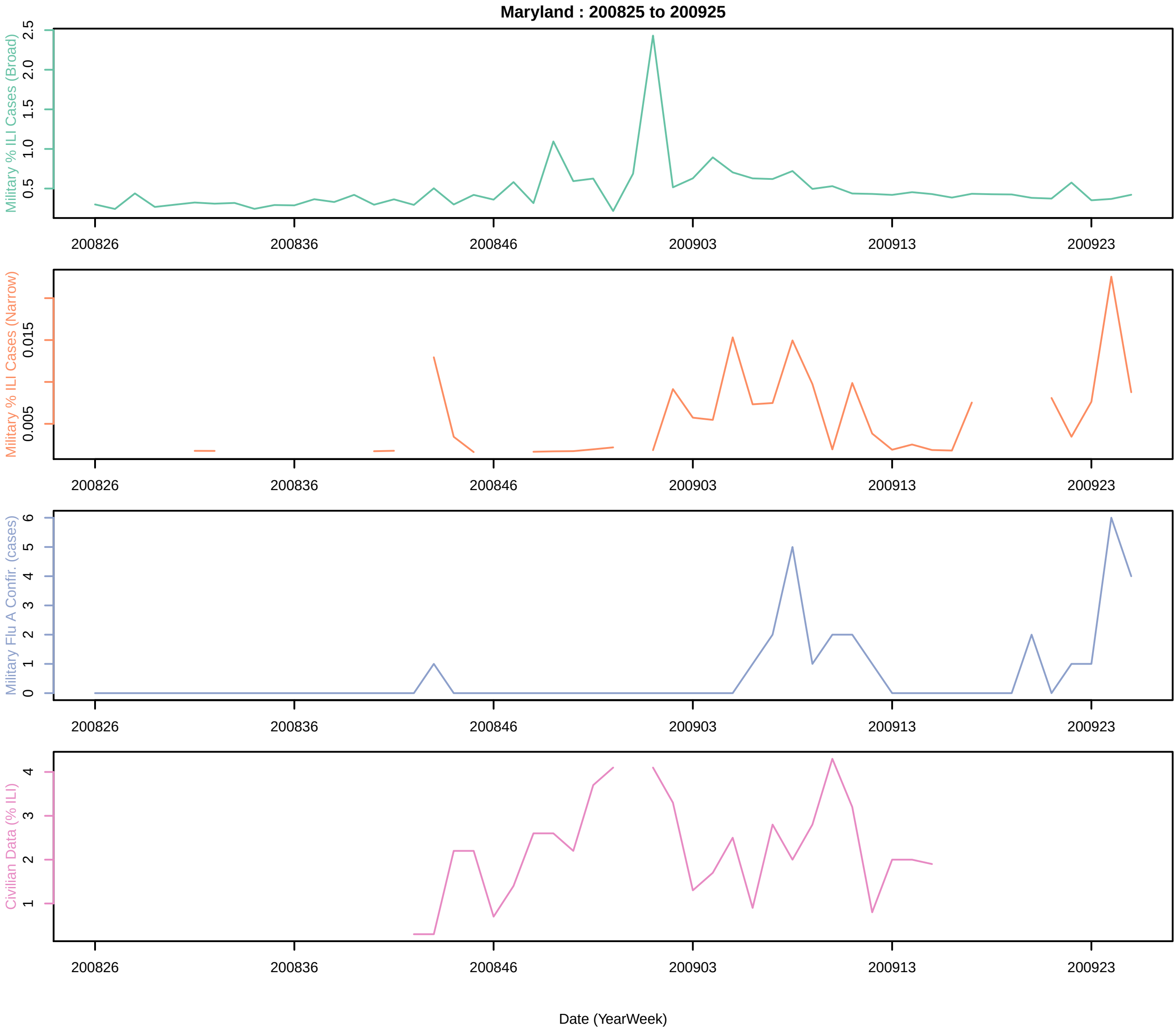
Maryland : 200825 to 200925
Military % ILI Cases (Broad)
0.5
1.0
1.5
2.0
2.5
Military % ILI Cases (Narrow)
0.005
0.015
Military Flu A Confir. (cases)
0
1
2
3
4
5
6
Civilian Data (% ILI)
1
2
3
4
200826
200836
200846
200903
200913
200923
Date (YearWeek)

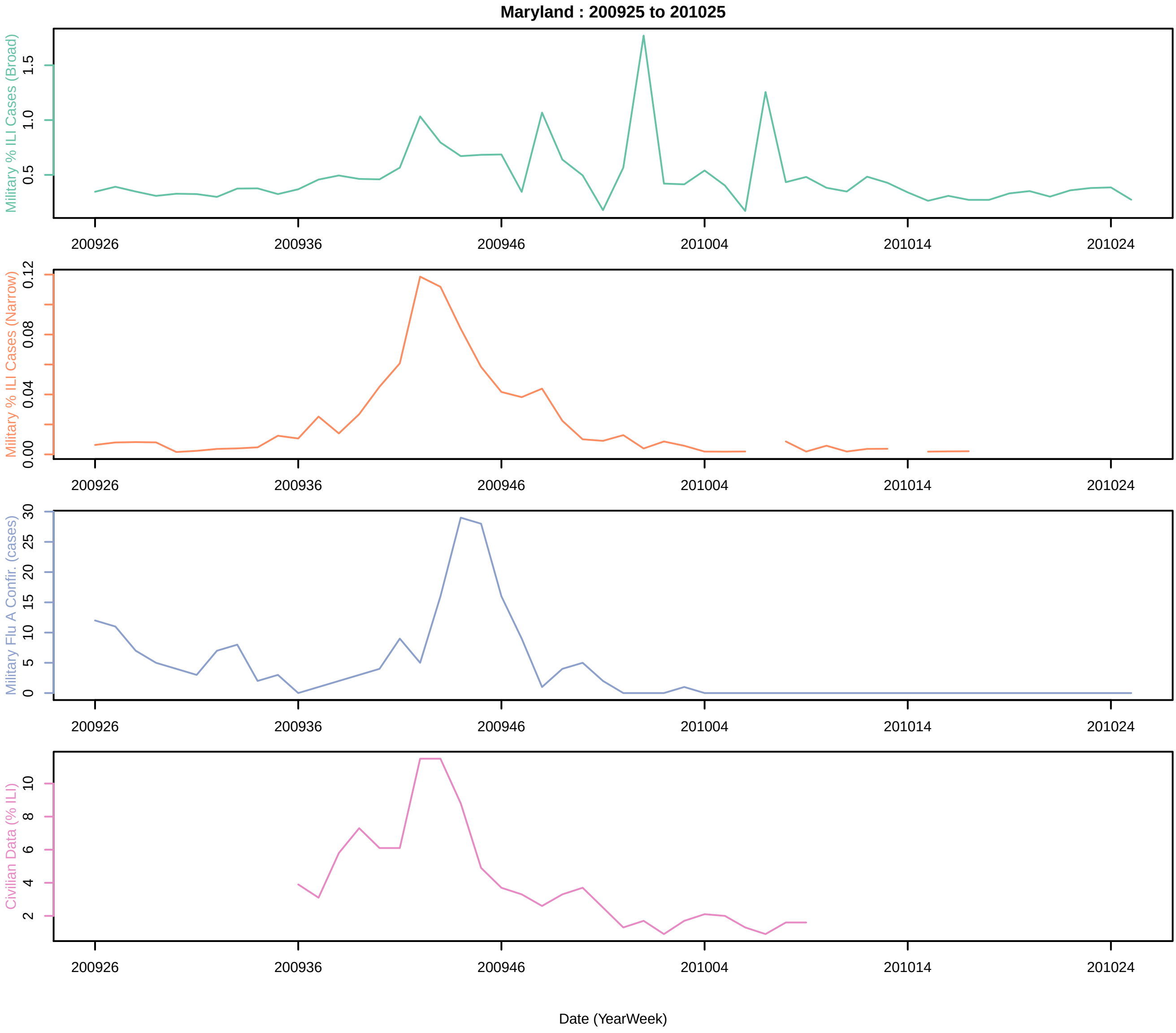

Maryland : 200925 to 201025
Military % ILI Cases (Broad)
0.5
1.0
1.5
Military % ILI Cases (Narrow)
0.00
0.04
0.08
0.12
Military Flu A Confir. (cases)
0
5
10
15
20
25
30
Civilian Data (% ILI)
2
4
6
8
10
200926
200936
200946
201004
201014
201024
Date (YearWeek)

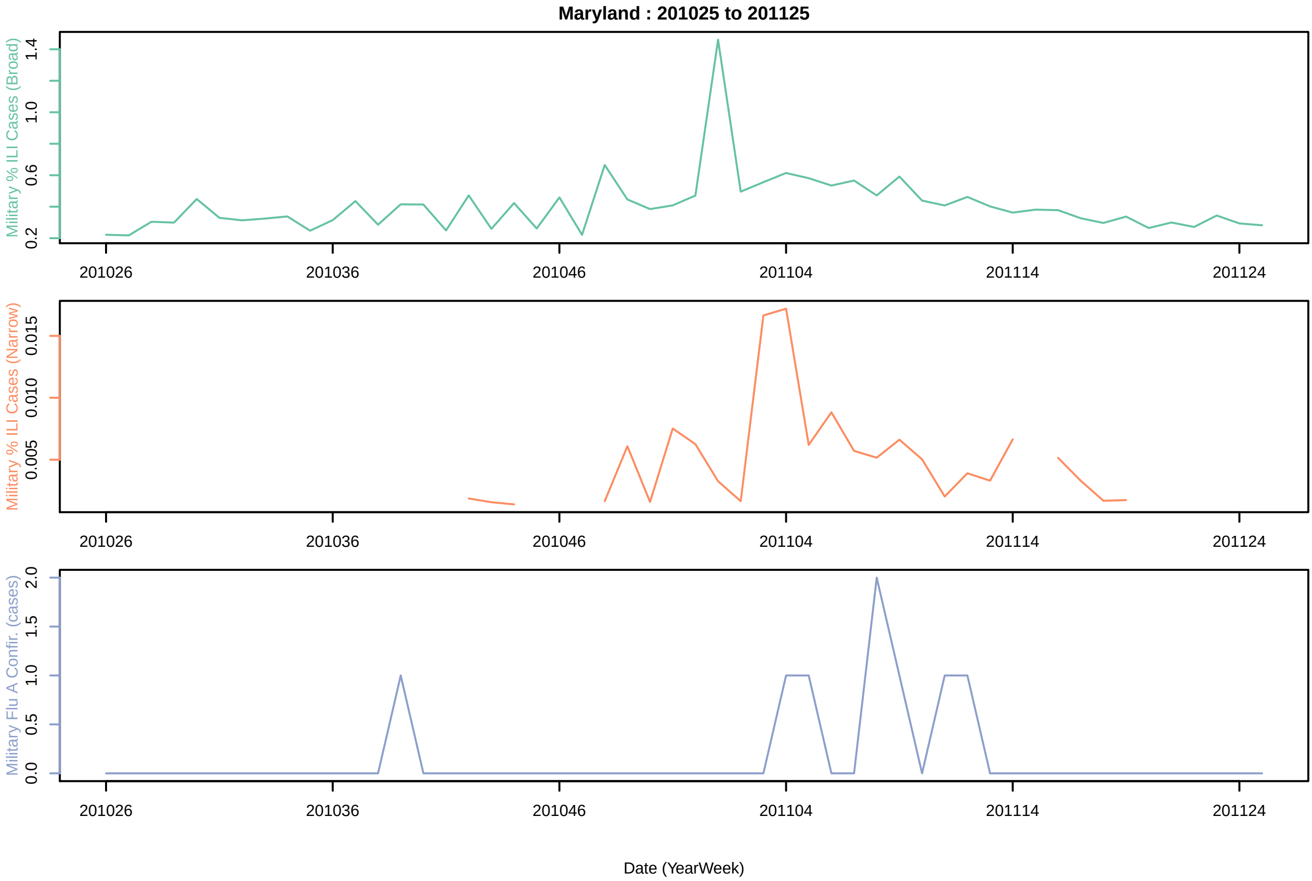

Maryland : 201025 to 201125
Military % ILI Cases (Broad)
0.2
0.6
1.0
1.4
Military % ILI Cases (Narrow)
0.005
0.010
0.015
Military Flu A Confir. (cases)
0.0
0.5
1.0
1.5
2.0
201026
201036
201046
201104
201114
201124
Date (YearWeek)

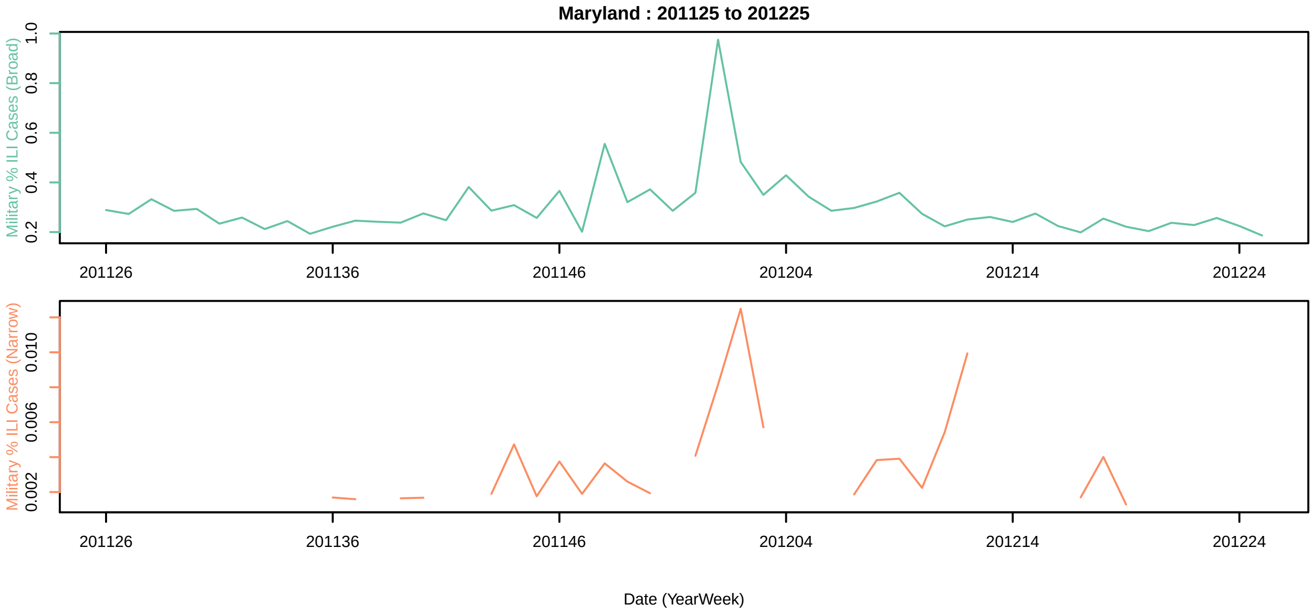
Maryland : 201125 to 201225
Military % ILI Cases (Broad)
0.2
0.4
0.6
0.8
1.0
201126
201136
201146
201204
201214
201224
Military % ILI Cases (Narrow)
0.002
0.006
0.010
201126
201136
201146
201204
201214
201224
Date (YearWeek)

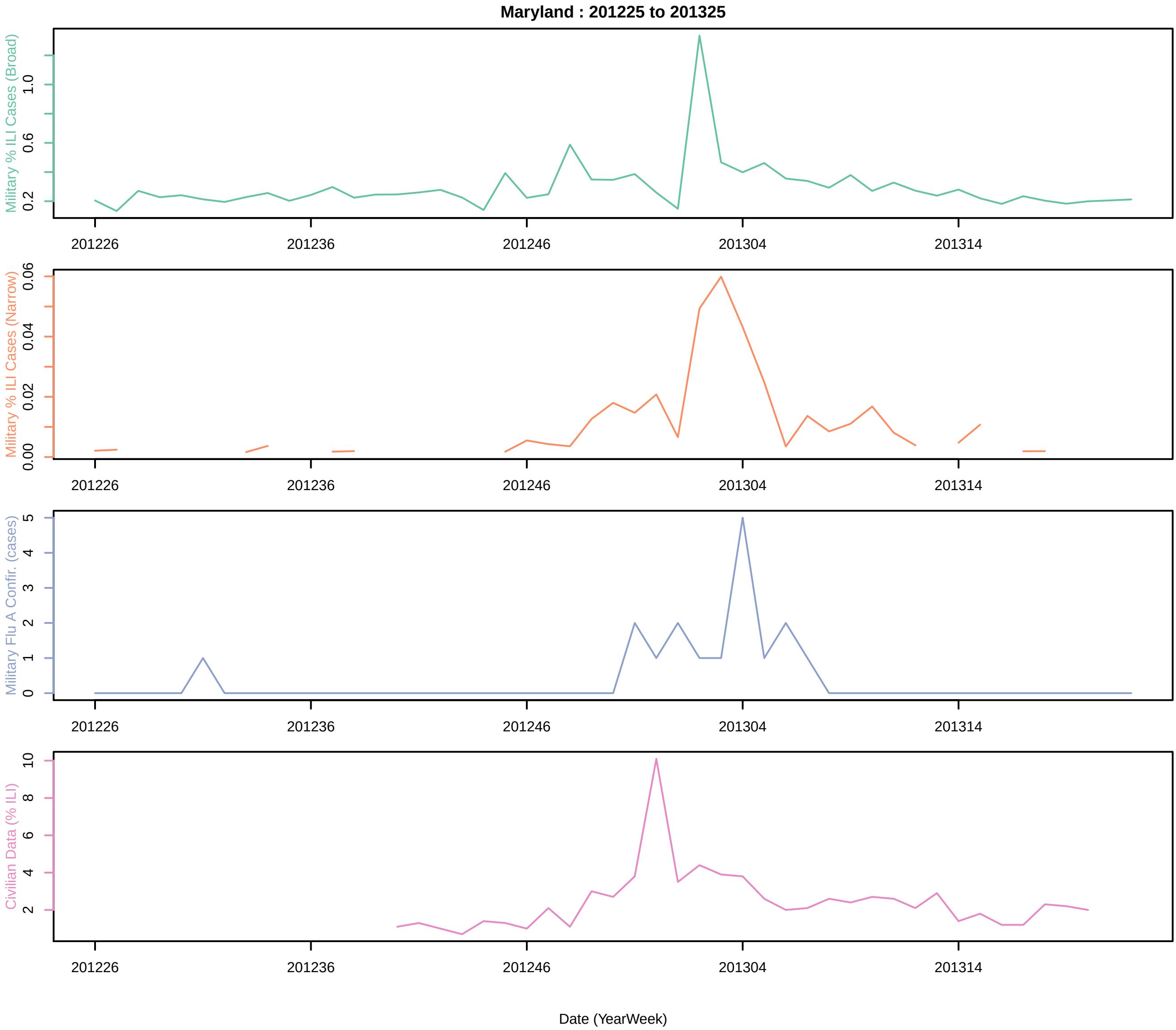

Maryland : 201225 to 201325
Military % ILI Cases (Broad)
0.2
0.6
1.0
Military % ILI Cases (Narrow)
0.00
0.02
0.04
0.06
Military Flu A Confir. (cases)
0
1
2
3
4
5
Civilian Data (% ILI)
2
4
6
8
10
201226
201236
201246
201304
201314
Date (YearWeek)

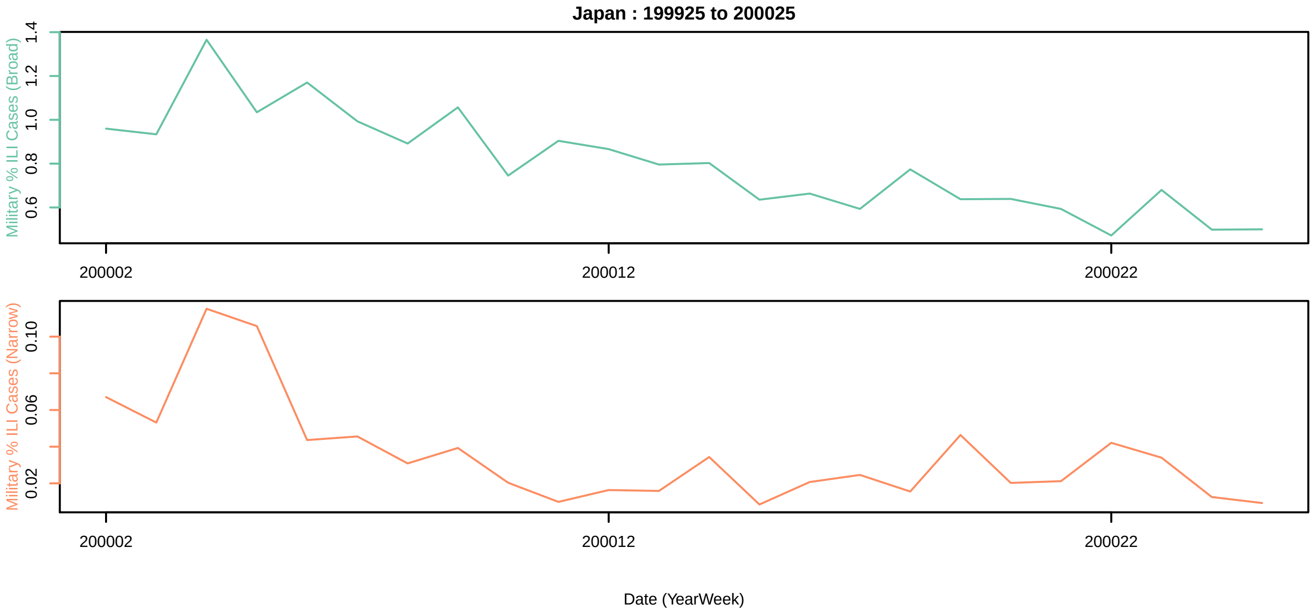
Japan : 199925 to 200025
Military % ILI Cases (Broad)
0.6
0.8
1.0
1.2
1.4
200002
200012
200022
Military % ILI Cases (Narrow)
0.02
0.06
0.10
200002
200012
200022
Date (YearWeek)

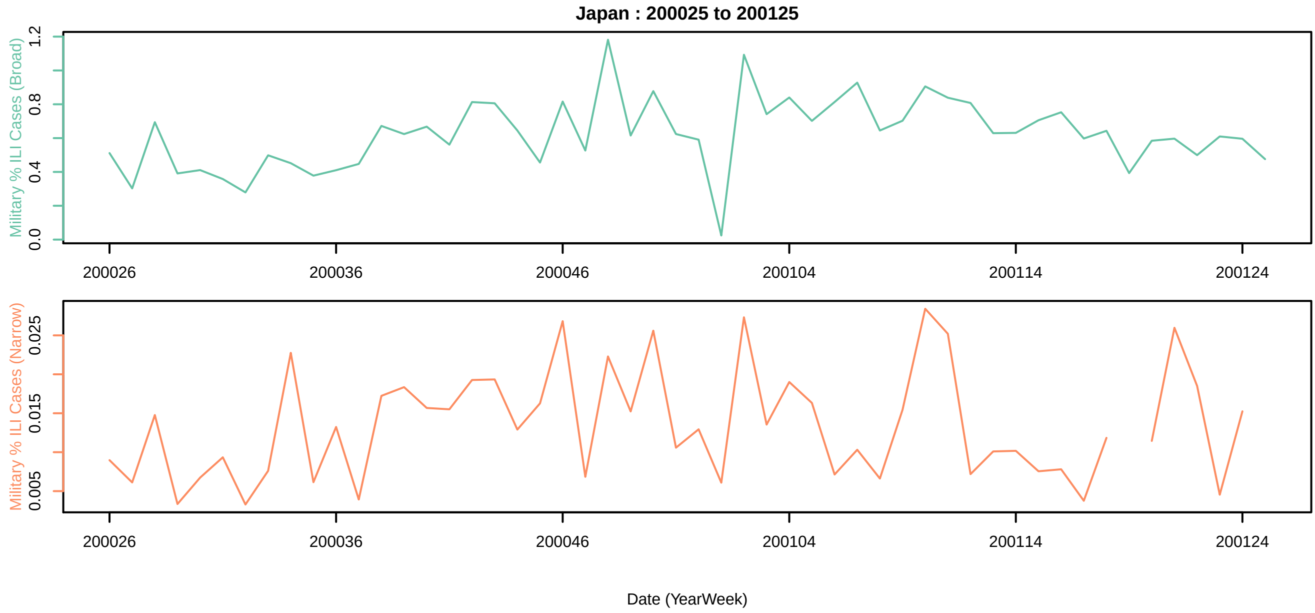

Japan : 200025 to 200125
Military % ILI Cases (Broad)
0.0
0.4
0.8
1.2
200026
200036
200046
200104
200114
200124
Military % ILI Cases (Narrow)
0.005
0.015
0.025
200026
200036
200046
200104
200114
200124
Date (YearWeek)

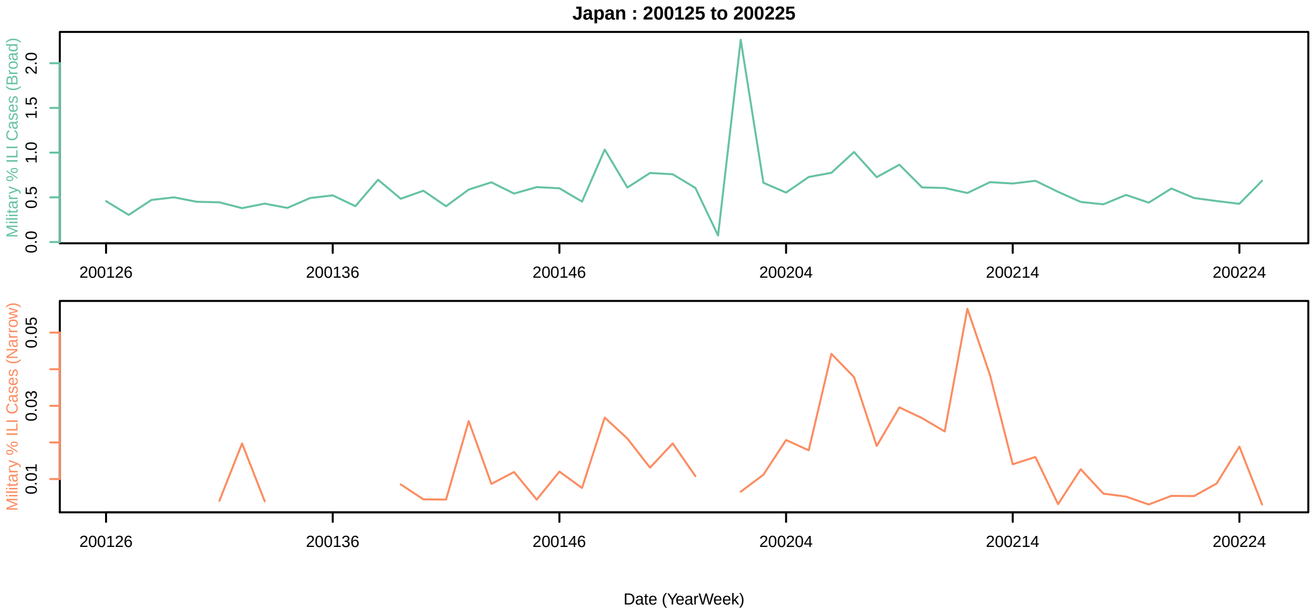
Japan : 200125 to 200225
Military % ILI Cases (Broad)
0.0
0.5
1.0
1.5
2.0
200126
200136
200146
200204
200214
200224
Military % ILI Cases (Narrow)
0.01
0.03
0.05
200126
200136
200146
200204
200214
200224
Date (YearWeek)

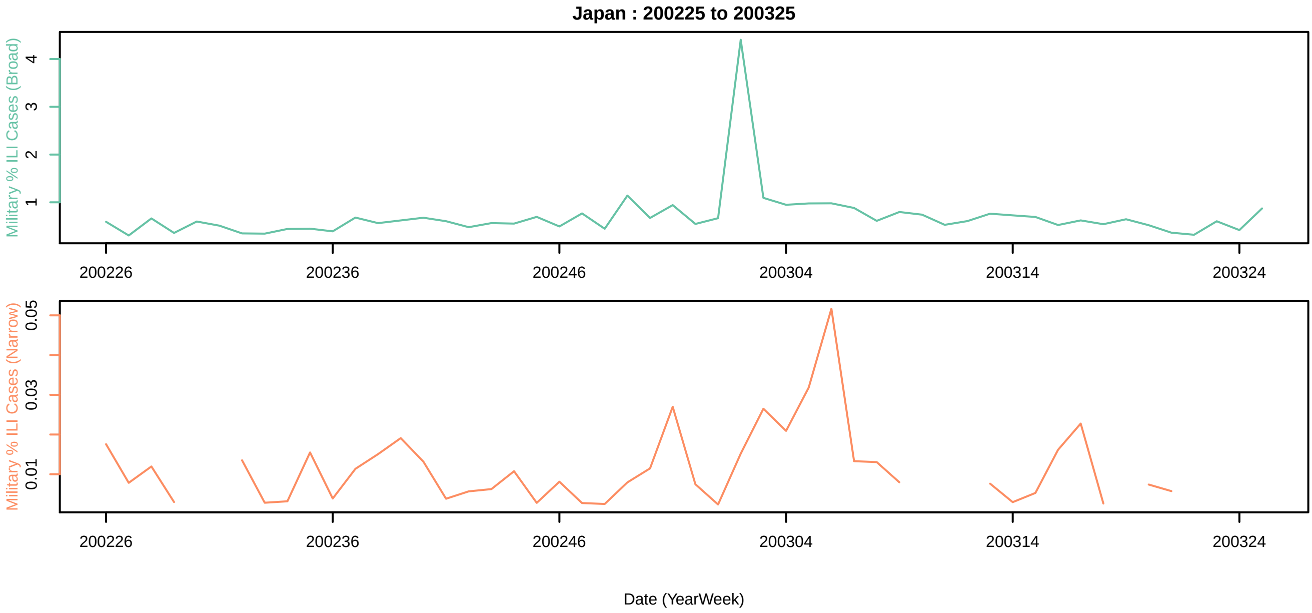
Japan : 200225 to 200325
Military % ILI Cases (Broad)
1
2
3
4
200226
200236
200246
200304
200314
200324
Military % ILI Cases (Narrow)
0.01
0.03
0.05
200226
200236
200246
200304
200314
200324
Date (YearWeek)

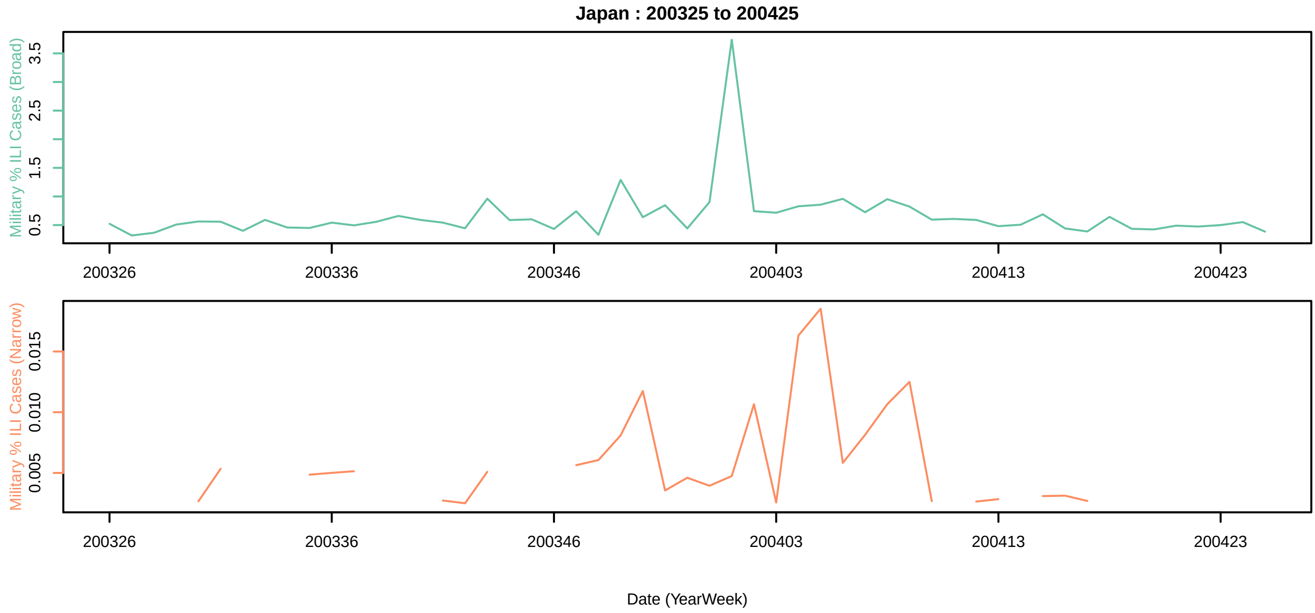
Japan : 200325 to 200425
Military % ILI Cases (Broad)
0.5
1.5
2.5
3.5
Military % ILI Cases (Narrow)
0.005
0.010
0.015
200326
200336
200346
200403
200413
200423
Date (YearWeek)

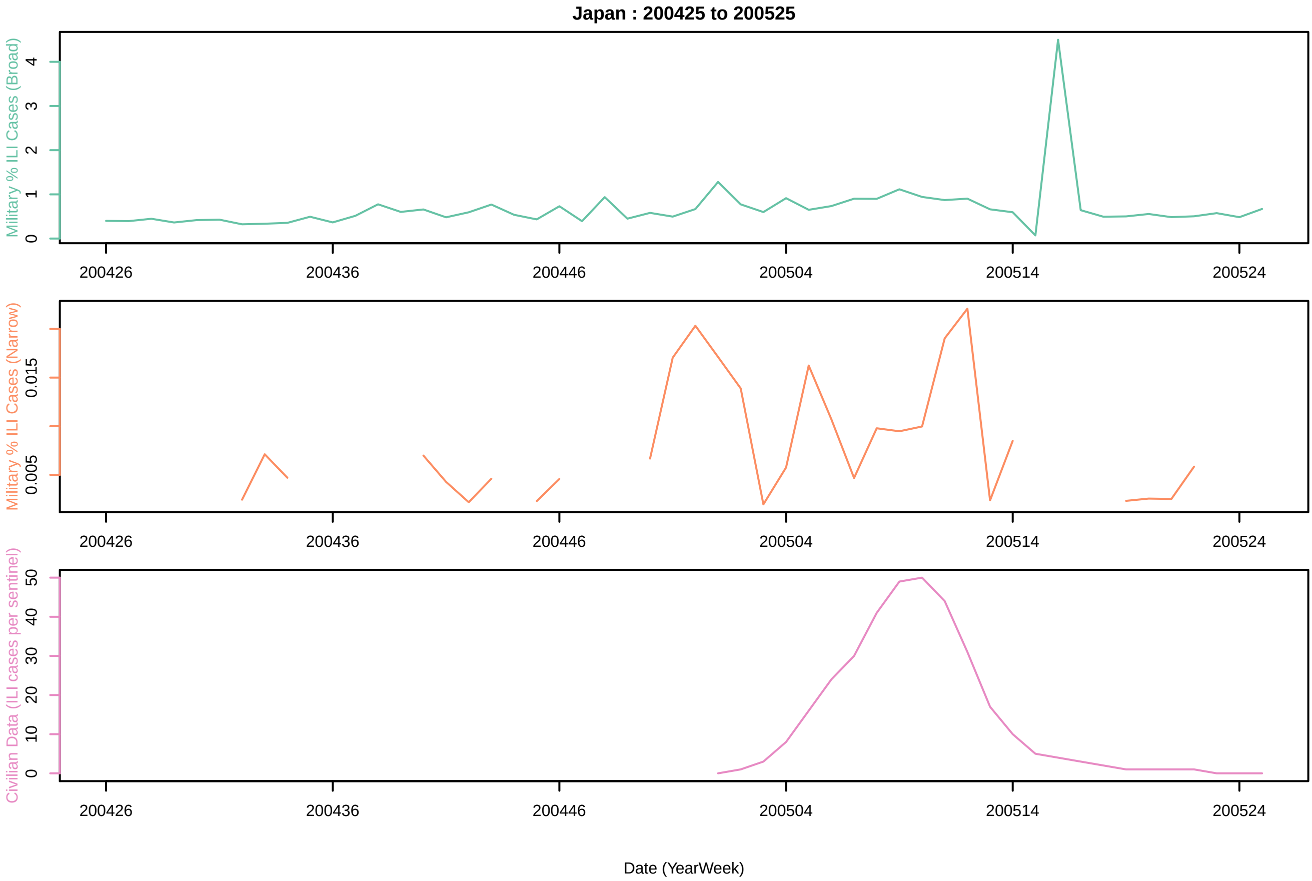
Japan : 200425 to 200525
Military % ILI Cases (Broad)
0
1
2
3
4
Military % ILI Cases (Narrow)
0.005
0.015
Civilian Data (ILI cases per sentinel)
0
10
20
30
40
50
200426
200436
200446
200504
200514
200524
Date (YearWeek)

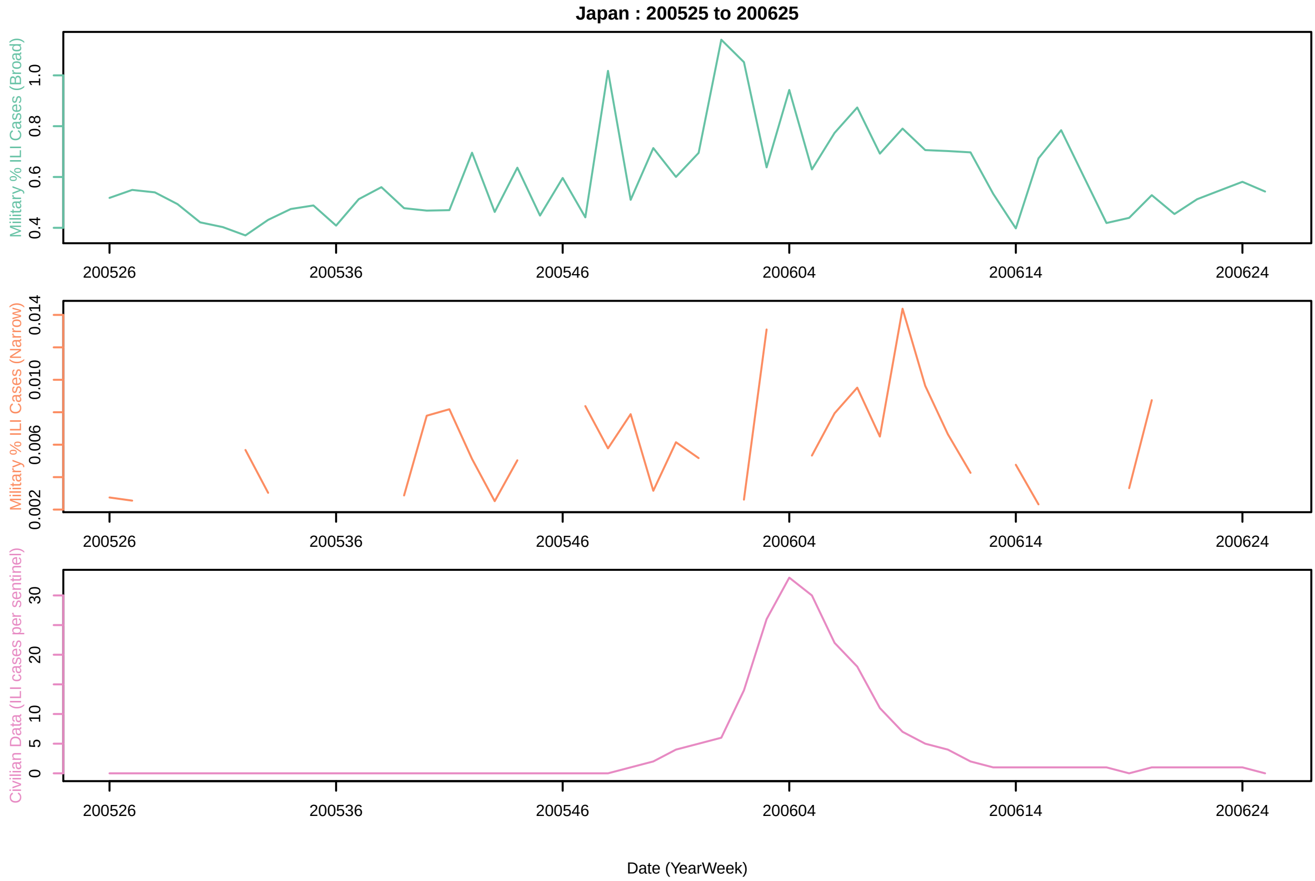

Japan : 200525 to 200625
Military % ILI Cases (Broad)
0.4
0.6
0.8
1.0
Military % ILI Cases (Narrow)
0.002
0.006
0.010
0.014
Civilian Data (ILI cases per sentinel)
0
5
10
20
30
200526
200536
200546
200604
200614
200624
Date (YearWeek)

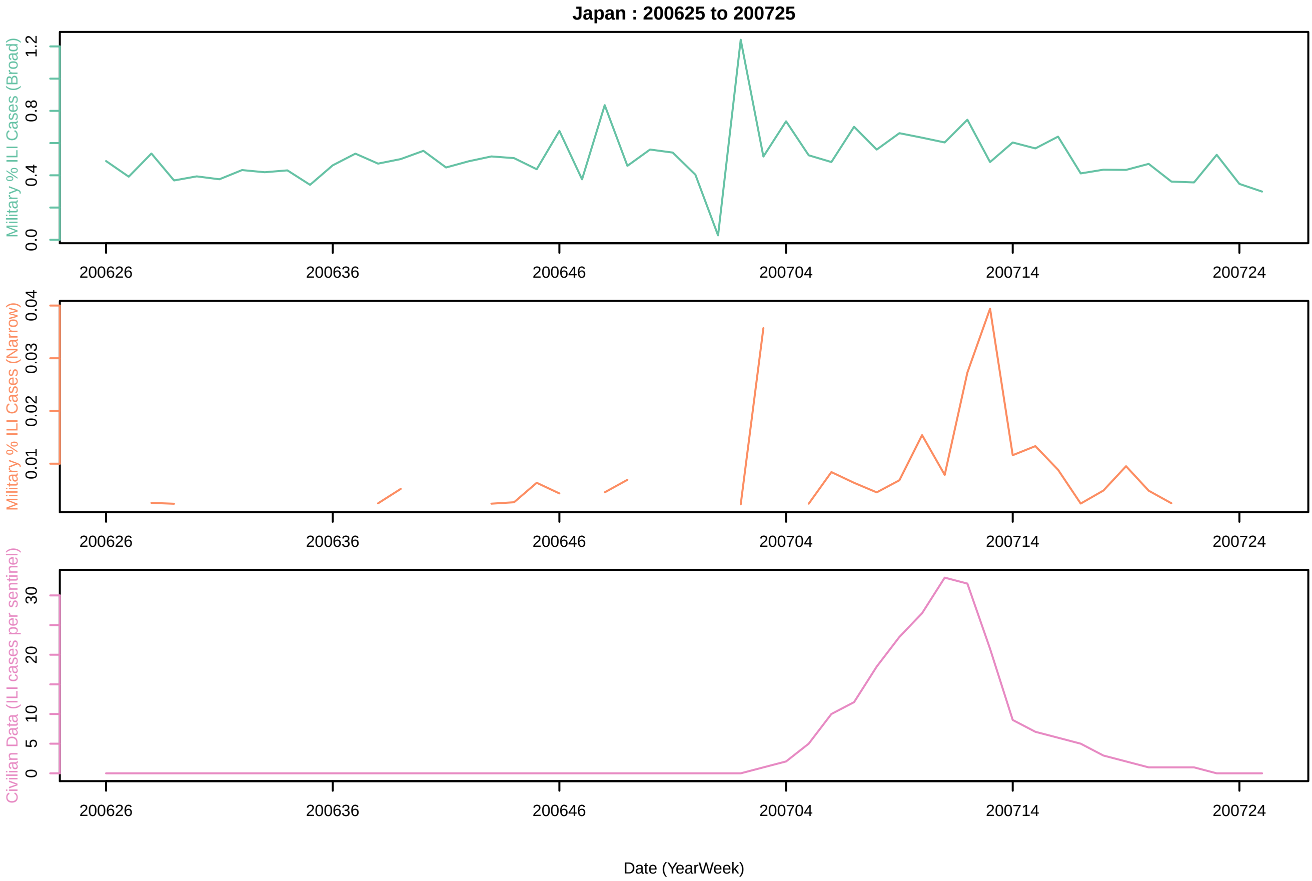
Japan : 200625 to 200725
Military % ILI Cases (Broad)
0.0
0.4
0.8
1.2
Military % ILI Cases (Narrow)
0.01
0.02
0.03
0.04
Civilian Data (ILI cases per sentinel)
0
5
10
20
30
200626
200636
200646
200704
200714
200724
Date (YearWeek)

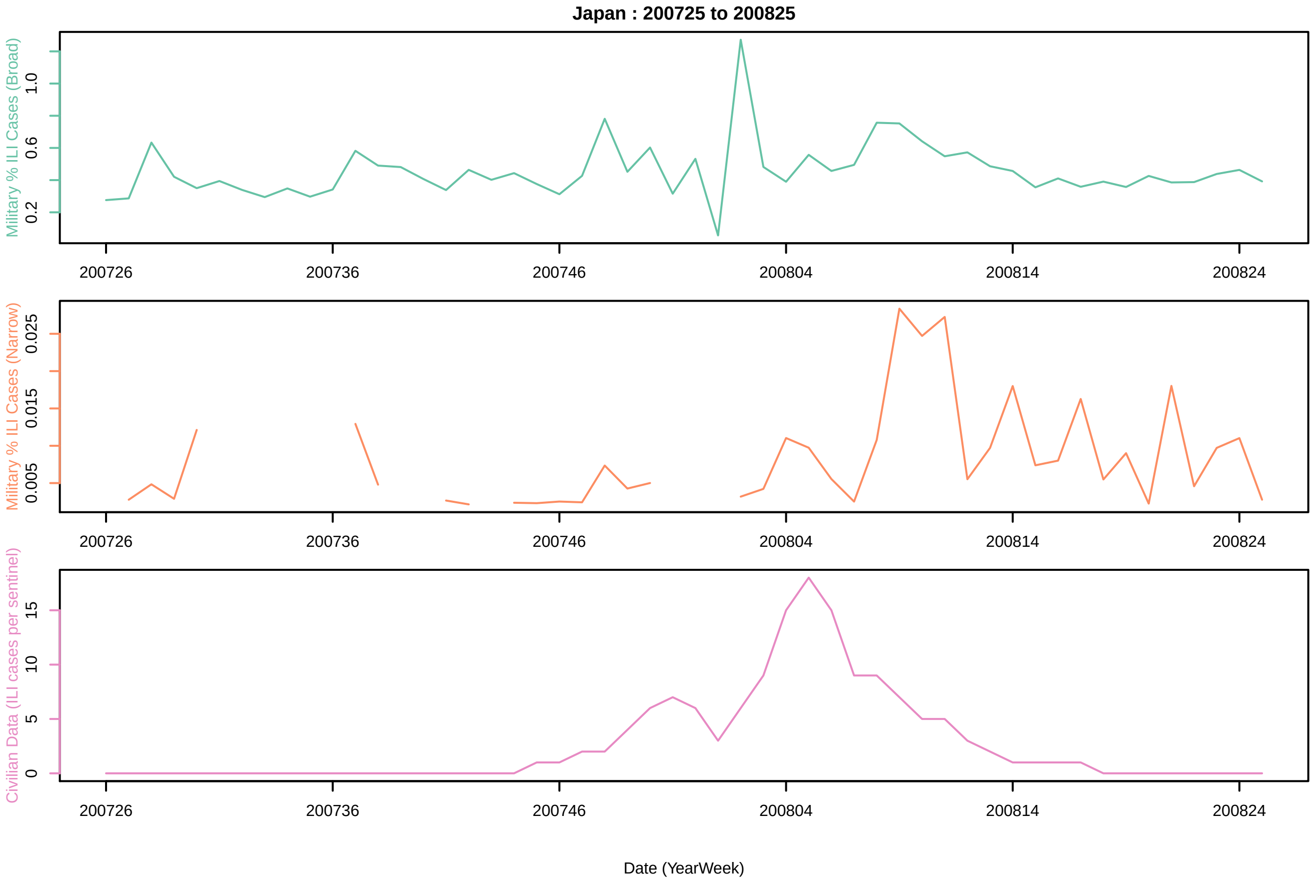
Japan : 200725 to 200825
Military % ILI Cases (Broad)
0.2
0.6
1.0
200726
200736
200746
200804
200814
200824
Military % ILI Cases (Narrow)
0.005
0.015
0.025
Civilian Data (ILI cases per sentinel)
0
5
10
15
Date (YearWeek)

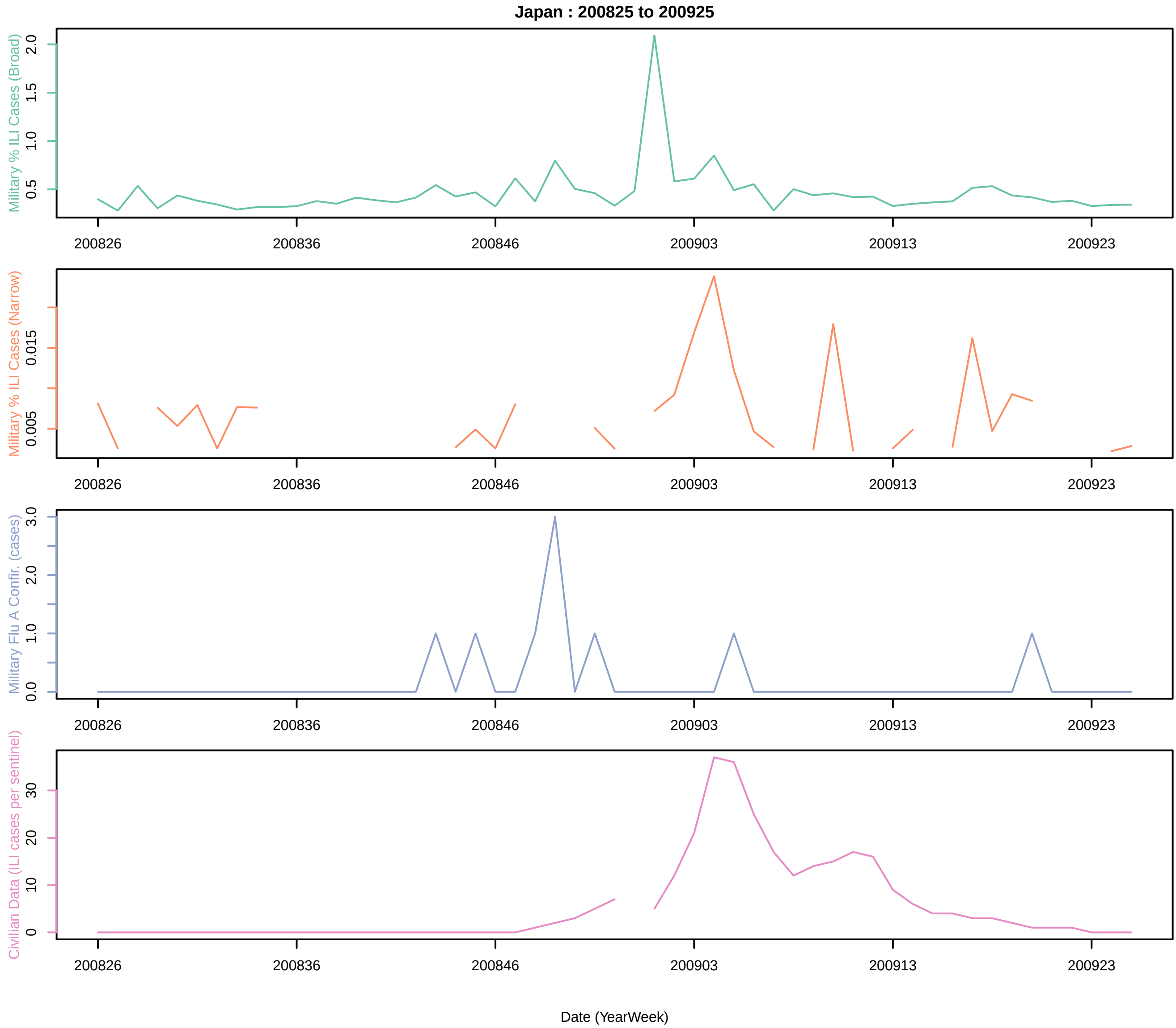
Japan : 200825 to 200925
Military % ILI Cases (Broad)
0.5
1.0
1.5
2.0
Military % ILI Cases (Narrow)
0.005
0.015
Military Flu A Confir. (cases)
0.0
1.0
2.0
3.0
Civilian Data (ILI cases per sentinel)
0
10
20
30
200826
200836
200846
200903
200913
200923
Date (YearWeek)

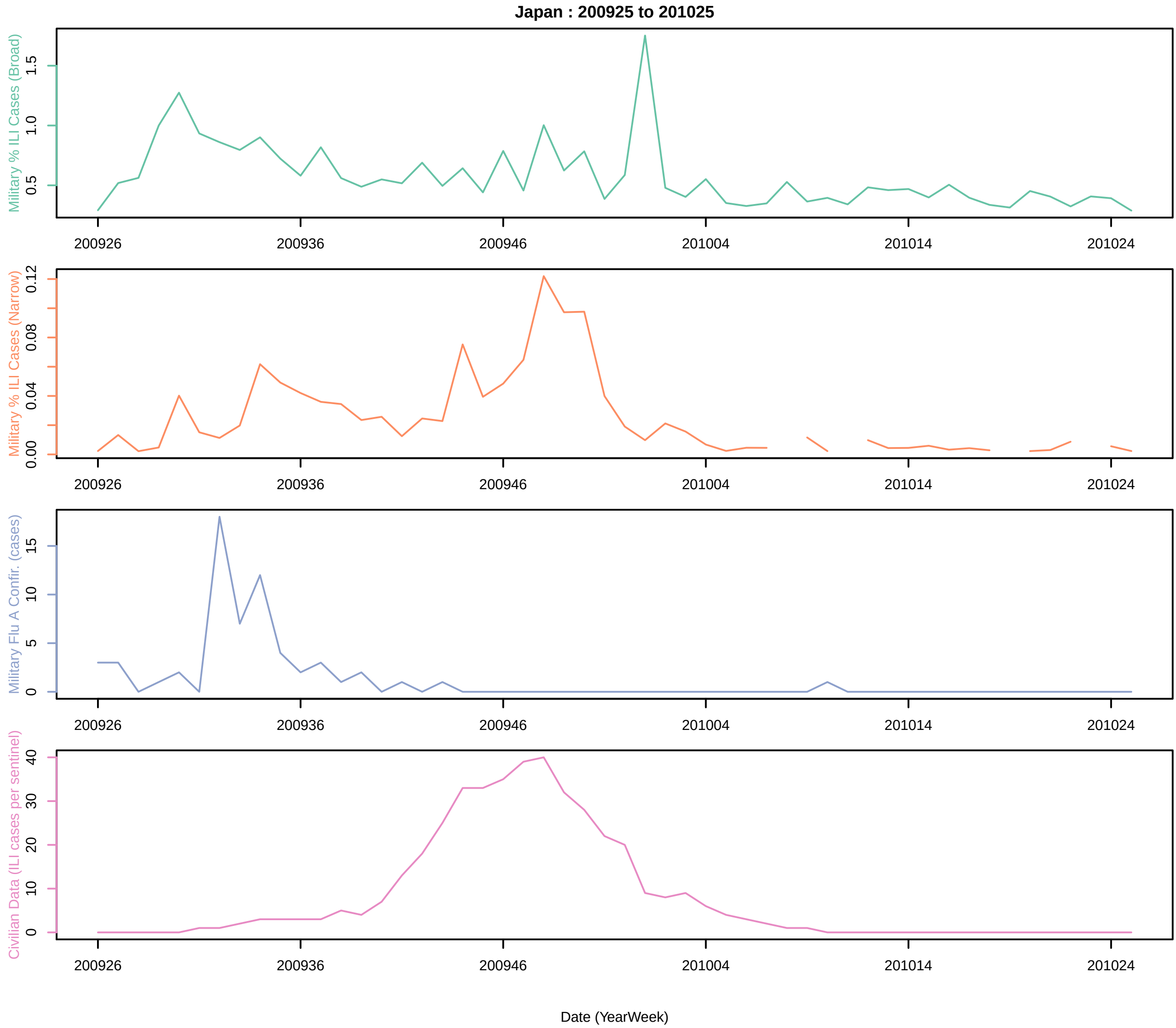
Japan : 200925 to 201025
Military % ILI Cases (Broad)
0.5
1.0
1.5
Military % ILI Cases (Narrow)
0.00
0.04
0.08
0.12
Military Flu A Confir. (cases)
0
5
10
15
Civilian Data (ILI cases per sentinel)
0
10
20
30
40
200926
200936
200946
201004
201014
201024
Date (YearWeek)

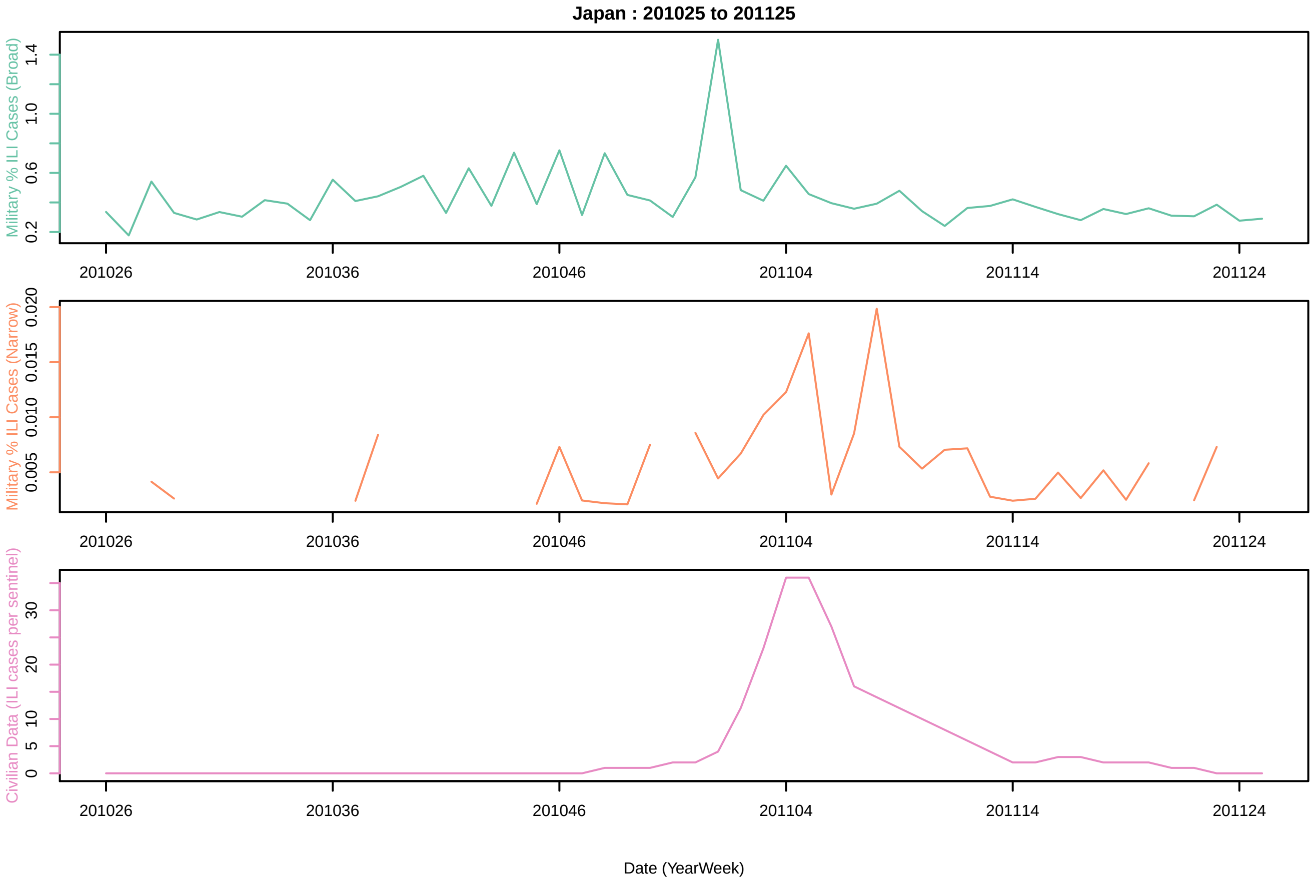
Japan : 201025 to 201125
Military % ILI Cases (Broad)
0.2
0.6
1.0
1.4
Military % ILI Cases (Narrow)
0.005
0.010
0.015
0.020
Civilian Data (ILI cases per sentinel)
0
5
10
20
30
201026
201036
201046
201104
201114
201124
Date (YearWeek)

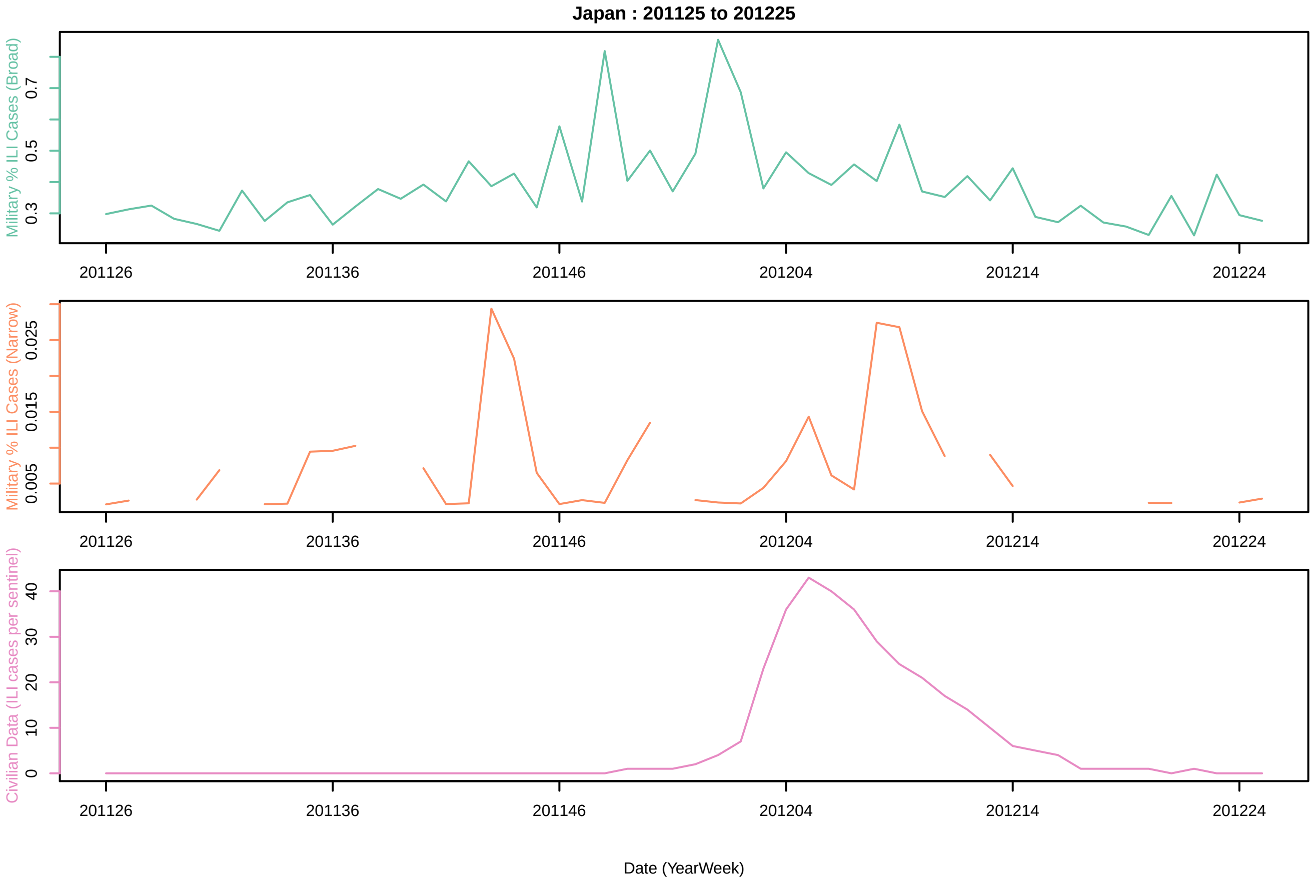

Japan : 201125 to 201225
Military % ILI Cases (Broad)
0.3
0.5
0.7
201126
201136
201146
201204
201214
201224
Military % ILI Cases (Narrow)
0.005
0.015
0.025
Civilian Data (ILI cases per sentinel)
0
10
20
30
40
Date (YearWeek)

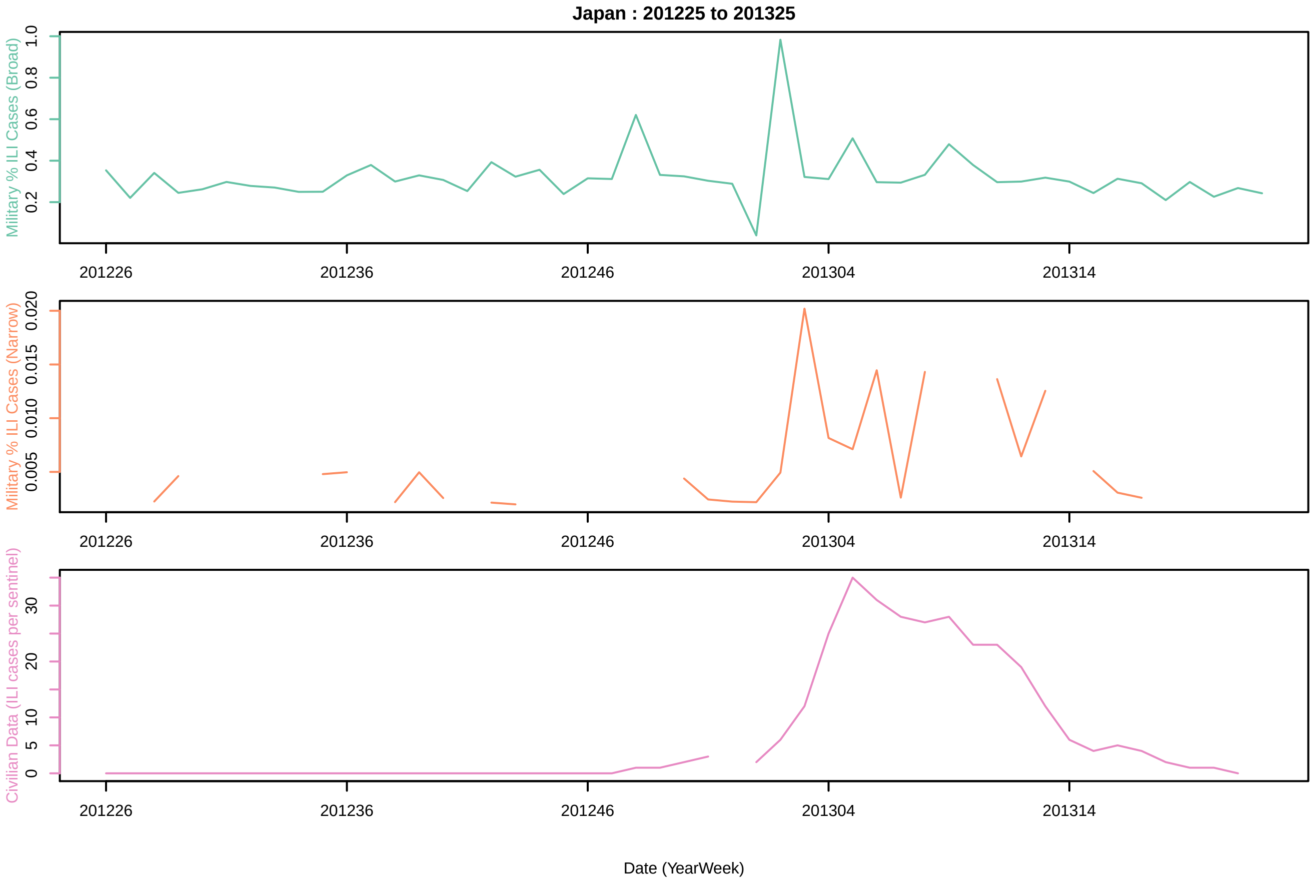

Japan : 201225 to 201325
Military % ILI Cases (Broad)
1.0
0.8
0.6
0.4
0.2
201226
201236
201246
201304
201314
Military % ILI Cases (Narrow)
0.020
0.015
0.010
0.005
201226
201236
201246
201304
201314
Civilian Data (ILI cases per sentinel)
30
20
10
5
0
201226
201236
201246
201304
201314
Date (YearWeek)

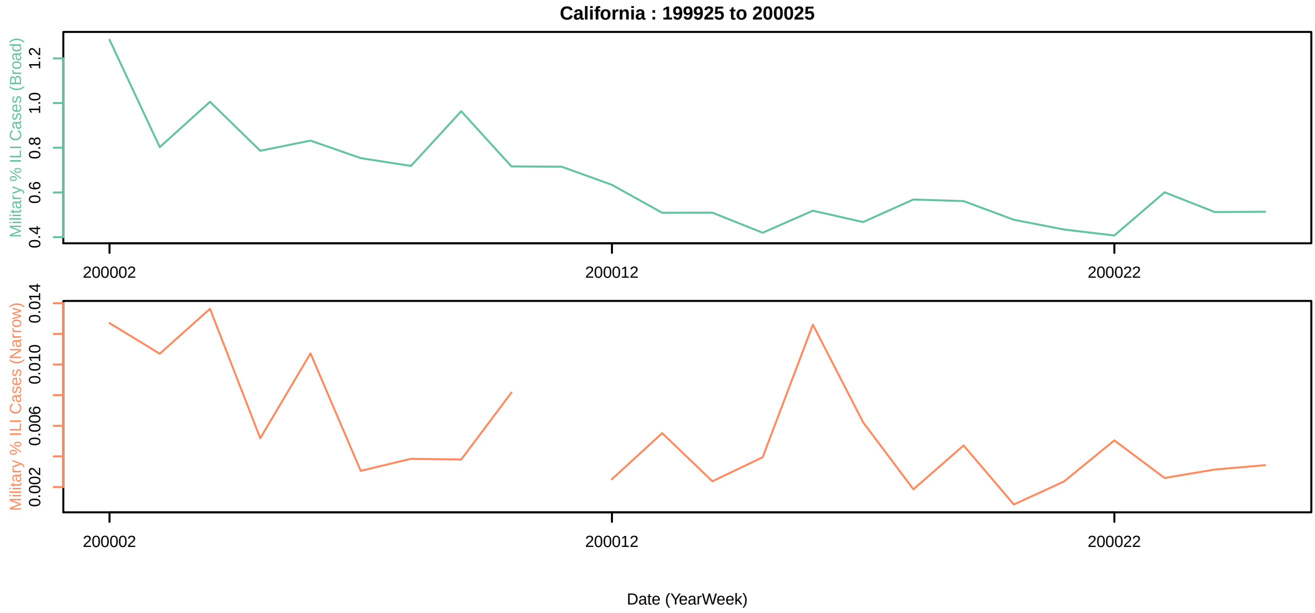
California : 199925 to 200025
Military % ILI Cases (Broad)
0.4
0.6
0.8
1.0
1.2
200002
200012
200022
Military % ILI Cases (Narrow)
0.002
0.006
0.010
0.014
200002
200012
200022
Date (YearWeek)

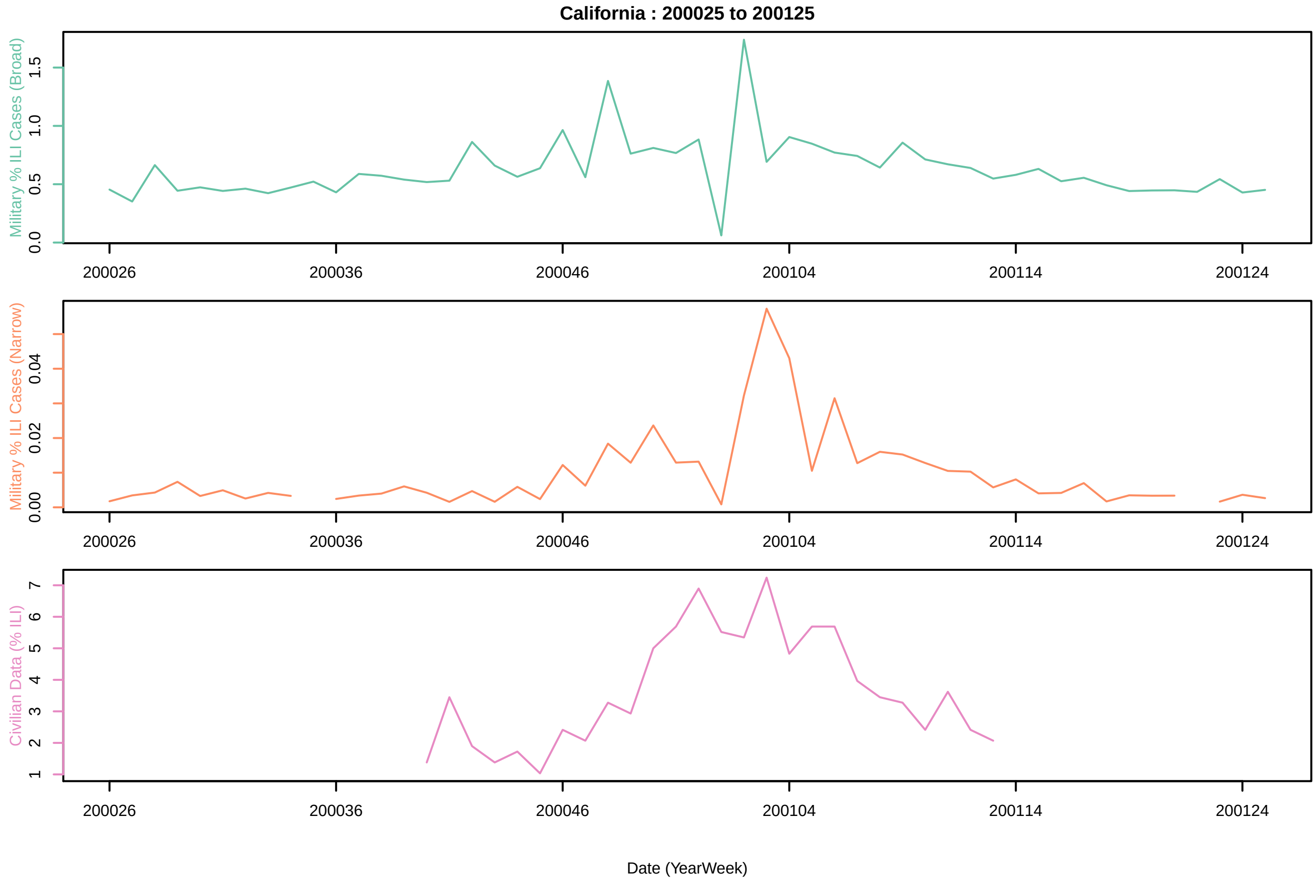
California : 200025 to 200125
Military % ILI Cases (Broad)
0.0
0.5
1.0
1.5
Military % ILI Cases (Narrow)
0.00
0.02
0.04
Civilian Data (% ILI)
1
2
3
4
5
6
7
200026
200036
200046
200104
200114
200124
Date (YearWeek)

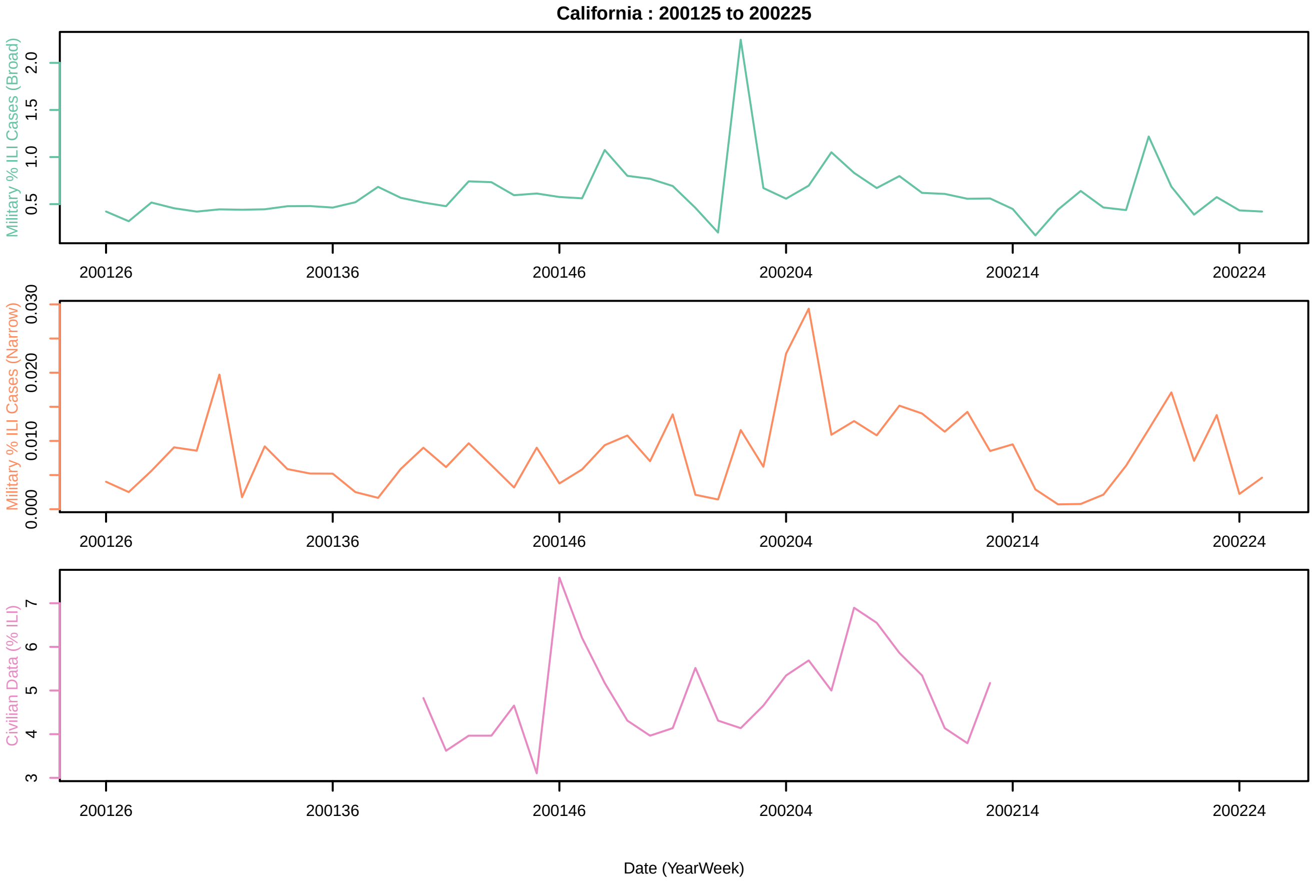
California : 200125 to 200225
Military % ILI Cases (Broad)
0.5
1.0
1.5
2.0
Military % ILI Cases (Narrow)
0.000
0.010
0.020
0.030
Civilian Data (% ILI)
3
4
5
6
7
200126
200136
200146
200204
200214
200224
Date (YearWeek)

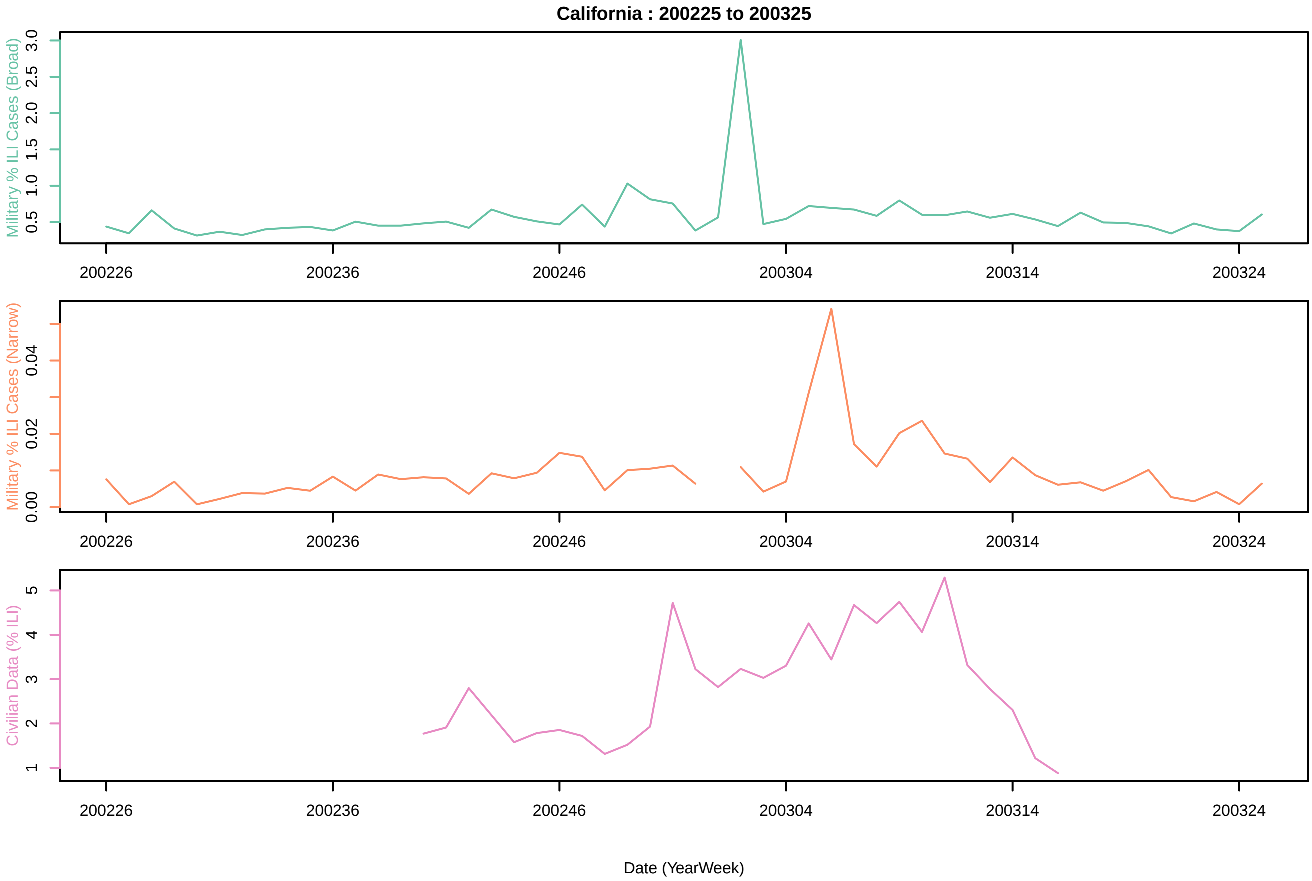

California : 200225 to 200325
Military % ILI Cases (Broad)
0.5
1.0
1.5
2.0
2.5
3.0
Military % ILI Cases (Narrow)
0.00
0.02
0.04
Civilian Data (% ILI)
1
2
3
4
5
200226
200236
200246
200304
200314
200324
Date (YearWeek)

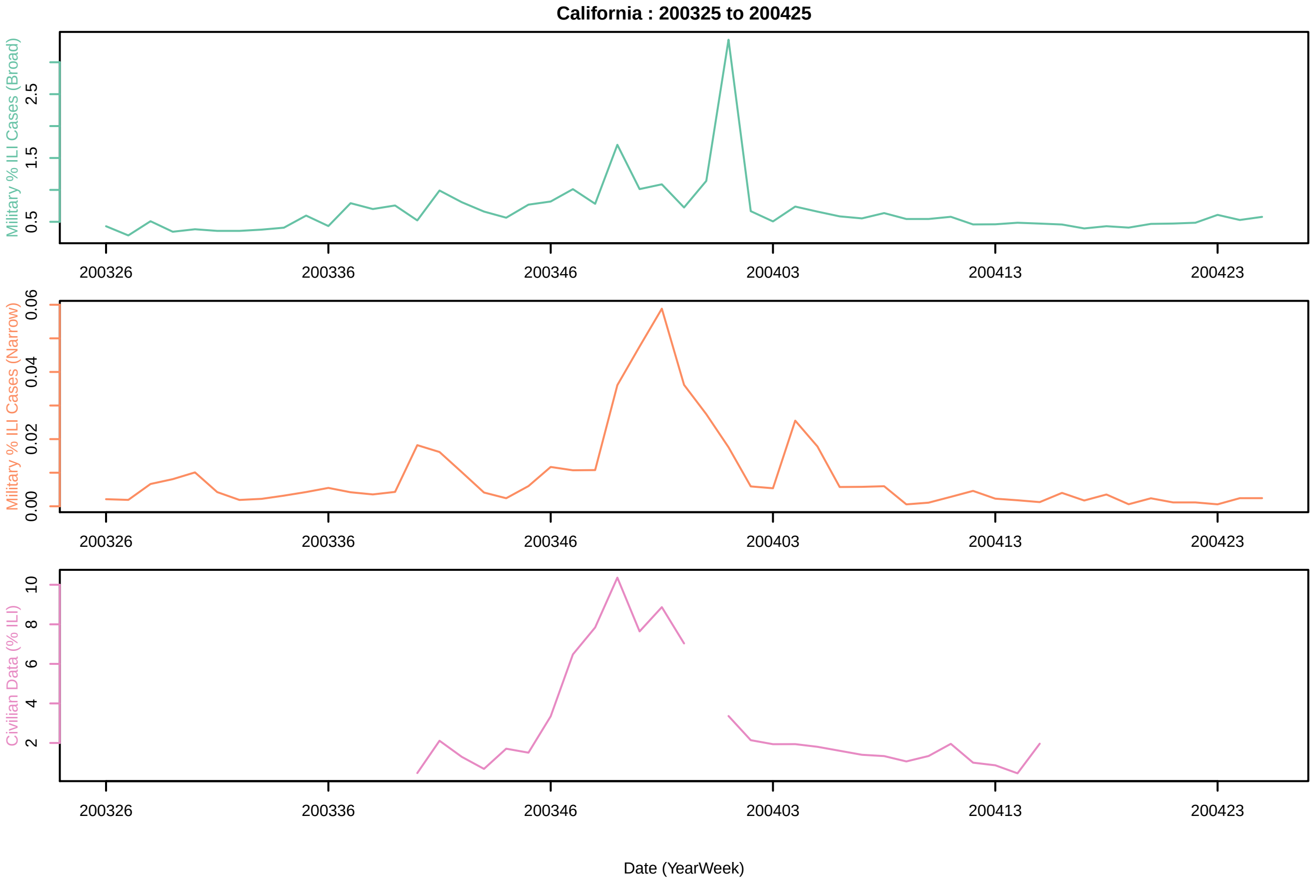

California : 200325 to 200425
Military % ILI Cases (Broad)
0.5
1.5
2.5
Military % ILI Cases (Narrow)
0.00
0.02
0.04
0.06
Civilian Data (% ILI)
2
4
6
8
10
200326
200336
200346
200403
200413
200423
Date (YearWeek)

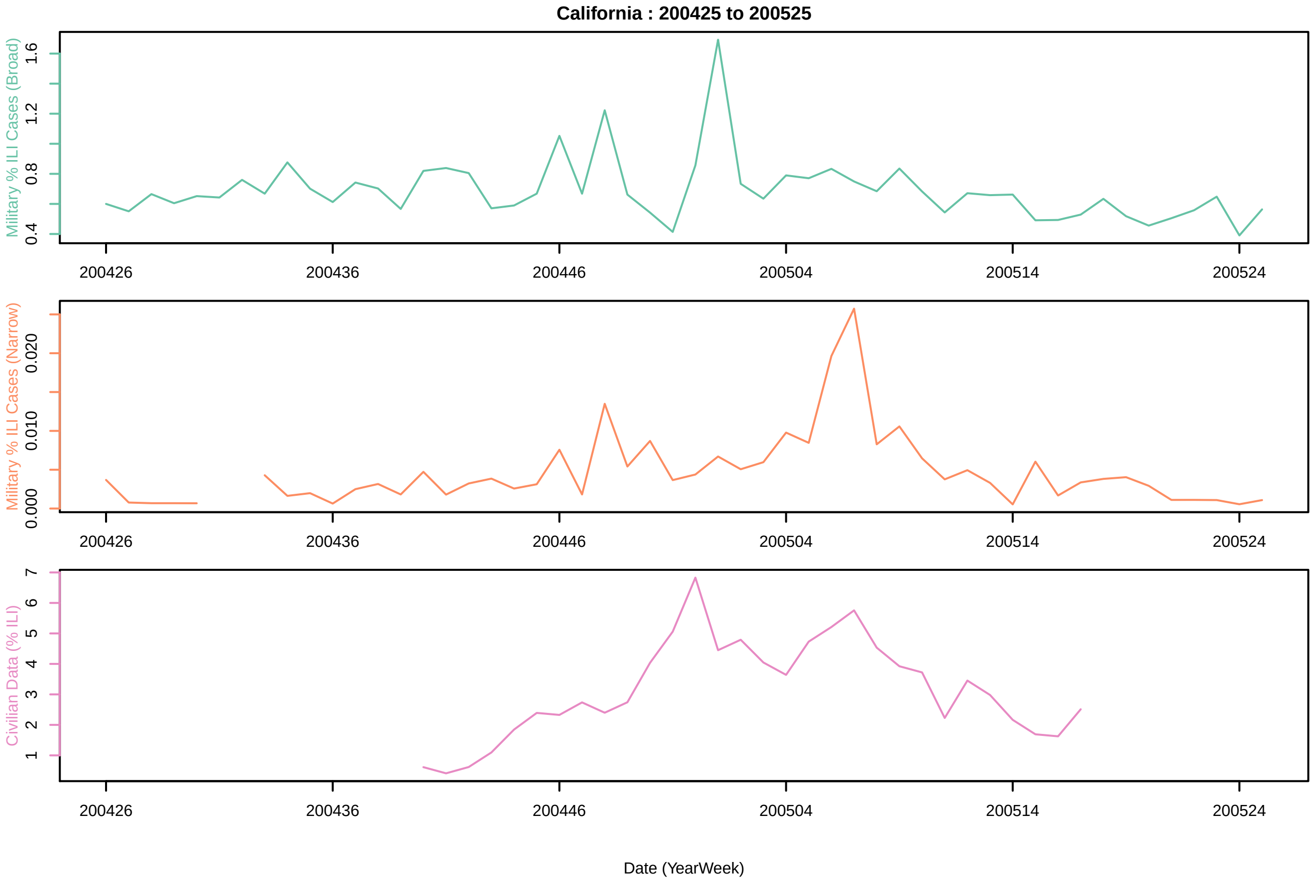

California : 200425 to 200525
Military % ILI Cases (Broad)
0.4
0.8
1.2
1.6
200426
200436
200446
200504
200514
200524
Military % ILI Cases (Narrow)
0.000
0.010
0.020
200426
200436
200446
200504
200514
200524
Civilian Data (% ILI)
1
2
3
4
5
6
7
200426
200436
200446
200504
200514
200524
Date (YearWeek)

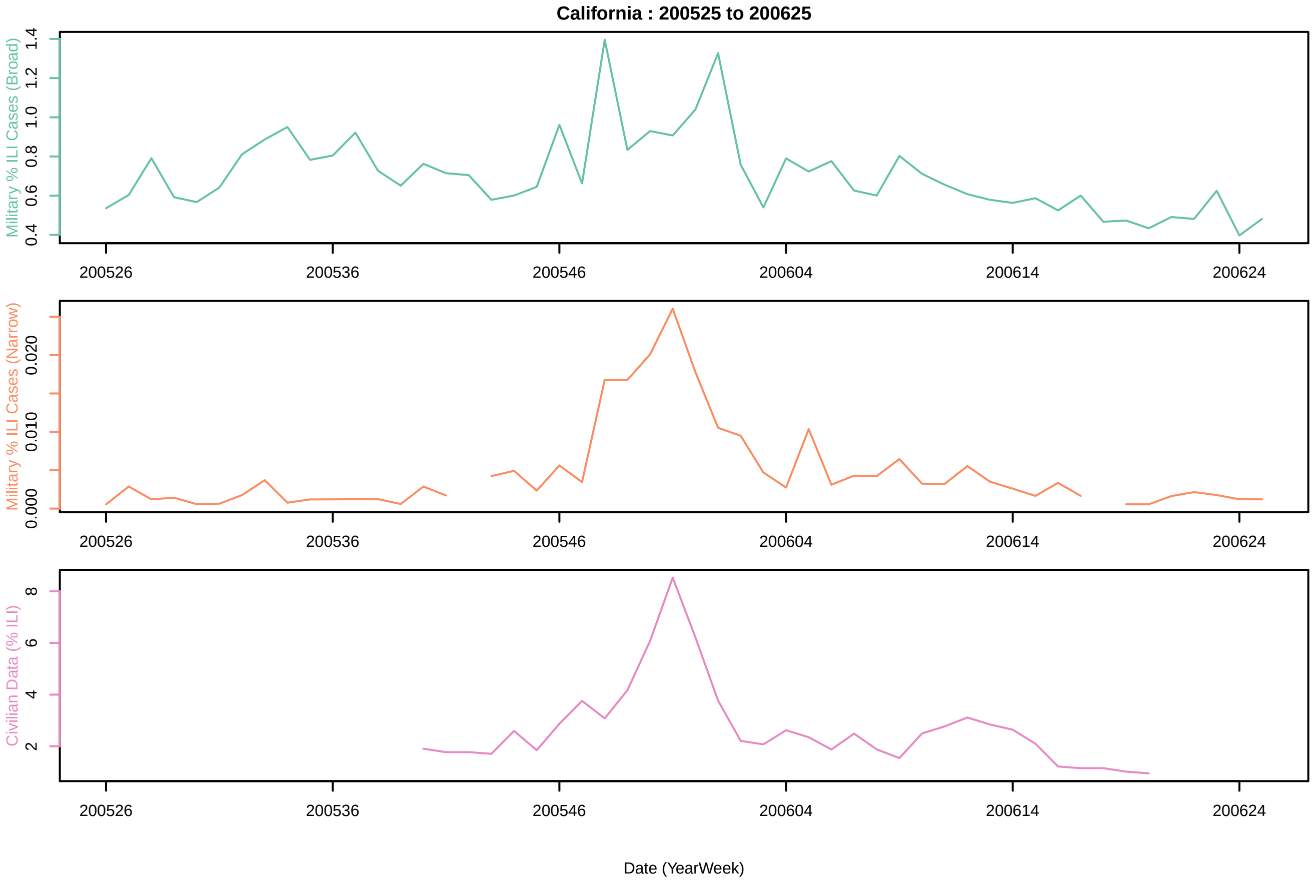
California : 200525 to 200625
Military % ILI Cases (Broad)
0.4
0.6
0.8
1.0
1.2
1.4
Military % ILI Cases (Narrow)
0.000
0.010
0.020
Civilian Data (% ILI)
2
4
6
8
200526
200536
200546
200604
200614
200624
Date (YearWeek)

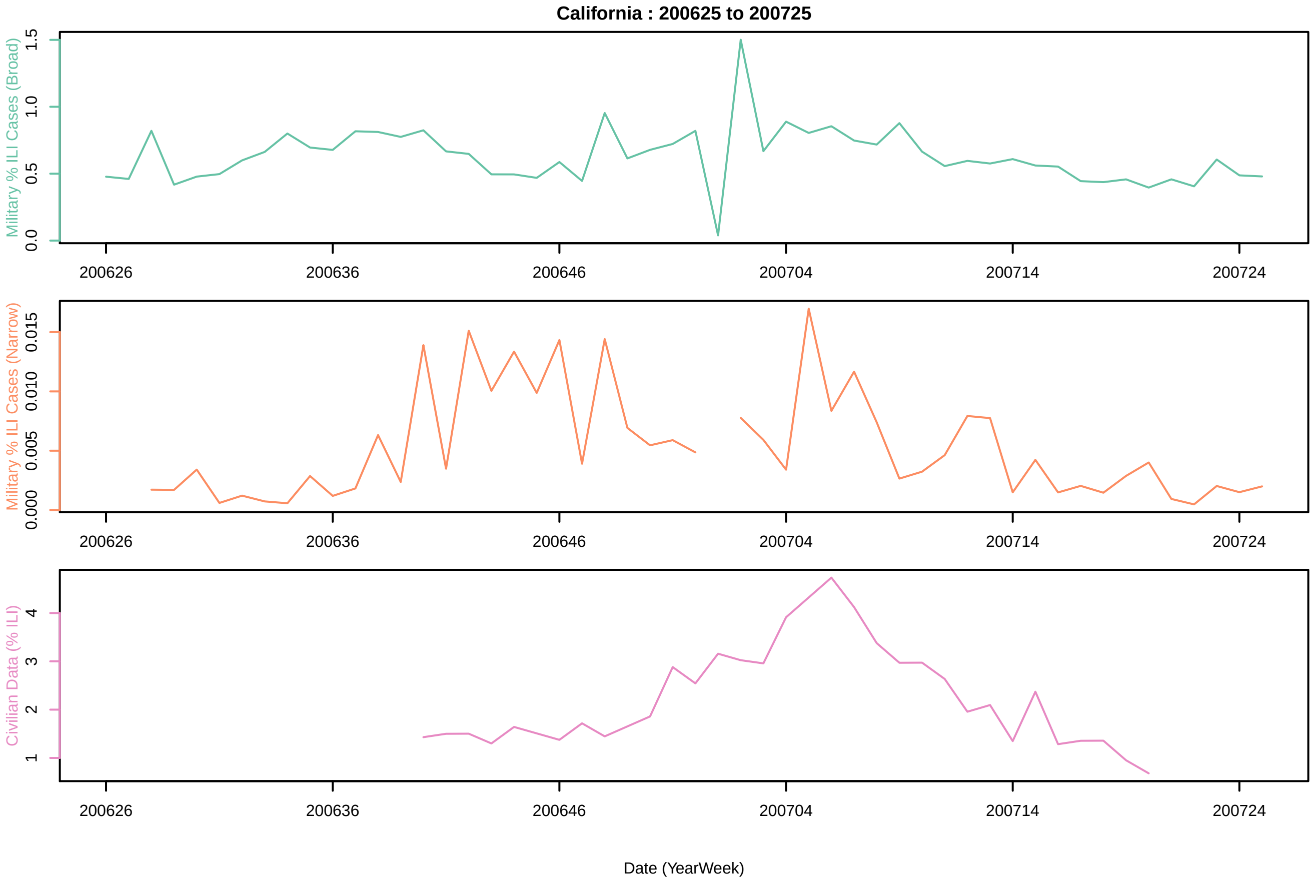
California : 200625 to 200725
Military % ILI Cases (Broad)
0.0
0.5
1.0
1.5
200626
200636
200646
200704
200714
200724
Military % ILI Cases (Narrow)
0.000
0.005
0.010
0.015
Civilian Data (% ILI)
1
2
3
4
Date (YearWeek)

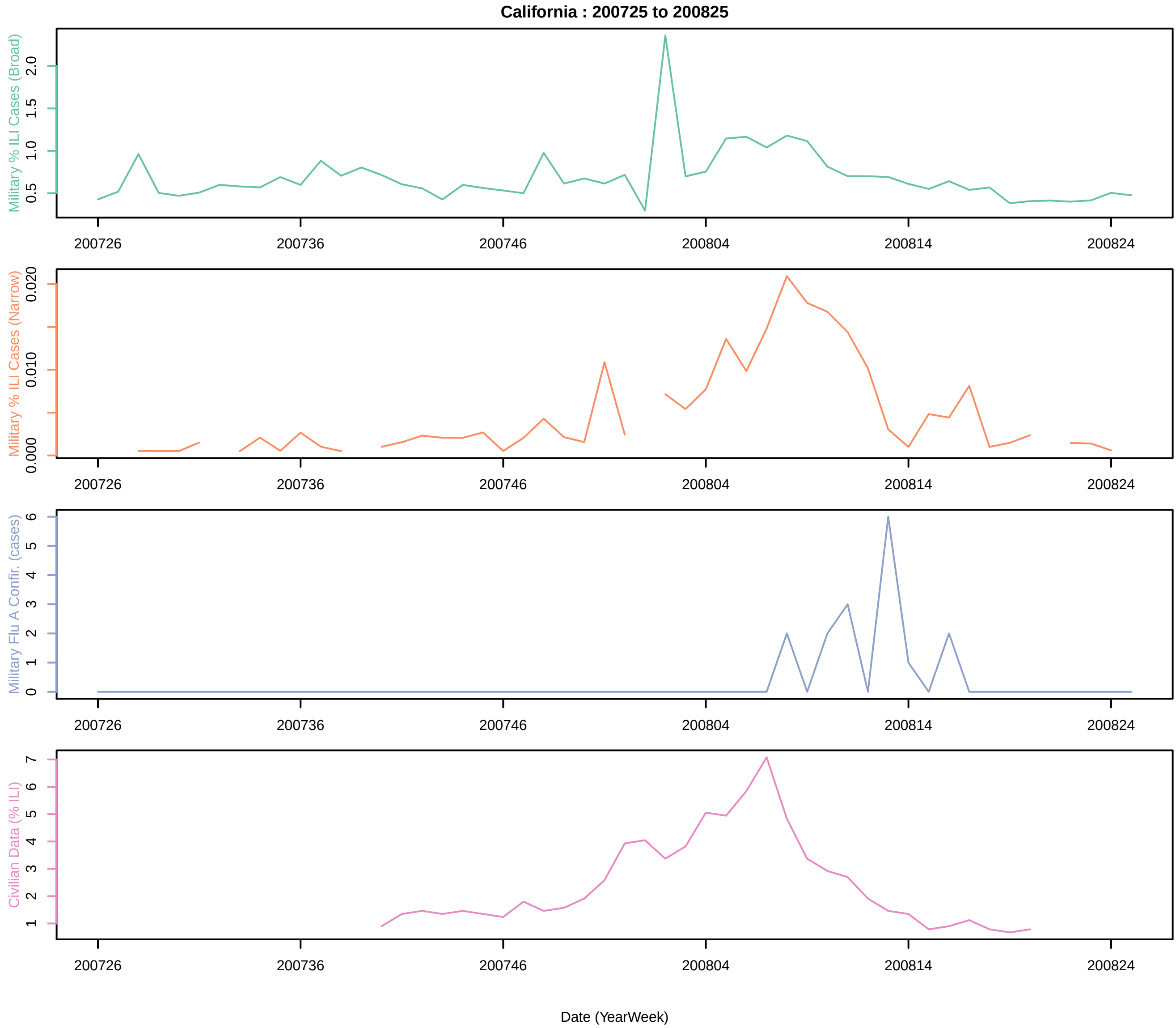
California : 200725 to 200825
Military % ILI Cases (Broad)
0.5
1.0
1.5
2.0
Military % ILI Cases (Narrow)
0.000
0.010
0.020
Military Flu A Confir. (cases)
0
1
2
3
4
5
6
Civilian Data (% ILI)
1
2
3
4
5
6
7
200726
200736
200746
200804
200814
200824
Date (YearWeek)

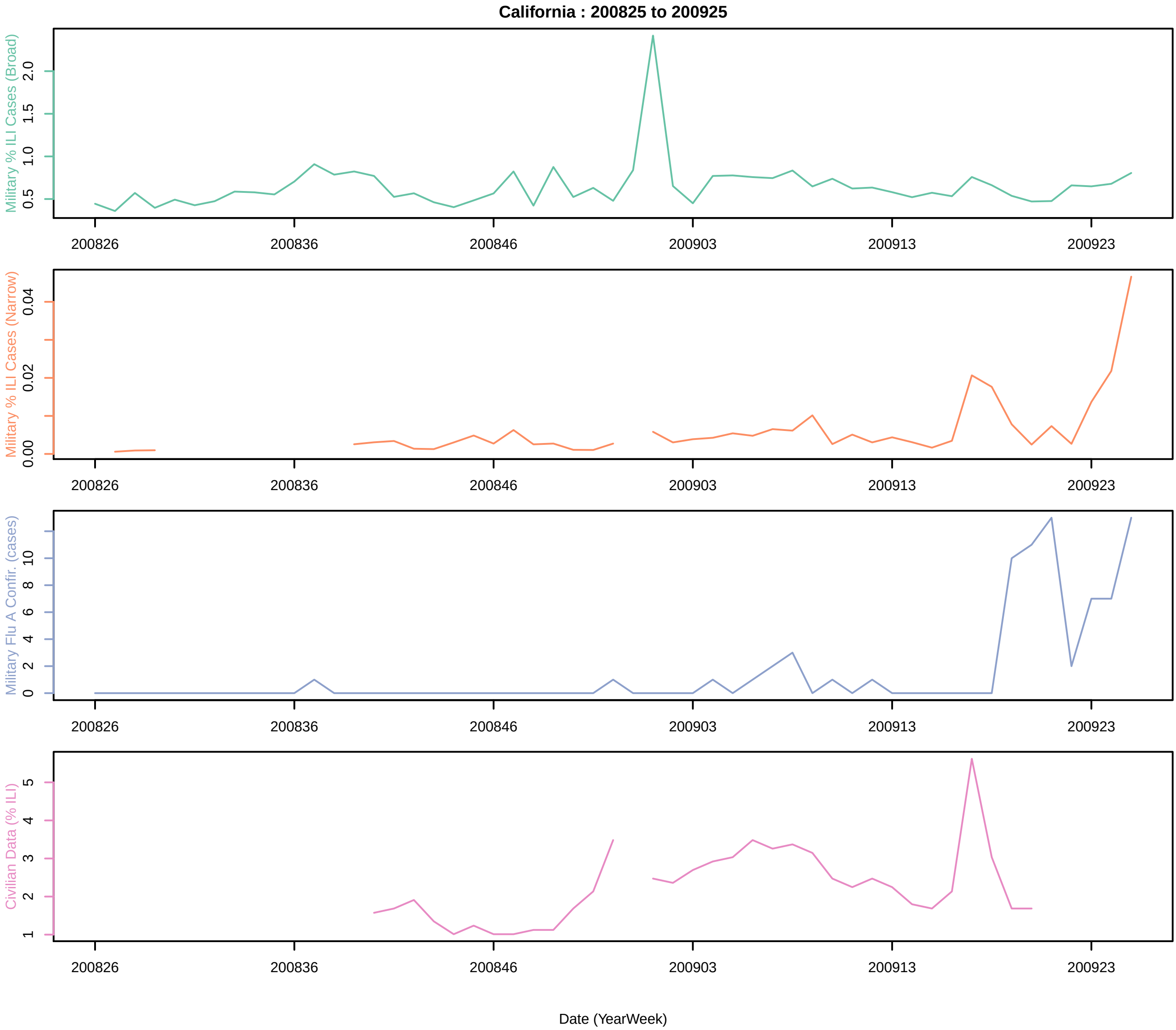

California : 200825 to 200925
Military % ILI Cases (Broad)
Military % ILI Cases (Narrow)
Military Flu A Confir. (cases)
Civilian Data (% ILI)
200826
200836
200846
200903
200913
200923
Date (YearWeek)

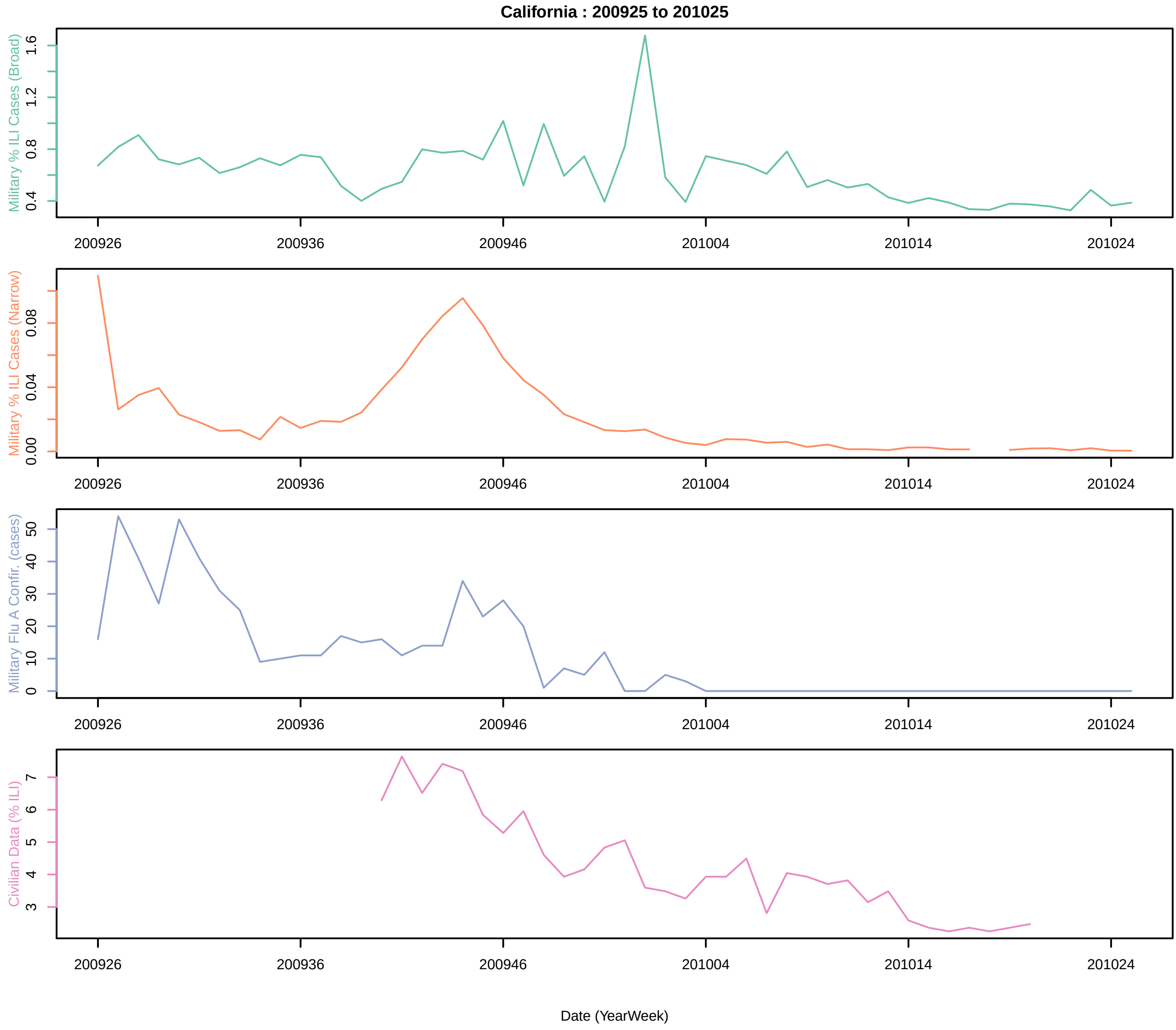
California : 200925 to 201025
Military % ILI Cases (Broad)
0.4
0.8
1.2
1.6
Military % ILI Cases (Narrow)
0.00
0.04
0.08
Military Flu A Confir. (cases)
0
10
20
30
40
50
Civilian Data (% ILI)
3
4
5
6
7
200926
200936
200946
201004
201014
201024
Date (YearWeek)

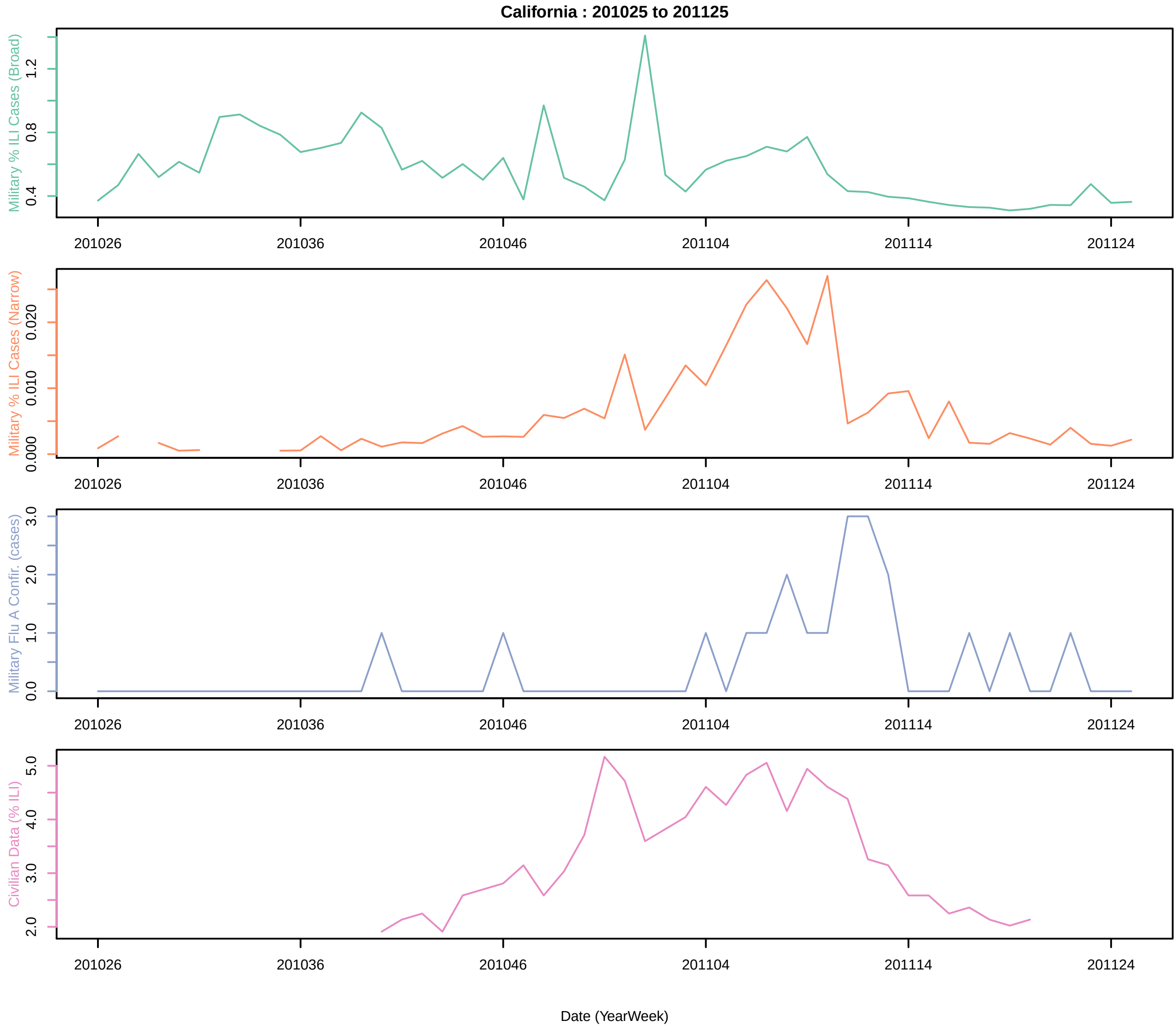
California : 201025 to 201125
Military % ILI Cases (Broad)
Military % ILI Cases (Narrow)
Military Flu A Confir. (cases)
Civilian Data (% ILI)
201026
201036
201046
201104
201114
201124
Date (YearWeek)

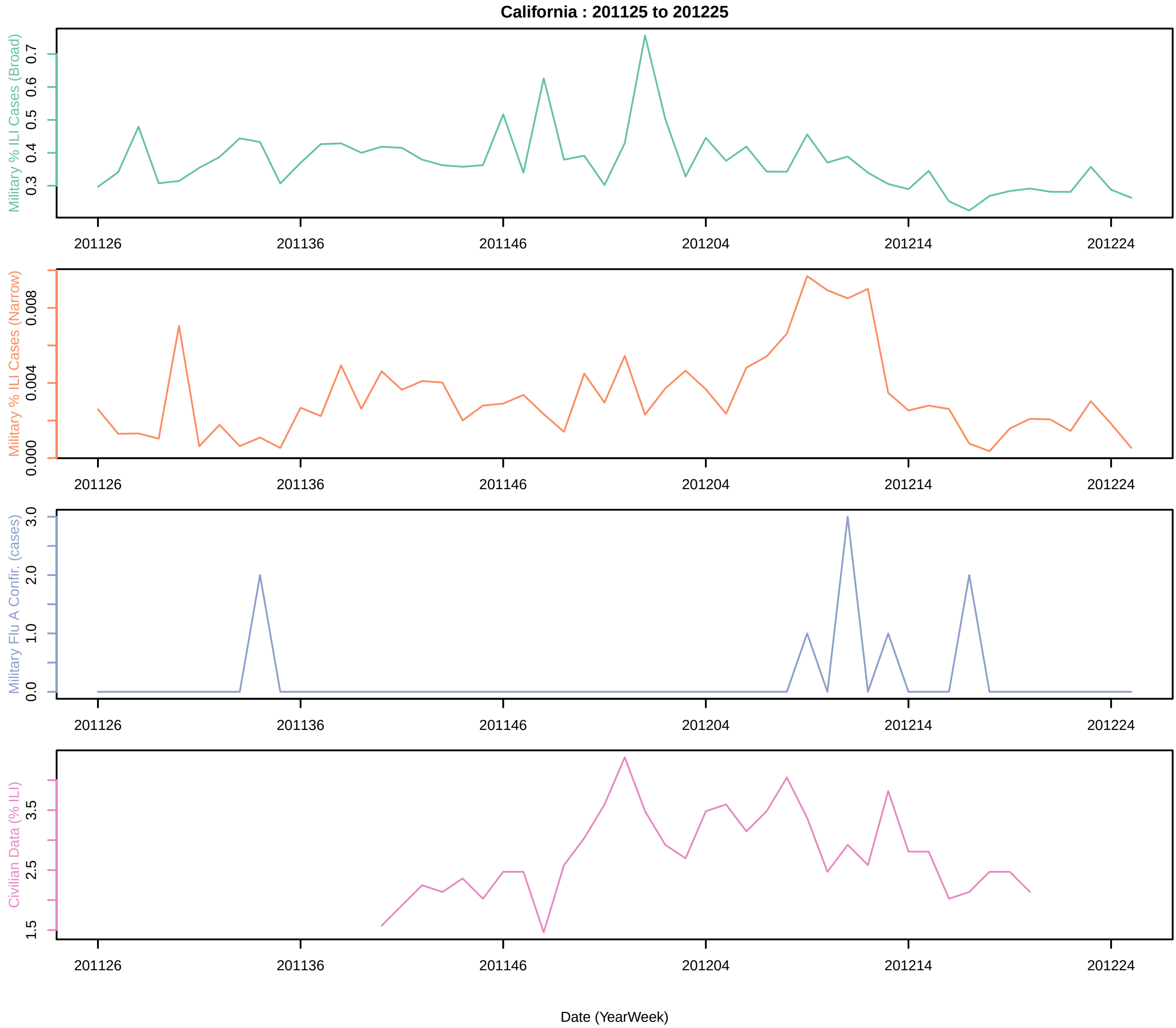

California : 201125 to 201225
Military % ILI Cases (Broad)
0.3
0.4
0.5
0.6
0.7
Military % ILI Cases (Narrow)
0.000
0.004
0.008
Military Flu A Confir. (cases)
0.0
1.0
2.0
3.0
Civilian Data (% ILI)
1.5
2.5
3.5
201126
201136
201146
201204
201214
201224
Date (YearWeek)

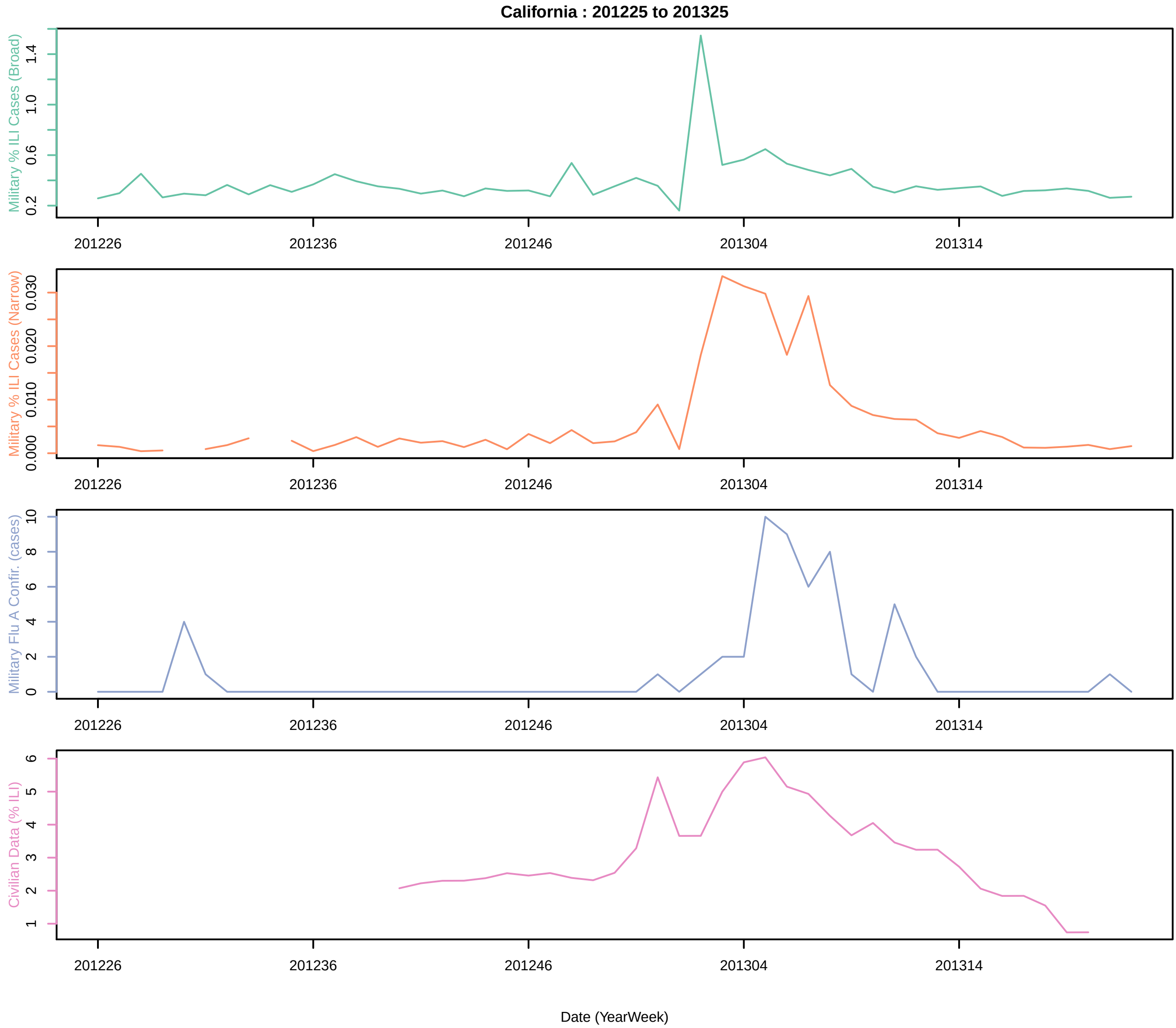

California : 201225 to 201325
Military % ILI Cases (Broad)
0.2
0.6
1.0
1.4
201226
201236
201246
201304
201314
Military % ILI Cases (Narrow)
0.000
0.010
0.020
0.030
201226
201236
201246
201304
201314
Military Flu A Confir. (cases)
0
2
4
6
8
10
201226
201236
201246
201304
201314
Civilian Data (% ILI)
1
2
3
4
5
6
201226
201236
201246
201304
201314
Date (YearWeek)

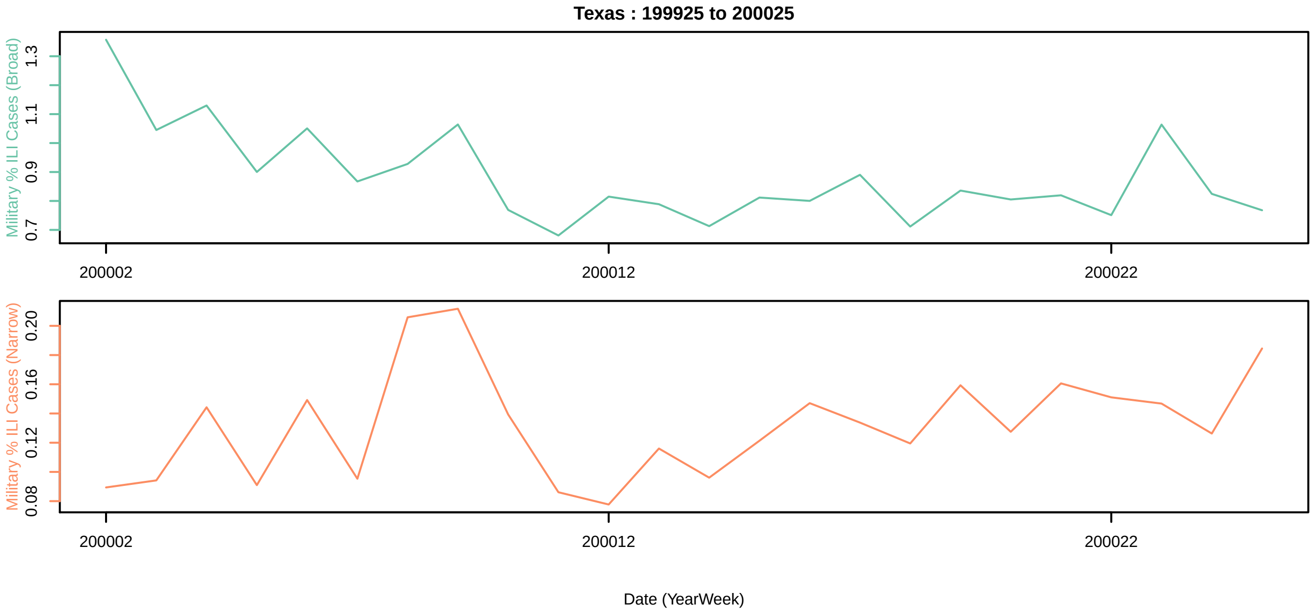

Texas : 199925 to 200025
Military % ILI Cases (Broad)
0.7
0.9
1.1
1.3
200002
200012
200022
Military % ILI Cases (Narrow)
0.08
0.12
0.16
0.20
200002
200012
200022
Date (YearWeek)

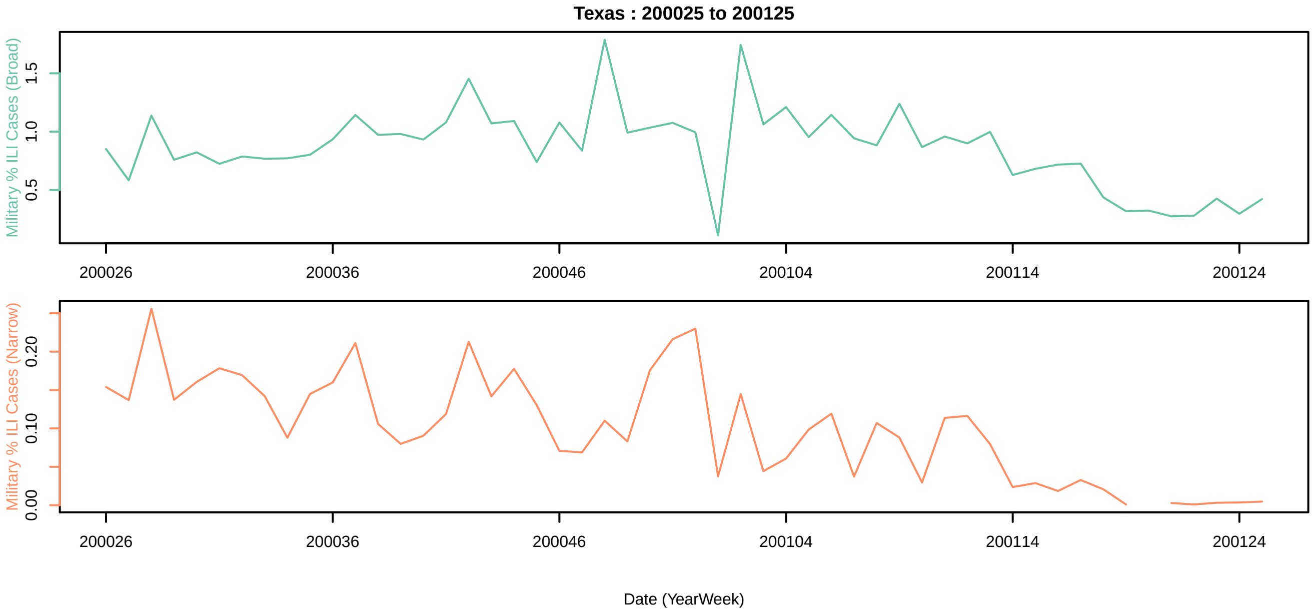
Texas : 200025 to 200125
Military % ILI Cases (Broad)
0.5
1.0
1.5
200026
200036
200046
200104
200114
200124
Military % ILI Cases (Narrow)
0.00
0.10
0.20
200026
200036
200046
200104
200114
200124
Date (YearWeek)

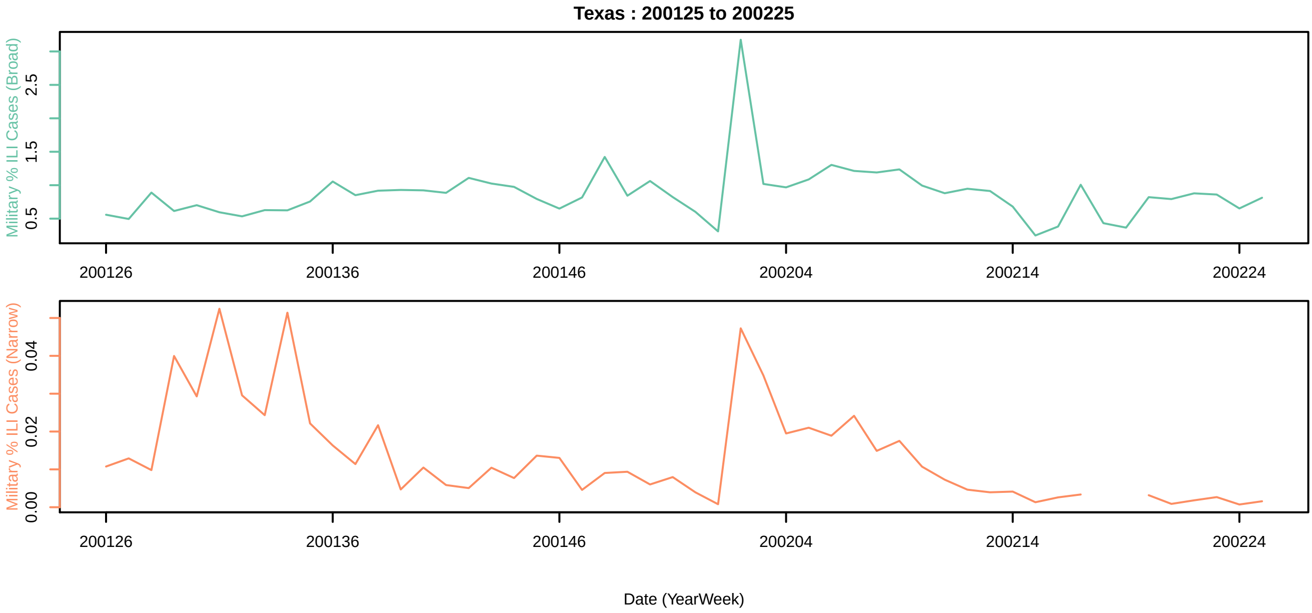
Texas : 200125 to 200225
Military % ILI Cases (Broad)
0.5
1.5
2.5
200126
200136
200146
200204
200214
200224
Military % ILI Cases (Narrow)
0.00
0.02
0.04
Date (YearWeek)

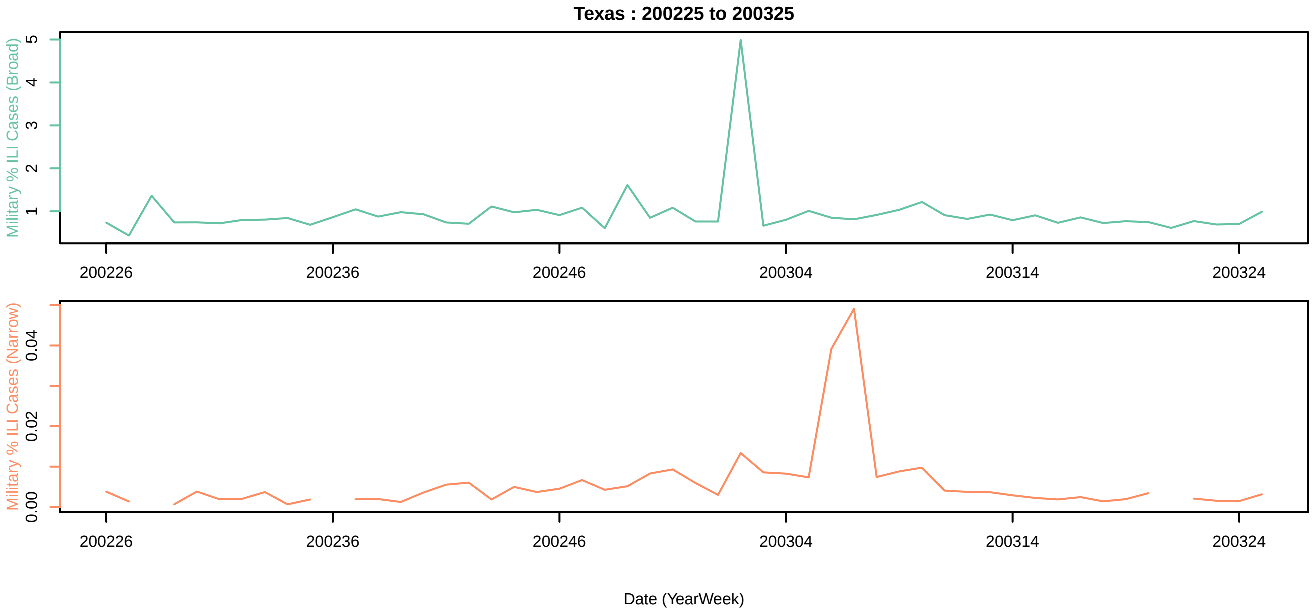
Texas : 200225 to 200325
Military % ILI Cases (Broad)
1
2
3
4
5
200226
200236
200246
200304
200314
200324
Military % ILI Cases (Narrow)
0.00
0.02
0.04
200226
200236
200246
200304
200314
200324
Date (YearWeek)

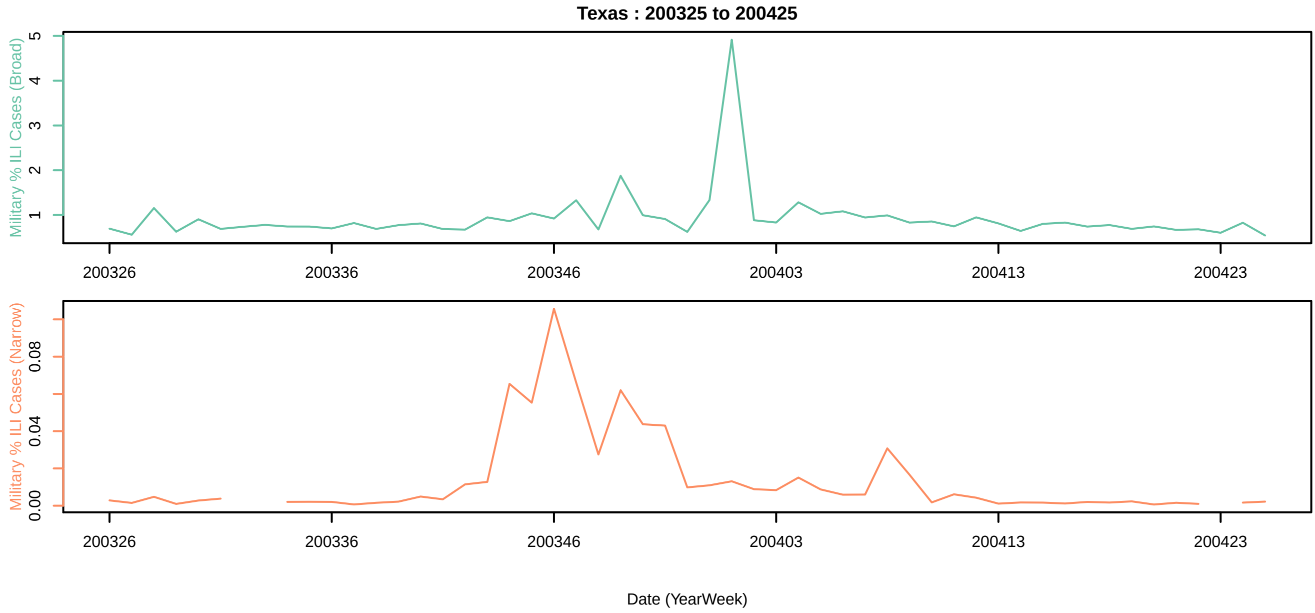

Texas : 200325 to 200425
Military % ILI Cases (Broad)
1
2
3
4
5
200326
200336
200346
200403
200413
200423
Military % ILI Cases (Narrow)
0.00
0.04
0.08
200326
200336
200346
200403
200413
200423
Date (YearWeek)

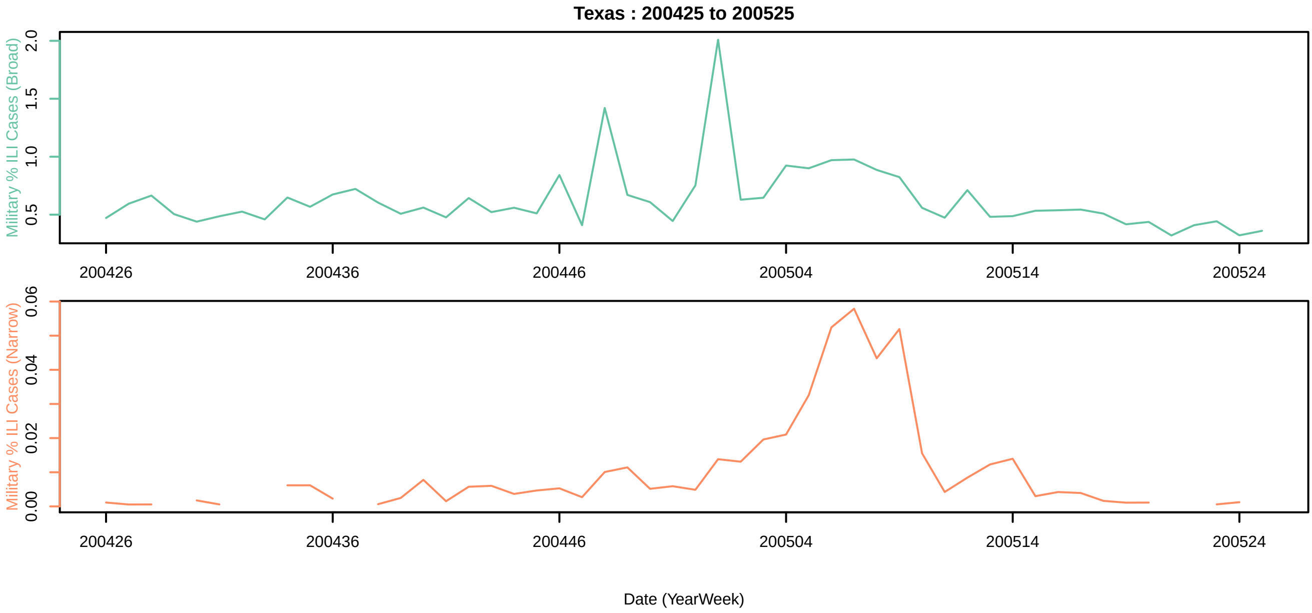

Texas : 200425 to 200525
Military % ILI Cases (Broad)
0.5
1.0
1.5
2.0
200426
200436
200446
200504
200514
200524
Military % ILI Cases (Narrow)
0.00
0.02
0.04
0.06
200426
200436
200446
200504
200514
200524
Date (YearWeek)

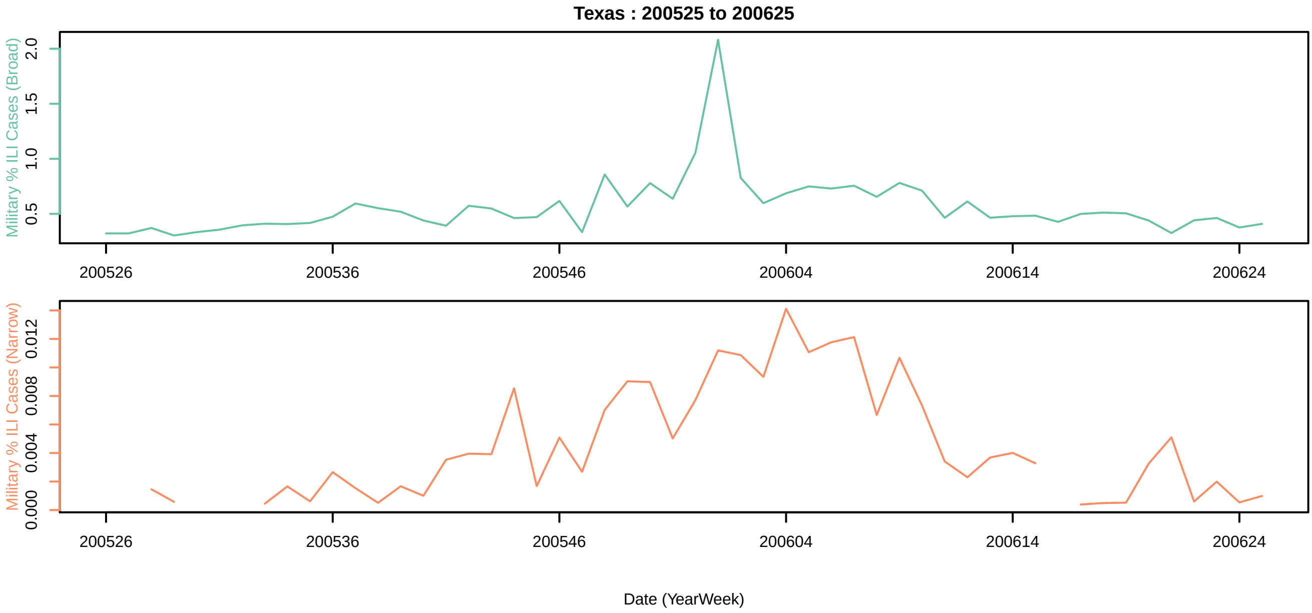
Texas : 200525 to 200625
Military % ILI Cases (Broad)
0.5
1.0
1.5
2.0
200526
200536
200546
200604
200614
200624
Military % ILI Cases (Narrow)
0.000
0.004
0.008
0.012
200526
200536
200546
200604
200614
200624
Date (YearWeek)

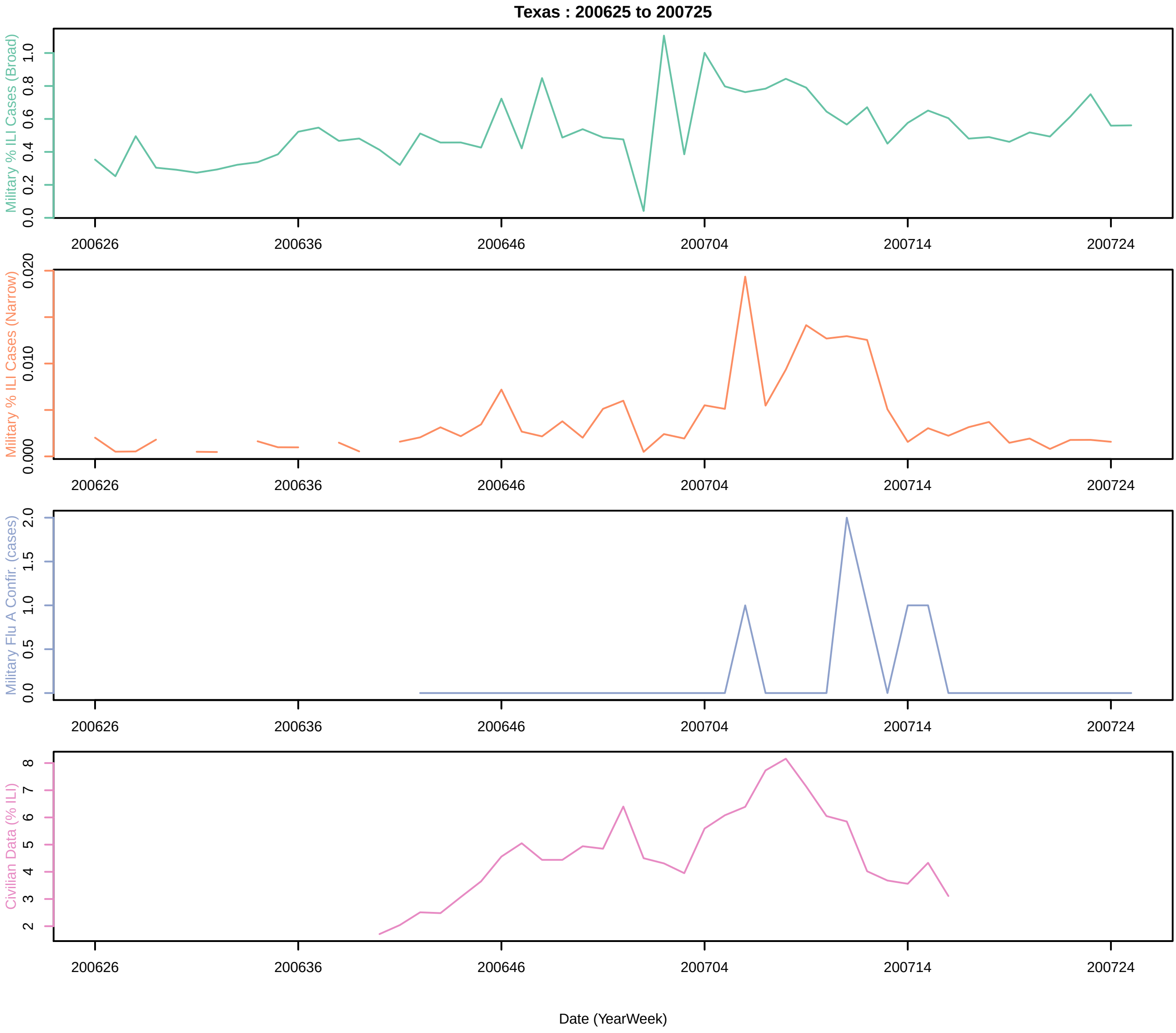

Texas : 200625 to 200725
Military % ILI Cases (Broad)
0.0
0.2
0.4
0.6
0.8
1.0
200626
200636
200646
200704
200714
200724
Military % ILI Cases (Narrow)
0.000
0.010
0.020
Military Flu A Confir. (cases)
0.0
0.5
1.0
1.5
2.0
Civilian Data (% ILI)
2
3
4
5
6
7
8
Date (YearWeek)

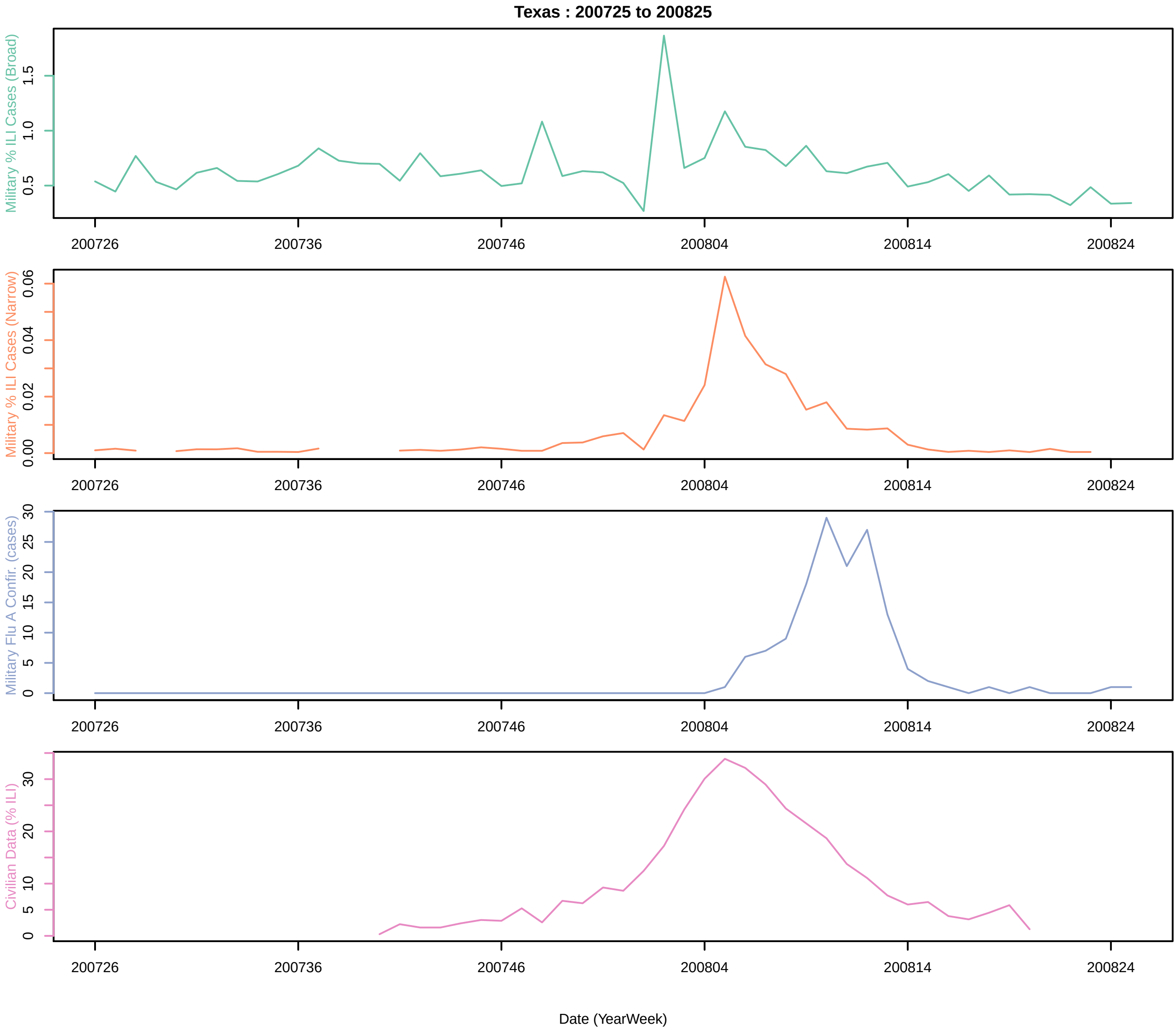

Texas : 200725 to 200825
Military % ILI Cases (Broad)
0.5
1.0
1.5
200726
200736
200746
200804
200814
200824
Military % ILI Cases (Narrow)
0.00
0.02
0.04
0.06
Military Flu A Confir. (cases)
0
5
10
15
20
25
30
Civilian Data (% ILI)
0
5
10
20
30
Date (YearWeek)

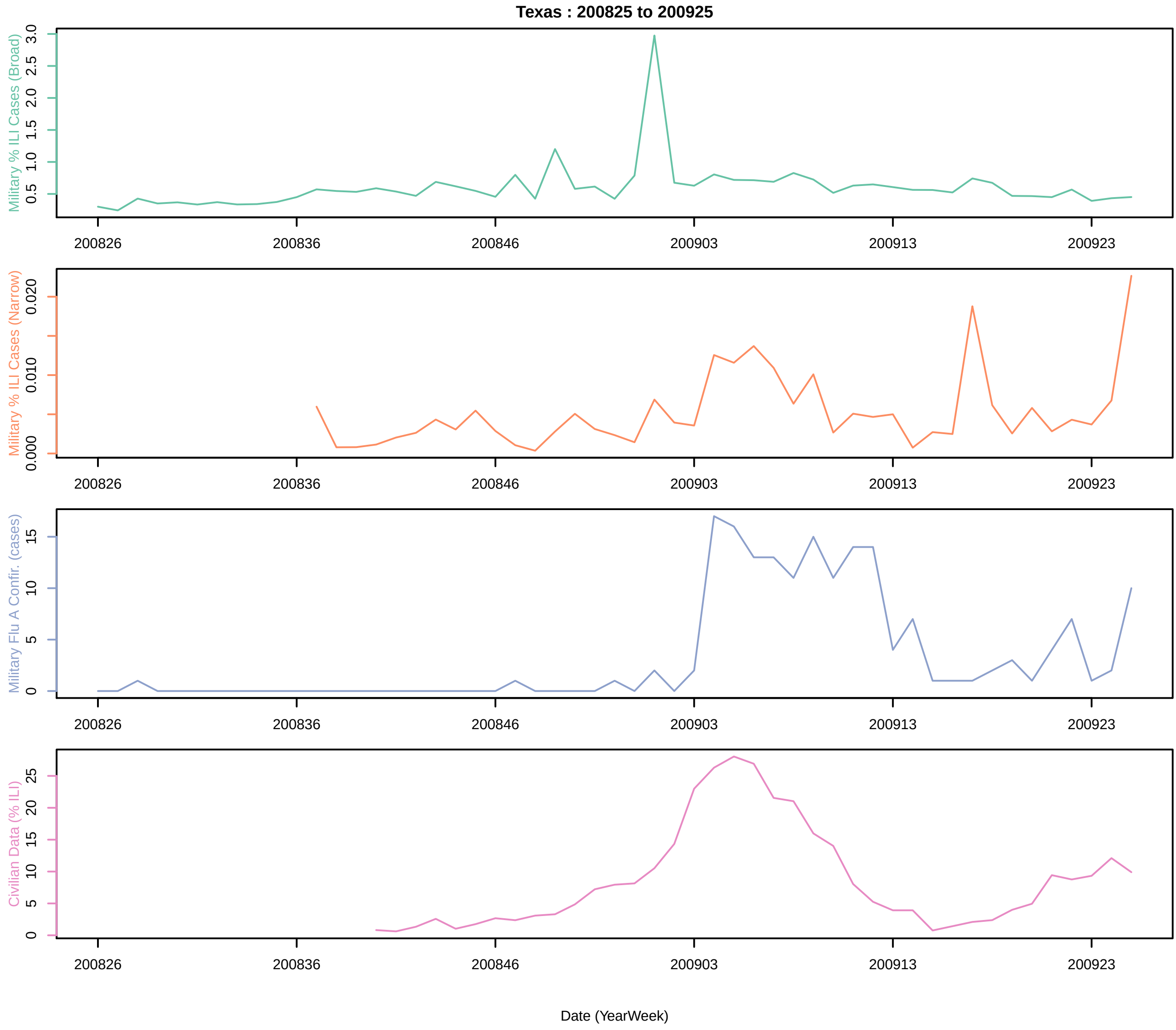
Texas : 200825 to 200925
Military % ILI Cases (Broad)
Military % ILI Cases (Narrow)
Military Flu A Confir. (cases)
Civilian Data (% ILI)
200826
200836
200846
200903
200913
200923
Date (YearWeek)

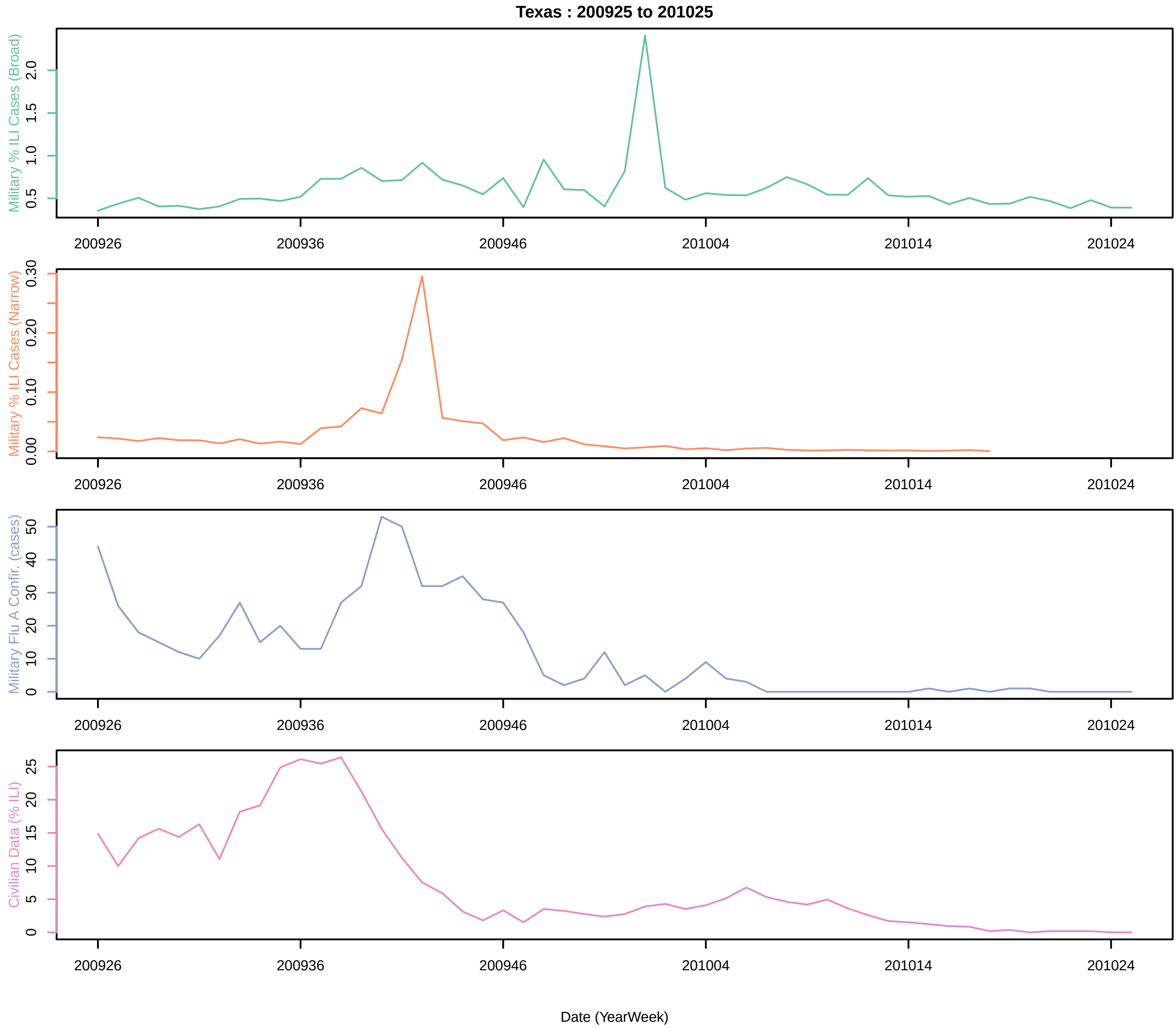

Texas : 200925 to 201025
Military % ILI Cases (Broad)
0.5
1.0
1.5
2.0
Military % ILI Cases (Narrow)
0.00
0.10
0.20
0.30
Military Flu A Confir. (cases)
0
10
20
30
40
50
Civilian Data (% ILI)
0
5
10
15
20
25
200926
200936
200946
201004
201014
201024
Date (YearWeek)

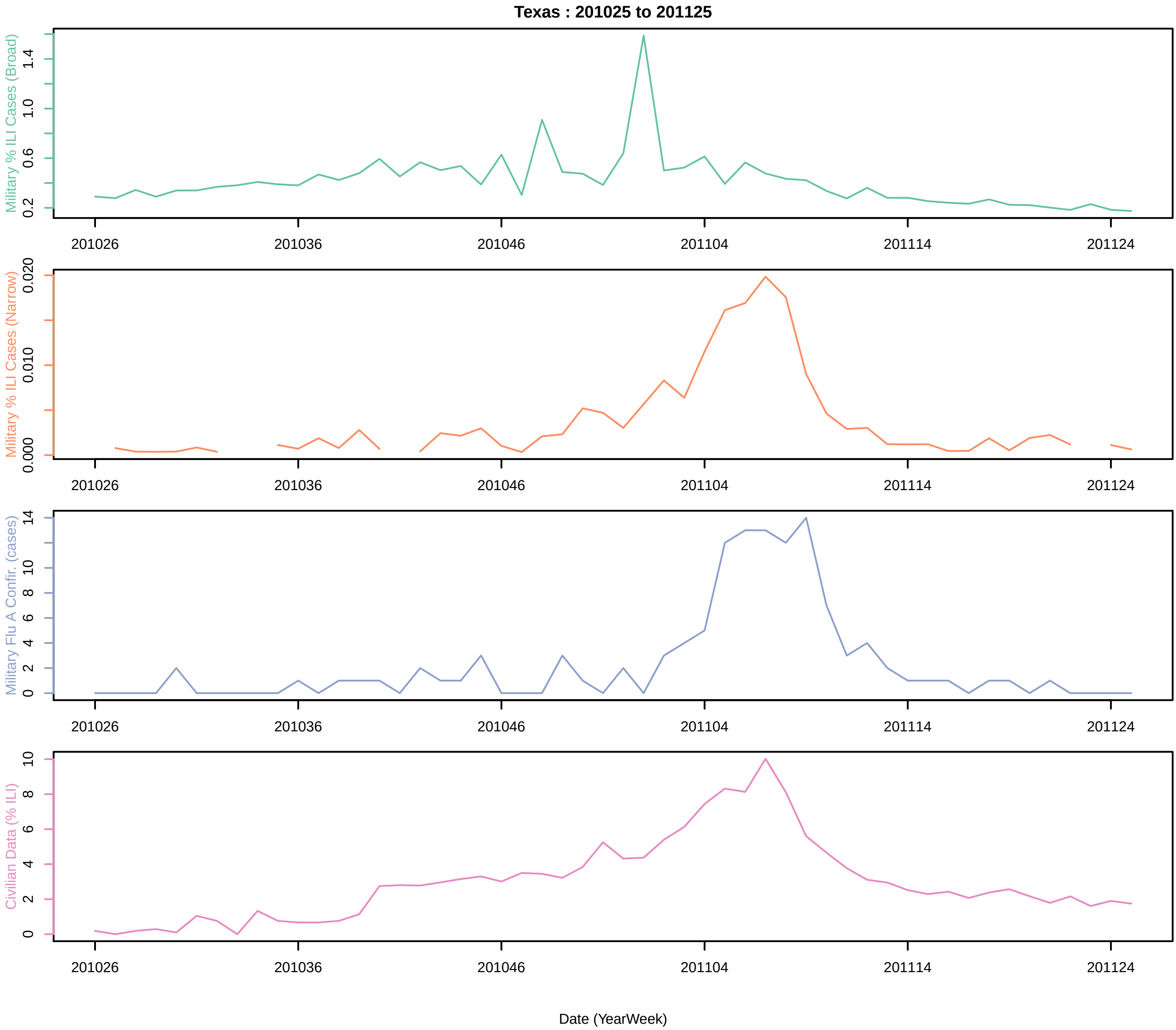

Texas : 201025 to 201125
Military % ILI Cases (Broad)
0.2
0.6
1.0
1.4
Military % ILI Cases (Narrow)
0.000
0.010
0.020
Military Flu A Confir. (cases)
0
2
4
6
8
10
14
Civilian Data (% ILI)
0
2
4
6
8
10
201026
201036
201046
201104
201114
201124
Date (YearWeek)

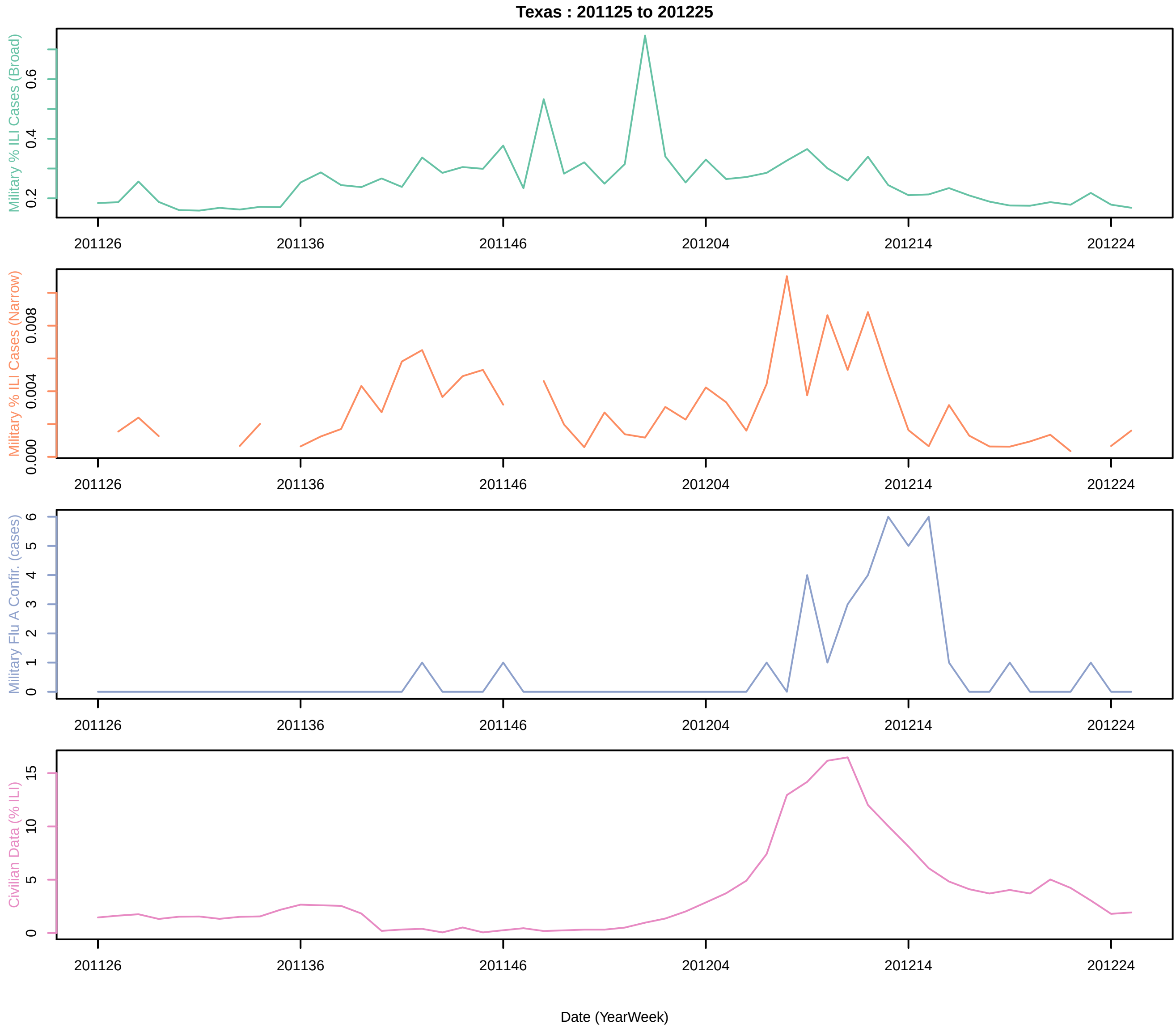
Texas : 201125 to 201225
Military % ILI Cases (Broad)
0.2
0.4
0.6
Military % ILI Cases (Narrow)
0.000
0.004
0.008
Military Flu A Confir. (cases)
0
1
2
3
4
5
6
Civilian Data (% ILI)
0
5
10
15
201126
201136
201146
201204
201214
201224
Date (YearWeek)

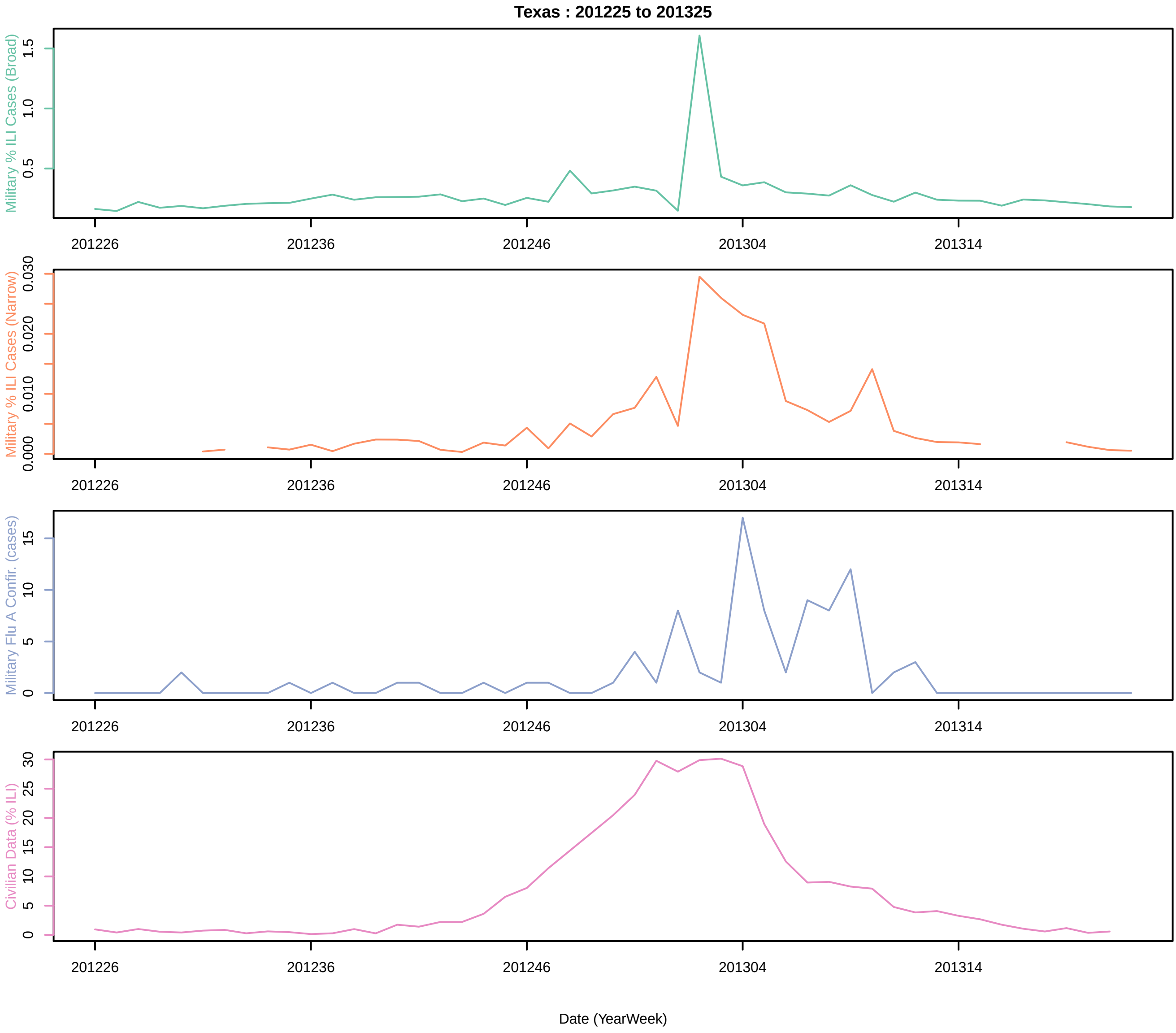
Texas : 201225 to 201325
Military % ILI Cases (Broad)
0.5
1.0
1.5
Military % ILI Cases (Narrow)
0.000
0.010
0.020
0.030
Military Flu A Confir. (cases)
0
5
10
15
Civilian Data (% ILI)
0
5
10
15
20
25
30
201226
201236
201246
201304
201314
Date (YearWeek)

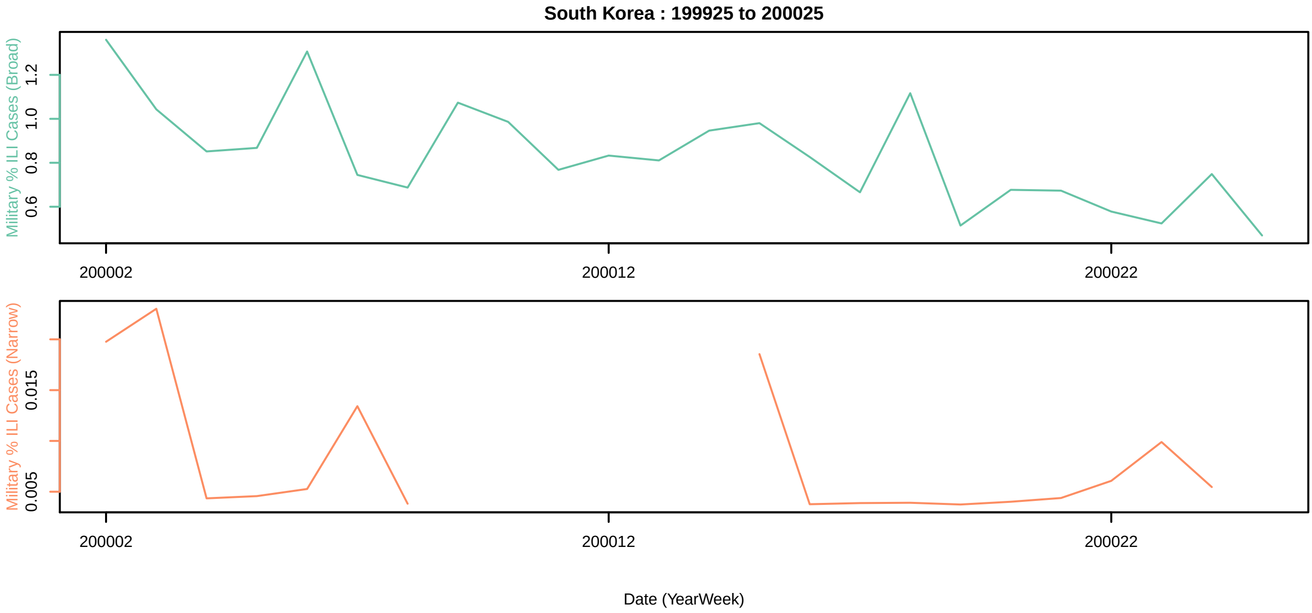
South Korea : 199925 to 200025
Military % ILI Cases (Broad)
0.6
0.8
1.0
1.2
200002
200012
200022
Military % ILI Cases (Narrow)
0.005
0.015
200002
200012
200022
Date (YearWeek)

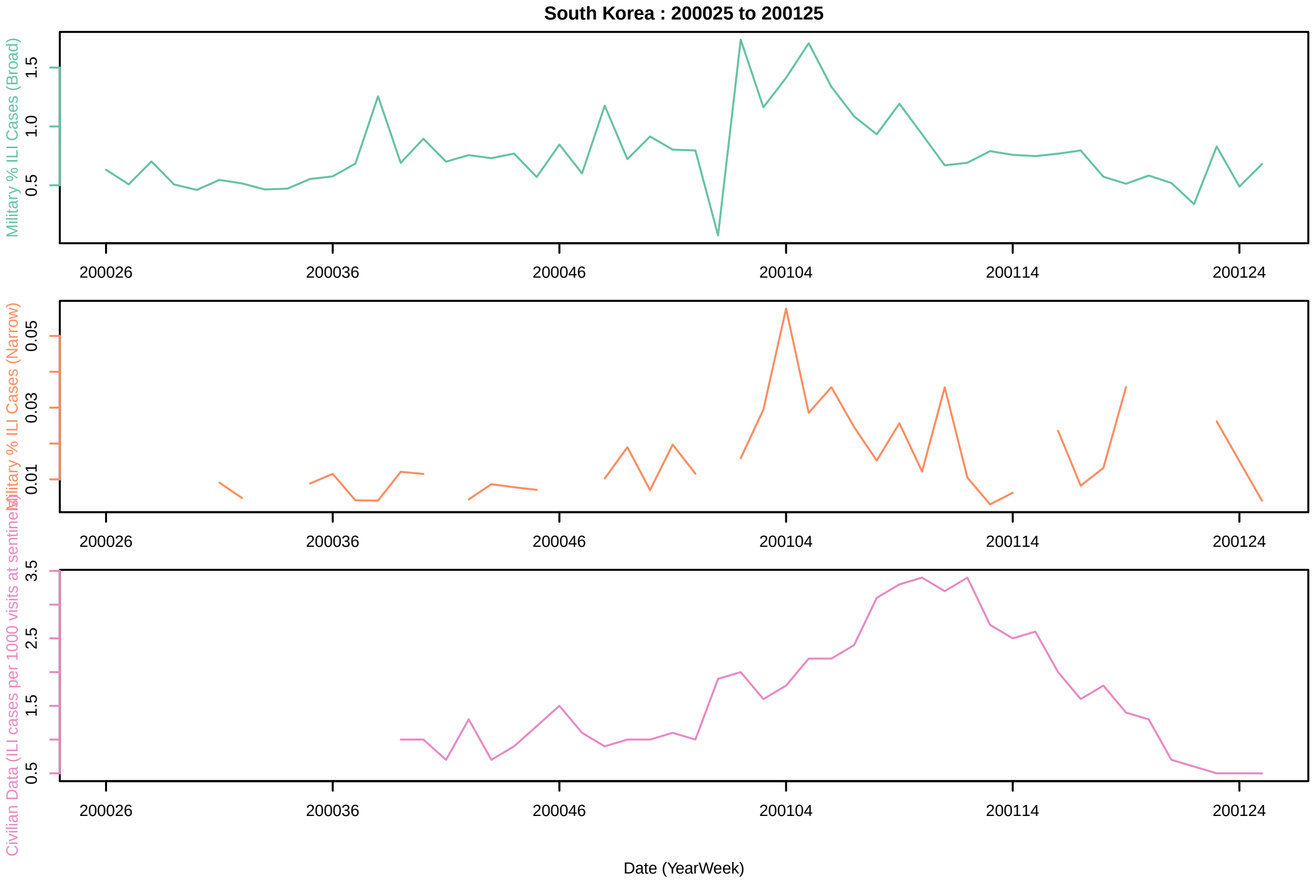

South Korea : 200025 to 200125
Military % ILI Cases (Broad)
0.5
1.0
1.5
Military % ILI Cases (Narrow)
0.01
0.03
0.05
Civilian Data (ILI cases per 1000 visits at sentinels)
0.5
1.5
2.5
3.5
200026
200036
200046
200104
200114
200124
Date (YearWeek)

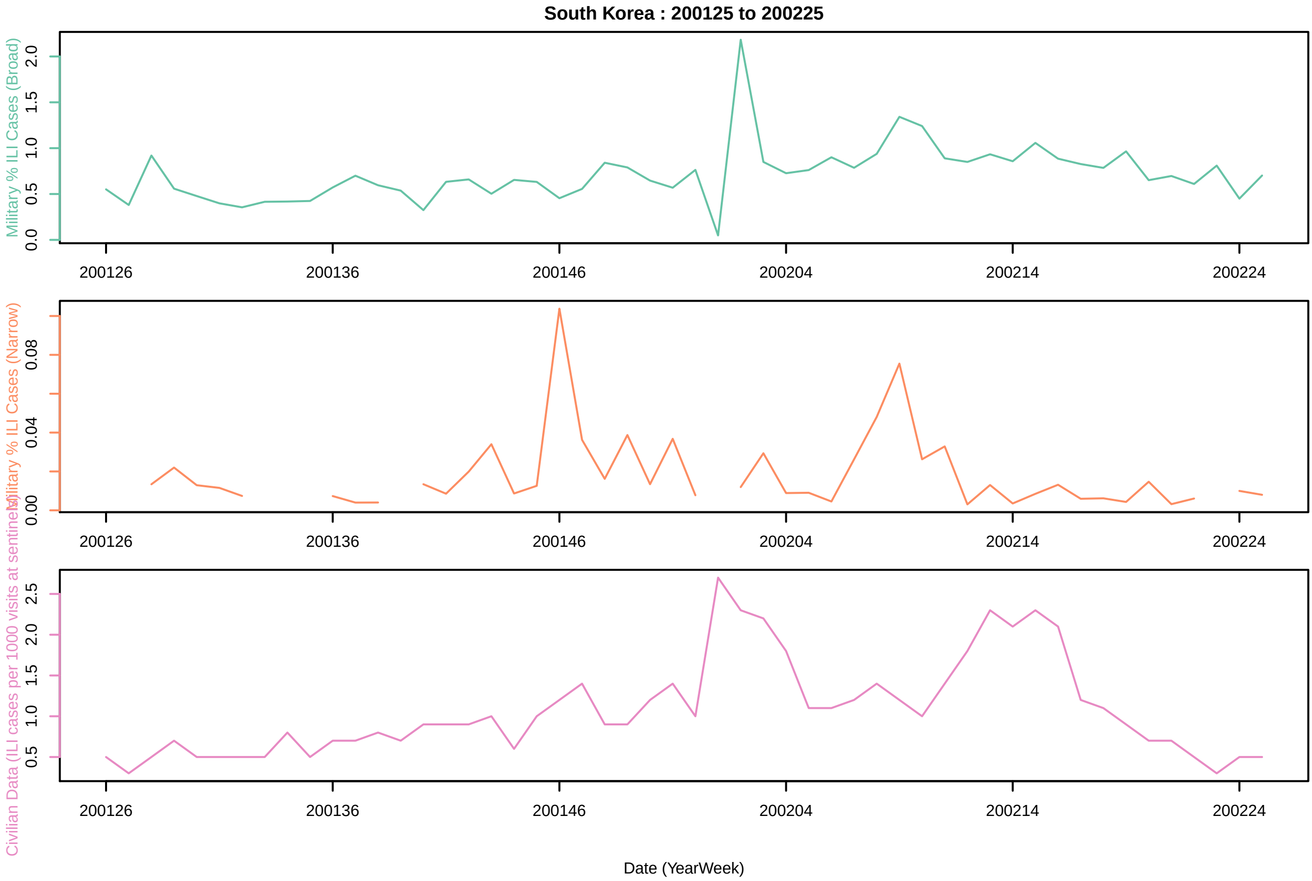

South Korea : 200125 to 200225
Military % ILI Cases (Broad)
0.0
0.5
1.0
1.5
2.0
200126
200136
200146
200204
200214
200224
Military % ILI Cases (Narrow)
0.00
0.04
0.08
Civilian Data (ILI cases per 1000 visits at sentinels)
0.5
1.0
1.5
2.0
2.5
Date (YearWeek)

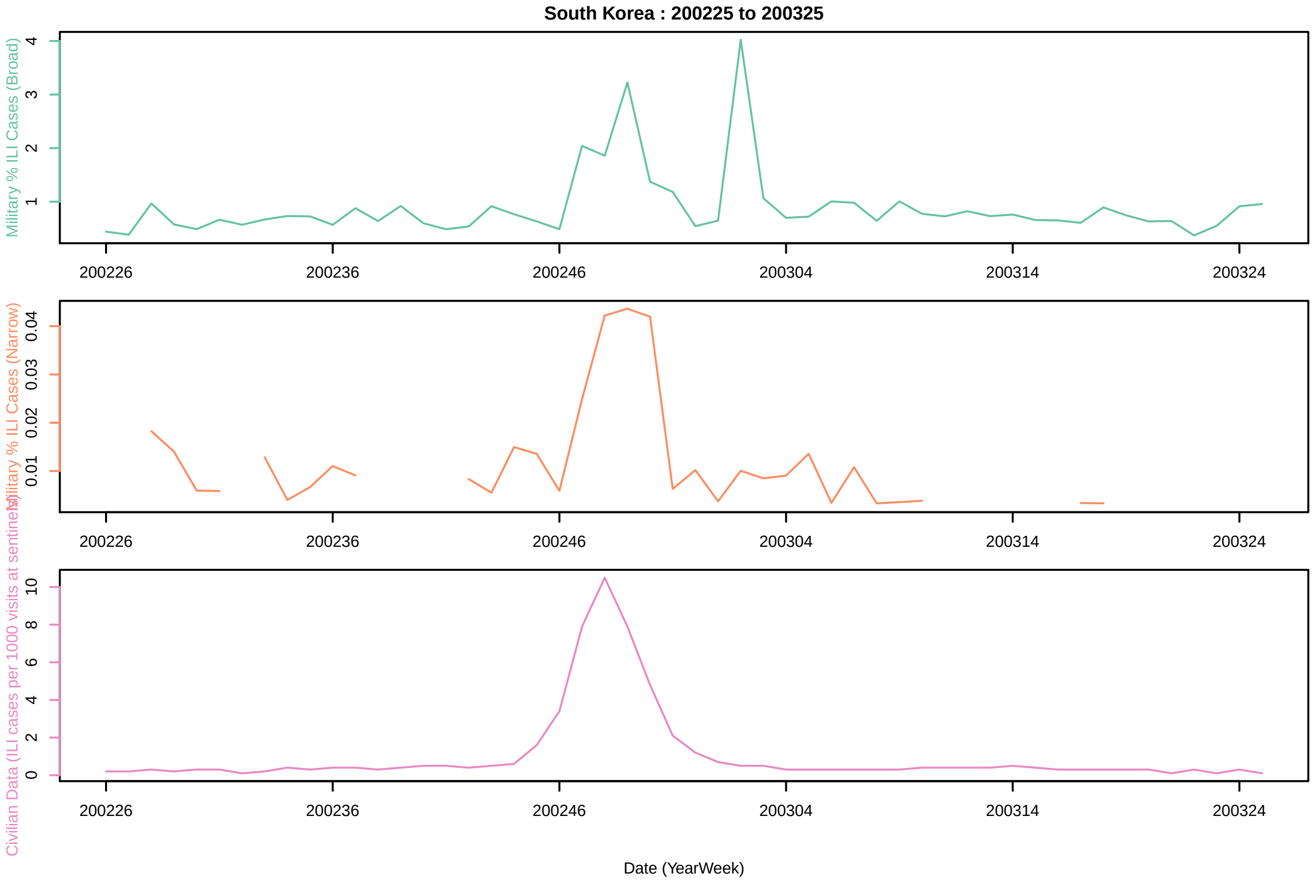
South Korea : 200225 to 200325
Military % ILI Cases (Broad)
Military % ILI Cases (Narrow)
Civilian Data (ILI cases per 1000 visits at sentinels)
200226
200236
200246
200304
200314
200324
Date (YearWeek)

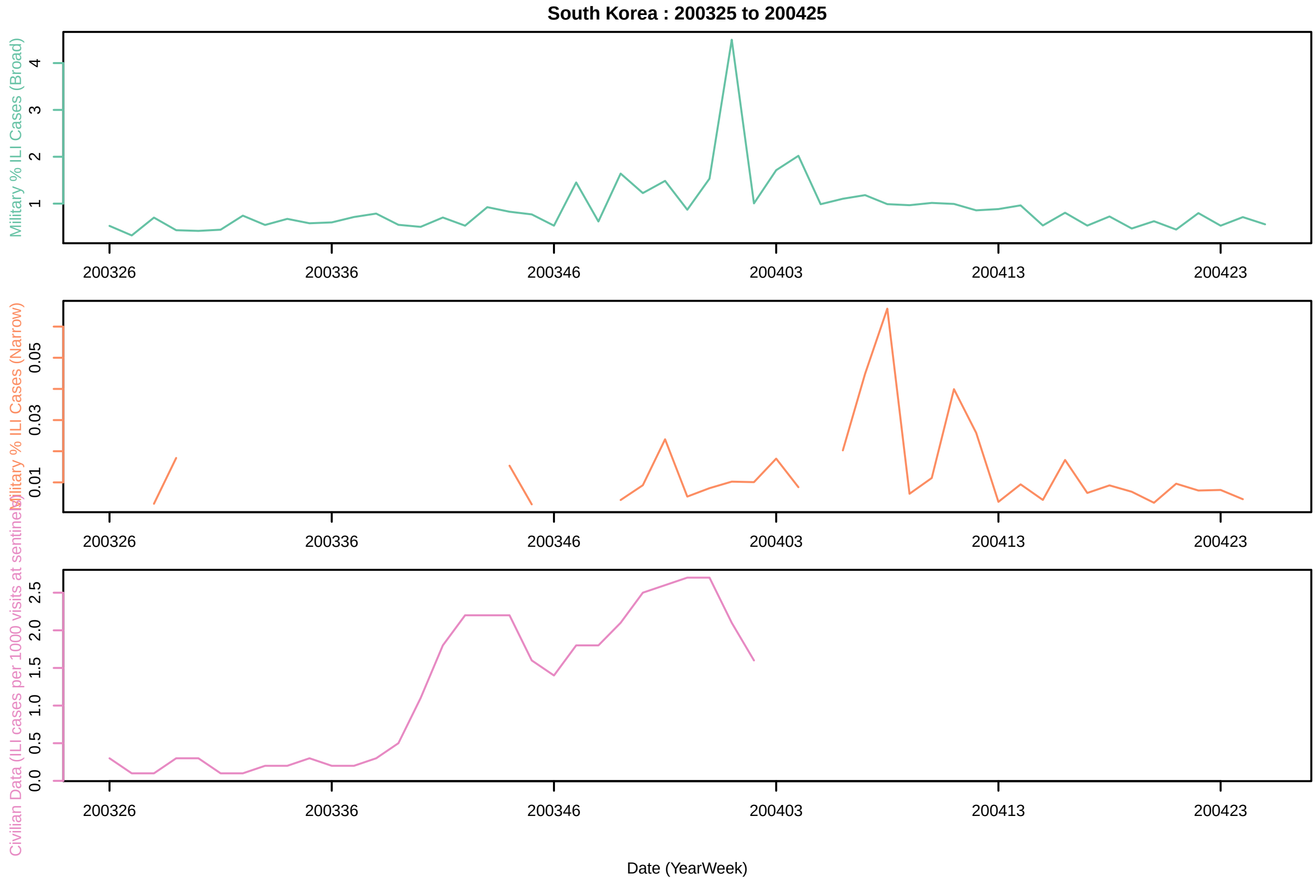
South Korea : 200325 to 200425
Military % ILI Cases (Broad)
Military % ILI Cases (Narrow)
Civilian Data (ILI cases per 1000 visits at sentinels)
200326
200336
200346
200403
200413
200423
Date (YearWeek)

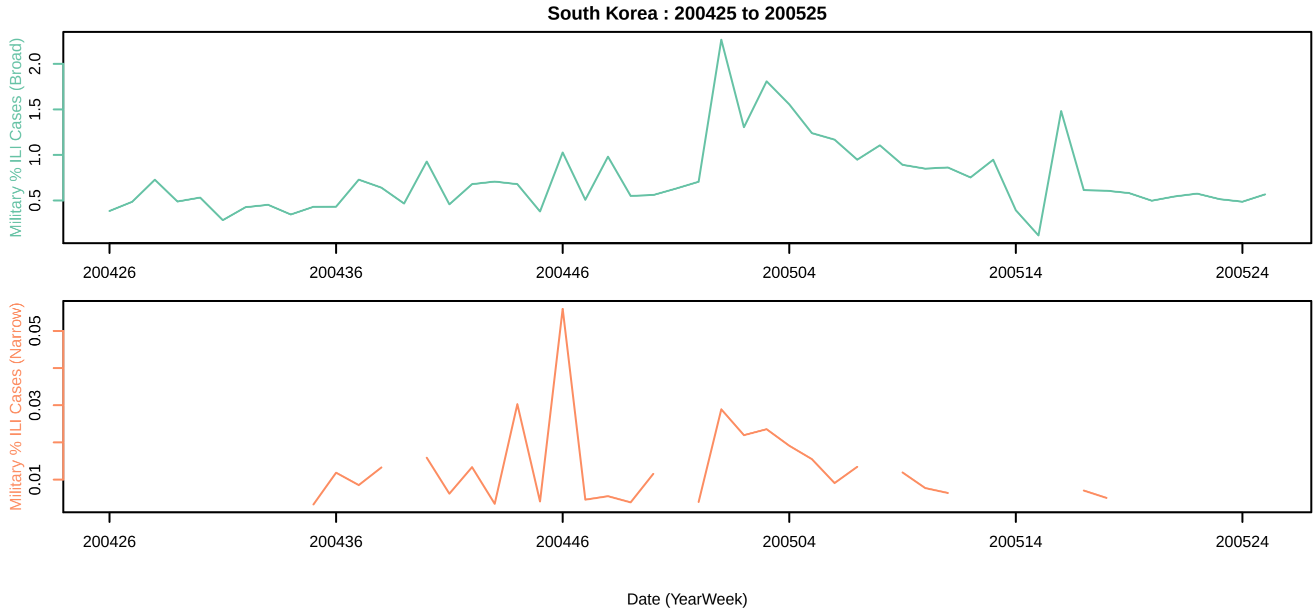

South Korea : 200425 to 200525
Military % ILI Cases (Broad)
0.5
1.0
1.5
2.0
200426
200436
200446
200504
200514
200524
Military % ILI Cases (Narrow)
0.01
0.03
0.05
Date (YearWeek)

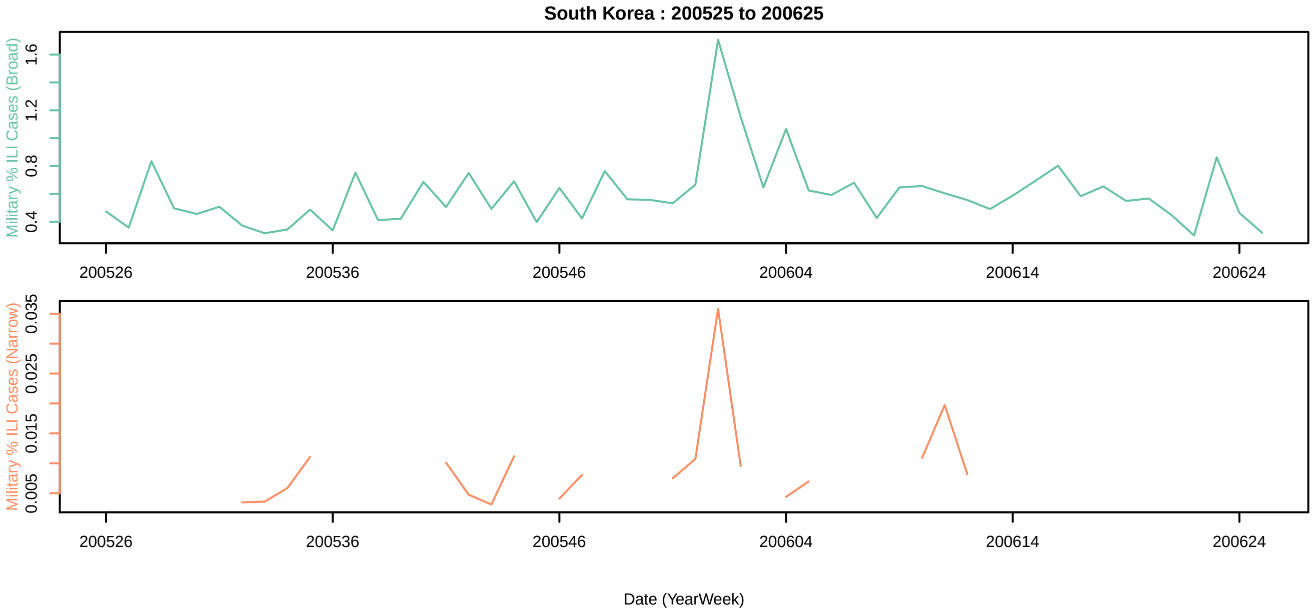

South Korea : 200525 to 200625
Military % ILI Cases (Broad)
0.4
0.8
1.2
1.6
200526
200536
200546
200604
200614
200624
Military % ILI Cases (Narrow)
0.005
0.015
0.025
0.035
200526
200536
200546
200604
200614
200624
Date (YearWeek)

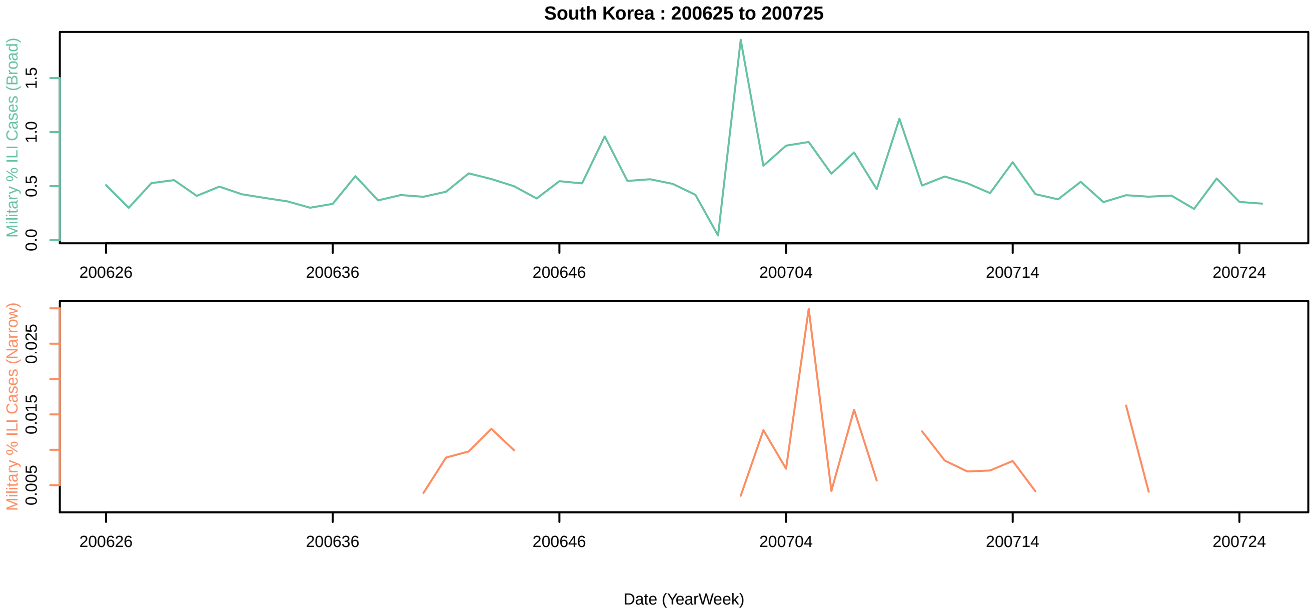
South Korea : 200625 to 200725
Military % ILI Cases (Broad)
0.0
0.5
1.0
1.5
200626
200636
200646
200704
200714
200724
Military % ILI Cases (Narrow)
0.005
0.015
0.025
200626
200636
200646
200704
200714
200724
Date (YearWeek)

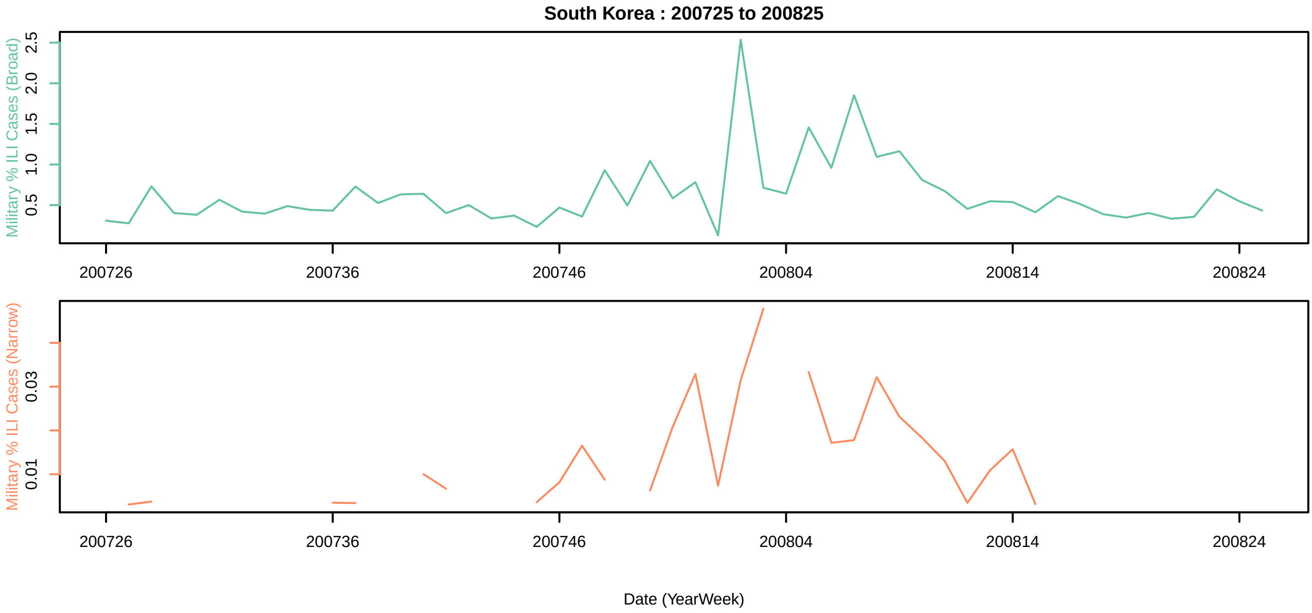
South Korea : 200725 to 200825
Military % ILI Cases (Broad)
0.5
1.0
1.5
2.0
2.5
200726
200736
200746
200804
200814
200824
Military % ILI Cases (Narrow)
0.01
0.03
200726
200736
200746
200804
200814
200824
Date (YearWeek)

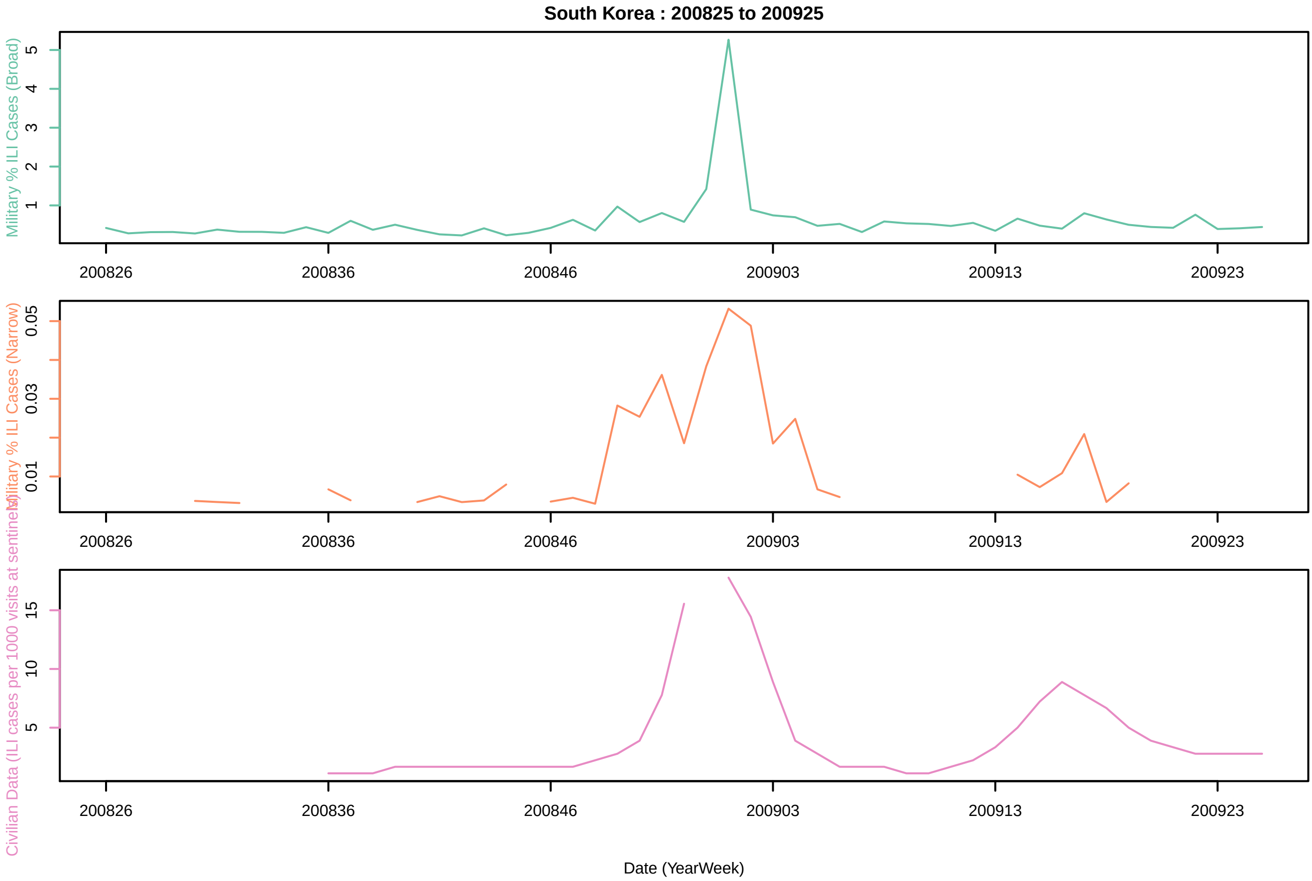
South Korea : 200825 to 200925
Military % ILI Cases (Broad)
Military % ILI Cases (Narrow)
Civilian Data (ILI cases per 1000 visits at sentinels)
200826
200836
200846
200903
200913
200923
Date (YearWeek)

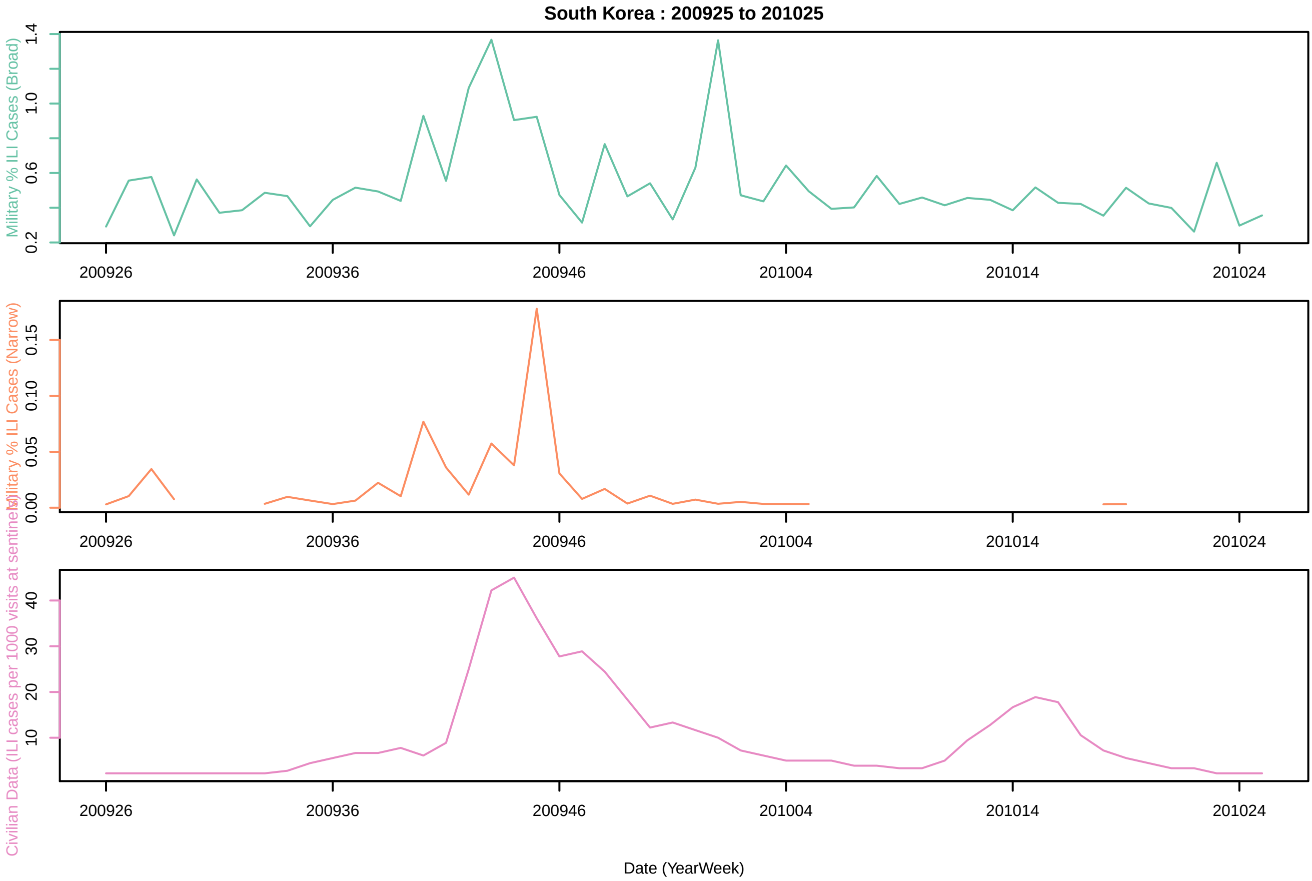
South Korea : 200925 to 201025
Military % ILI Cases (Broad)
0.2
0.6
1.0
1.4
Military % ILI Cases (Narrow)
0.00
0.05
0.10
0.15
Civilian Data (ILI cases per 1000 visits at sentinels)
10
20
30
40
200926
200936
200946
201004
201014
201024
Date (YearWeek)

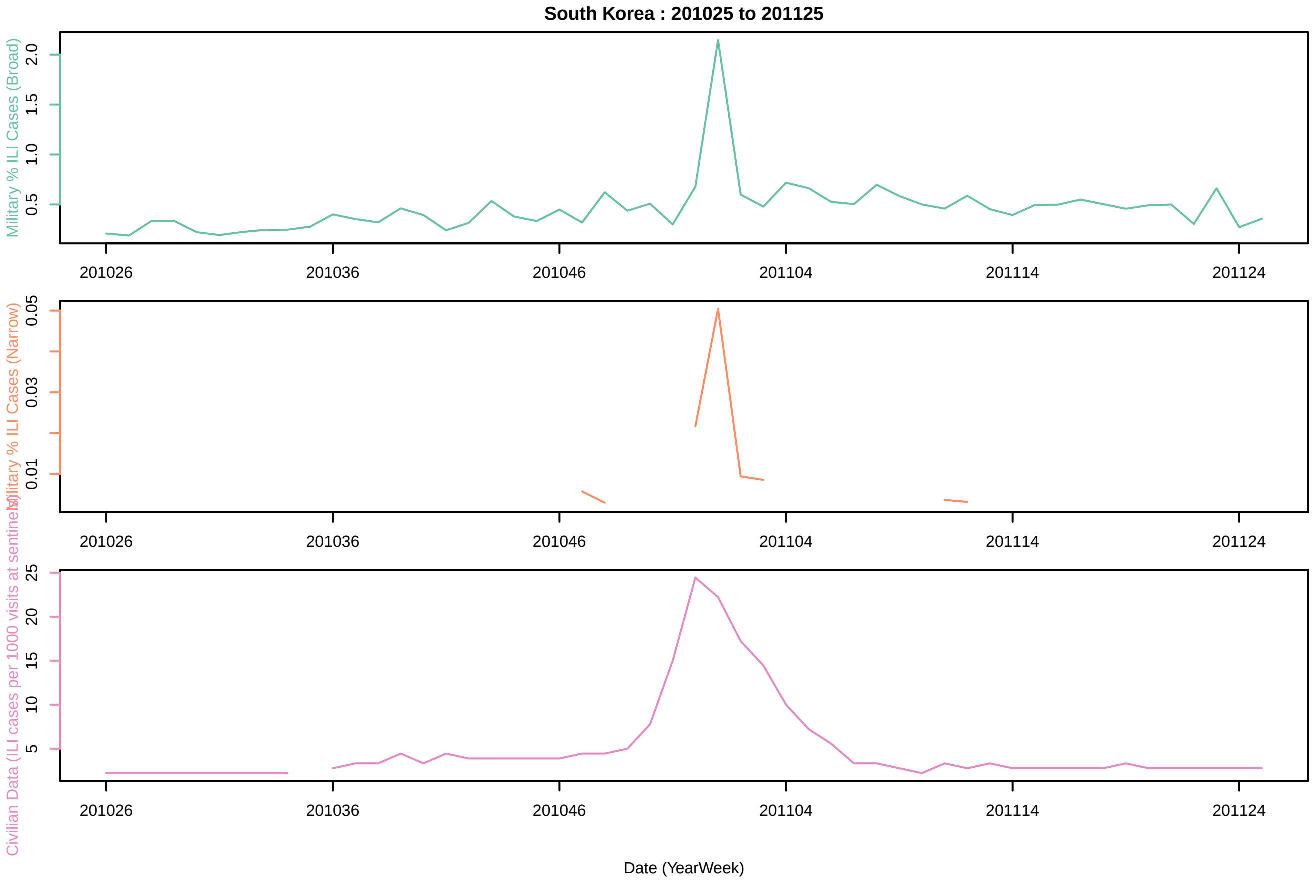

South Korea : 201025 to 201125
Military % ILI Cases (Broad)
0.5
1.0
1.5
2.0
201026
201036
201046
201104
201114
201124
Military % ILI Cases (Narrow)
0.01
0.03
0.05
201026
201036
201046
201104
201114
201124
Civilian Data (ILI cases per 1000 visits at sentinels)
5
10
15
20
25
201026
201036
201046
201104
201114
201124
Date (YearWeek)

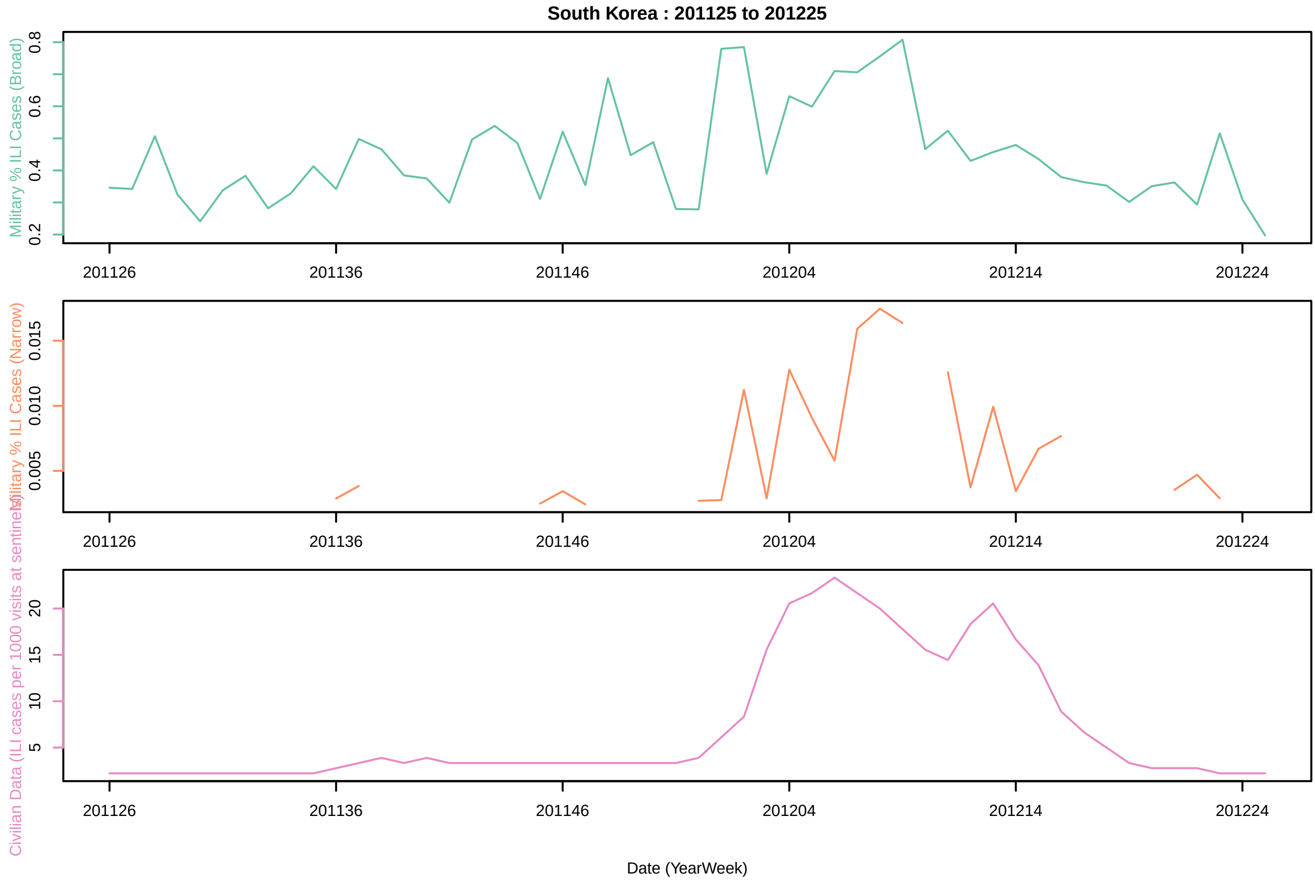
South Korea : 201125 to 201225
Military % ILI Cases (Broad)
0.2
0.4
0.6
0.8
Military % ILI Cases (Narrow)
0.005
0.010
0.015
Civilian Data (ILI cases per 1000 visits at sentinels)
5
10
15
20
201126
201136
201146
201204
201214
201224
Date (YearWeek)

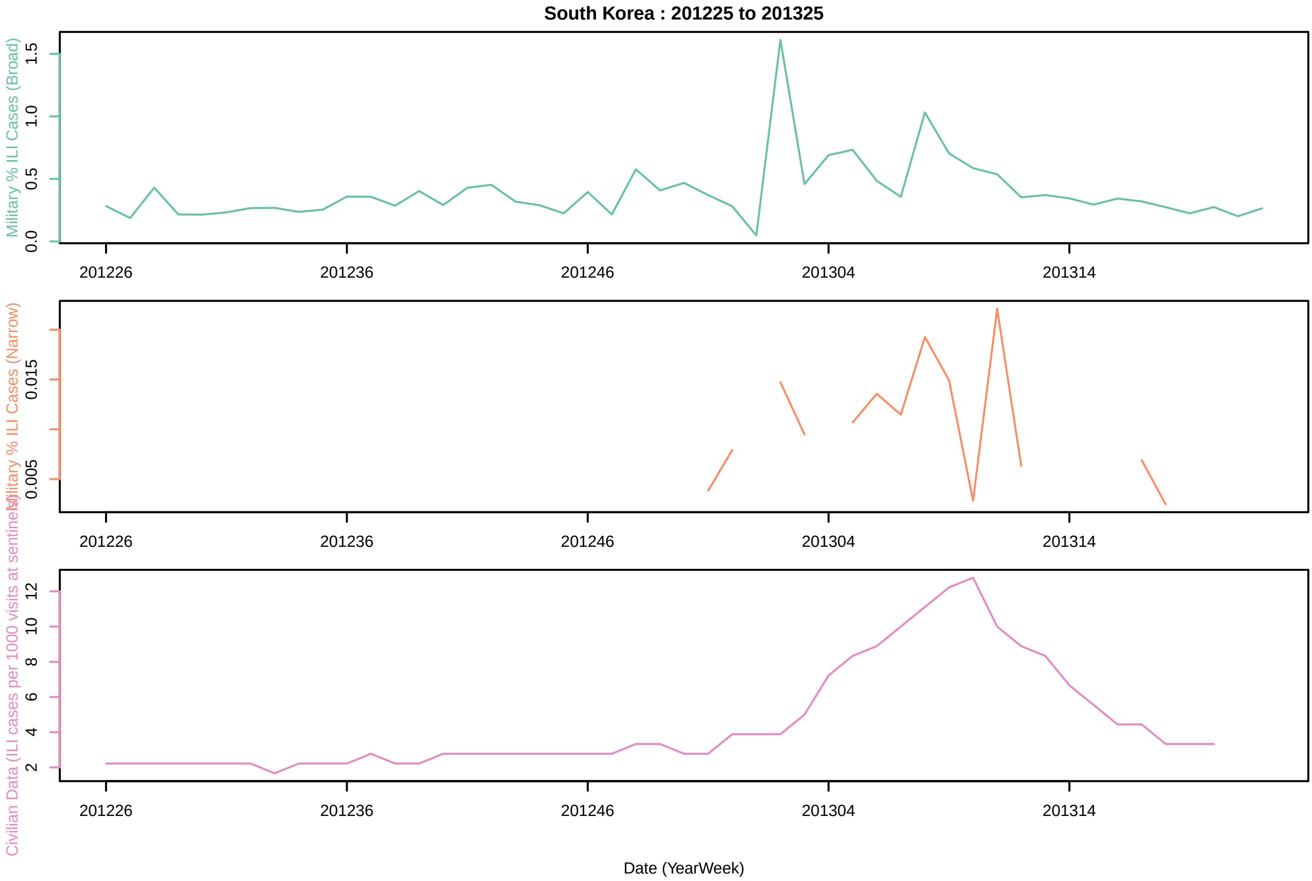
South Korea : 201225 to 201325
Military % ILI Cases (Broad)
Military % ILI Cases (Narrow)
Civilian Data (ILI cases per 1000 visits at sentinels)
Date (YearWeek)
201226
201236
201246
201304
201314

North Carolina : 199925 to 200025

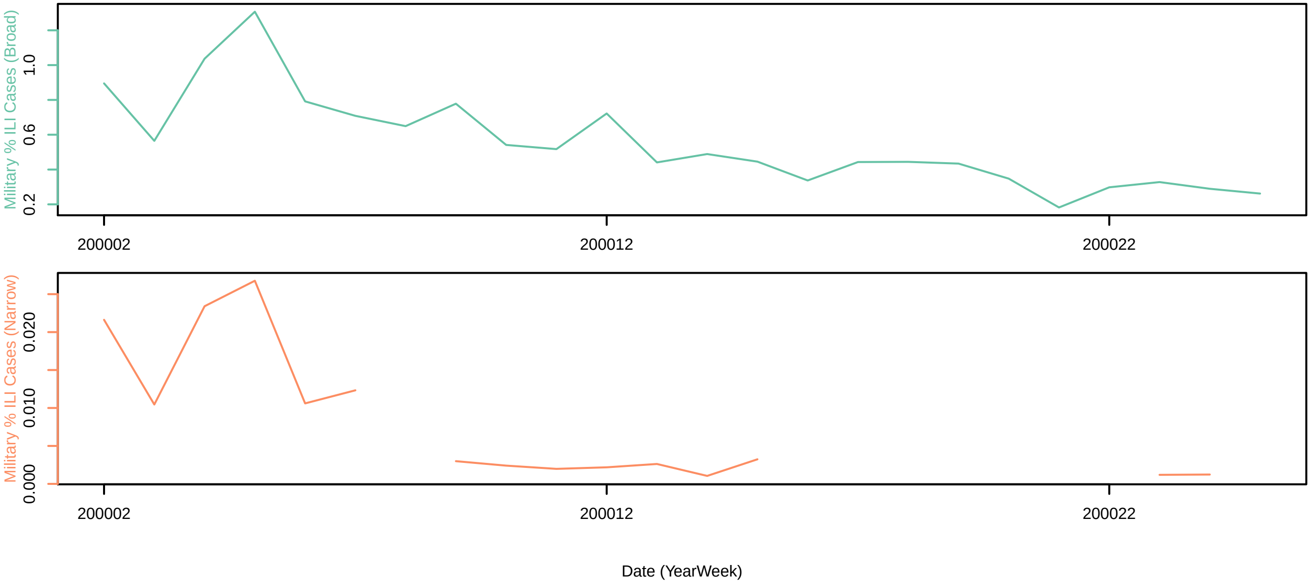

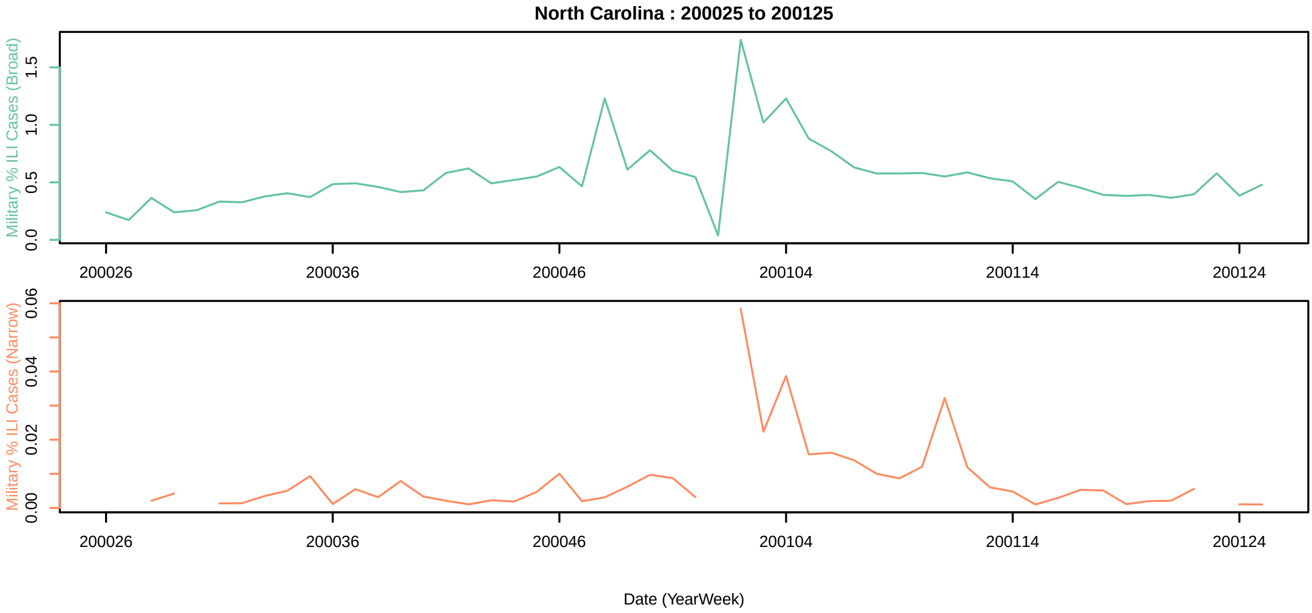
North Carolina : 200025 to 200125
Military % ILI Cases (Broad)
0.0
0.5
1.0
1.5
200026
200036
200046
200104
200114
200124
Military % ILI Cases (Narrow)
0.00
0.02
0.04
0.06
200026
200036
200046
200104
200114
200124
Date (YearWeek)

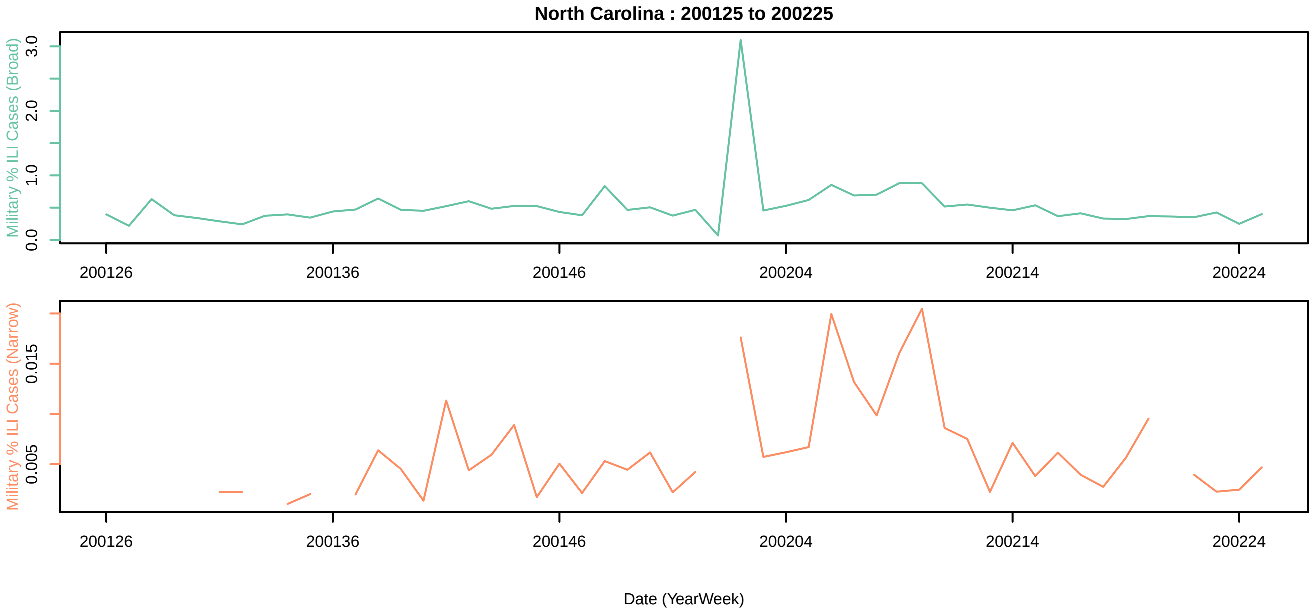

North Carolina : 200125 to 200225
Military % ILI Cases (Broad)
0.0
1.0
2.0
3.0
200126
200136
200146
200204
200214
200224
Military % ILI Cases (Narrow)
0.005
0.015
200126
200136
200146
200204
200214
200224
Date (YearWeek)

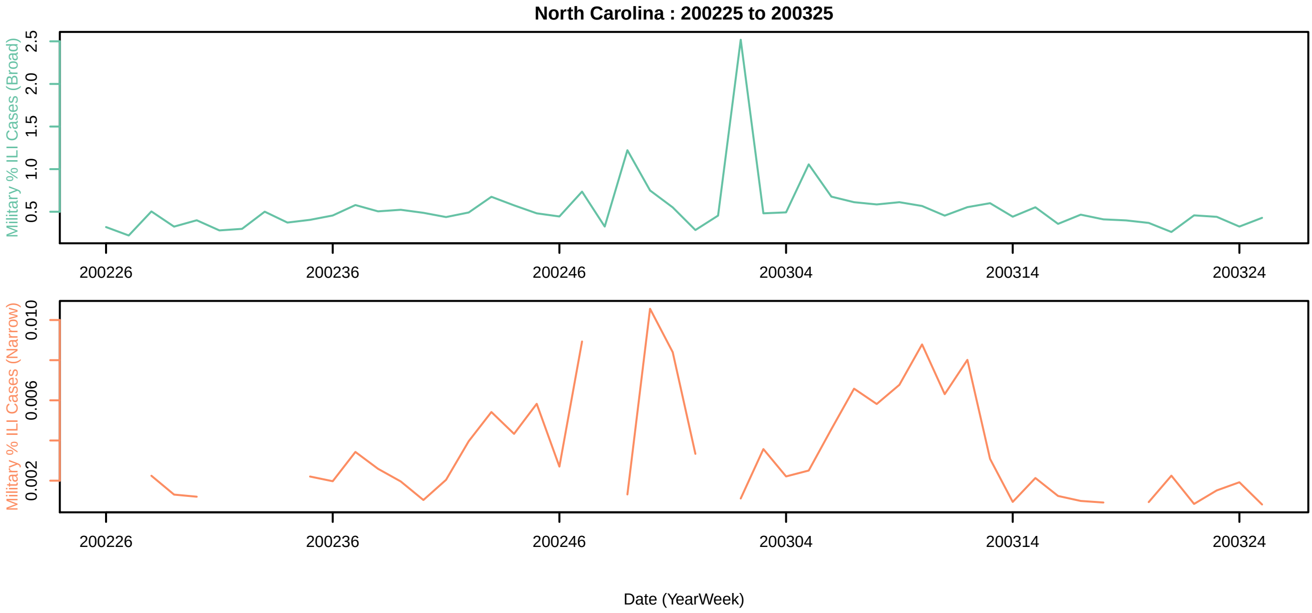
North Carolina : 200225 to 200325
Military % ILI Cases (Broad)
0.5
1.0
1.5
2.0
2.5
200226
200236
200246
200304
200314
200324
Military % ILI Cases (Narrow)
0.002
0.006
0.010
200226
200236
200246
200304
200314
200324
Date (YearWeek)

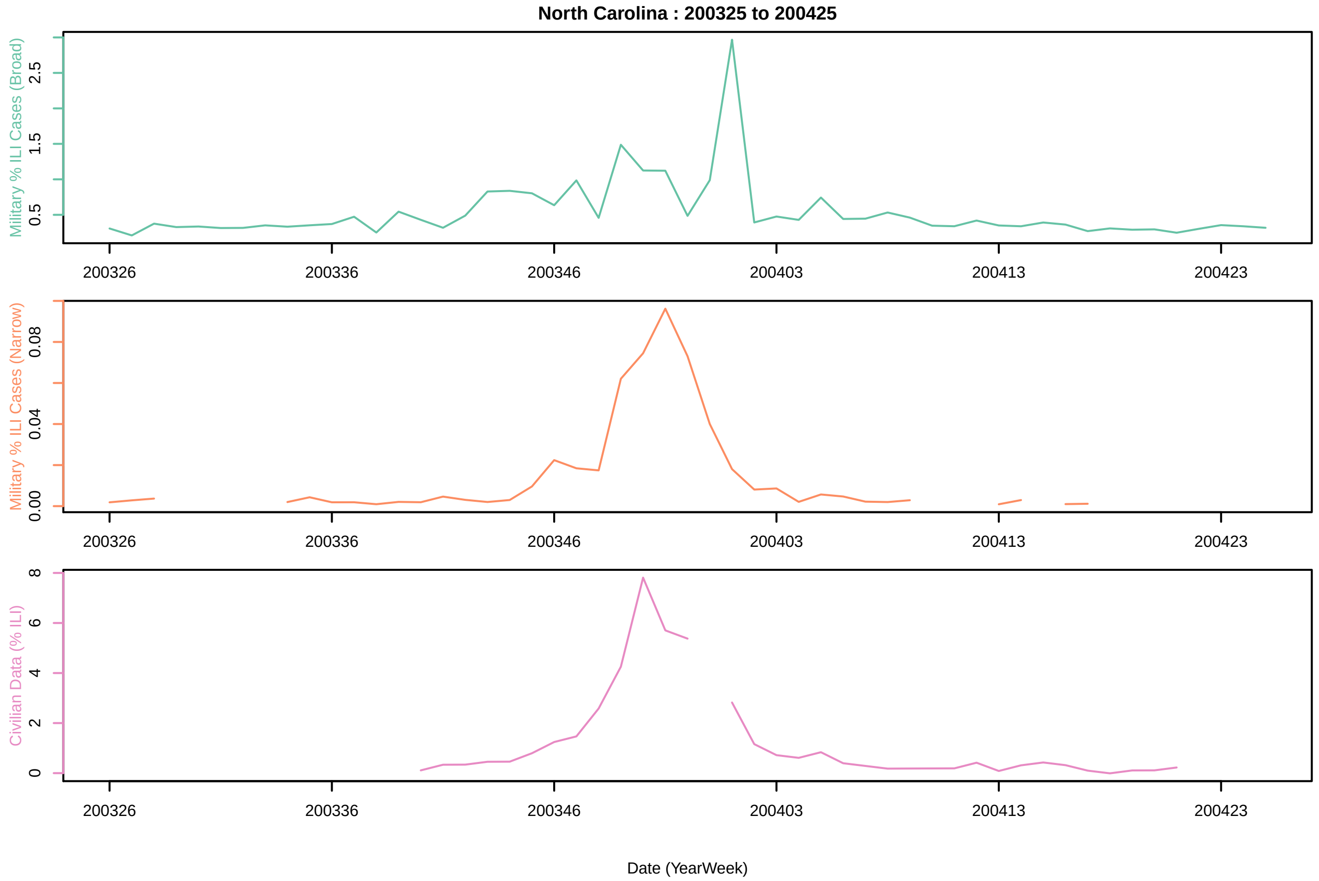
North Carolina : 200325 to 200425
Military % ILI Cases (Broad)
0.5
1.5
2.5
Military % ILI Cases (Narrow)
0.00
0.04
0.08
Civilian Data (% ILI)
0
2
4
6
8
200326
200336
200346
200403
200413
200423
Date (YearWeek)

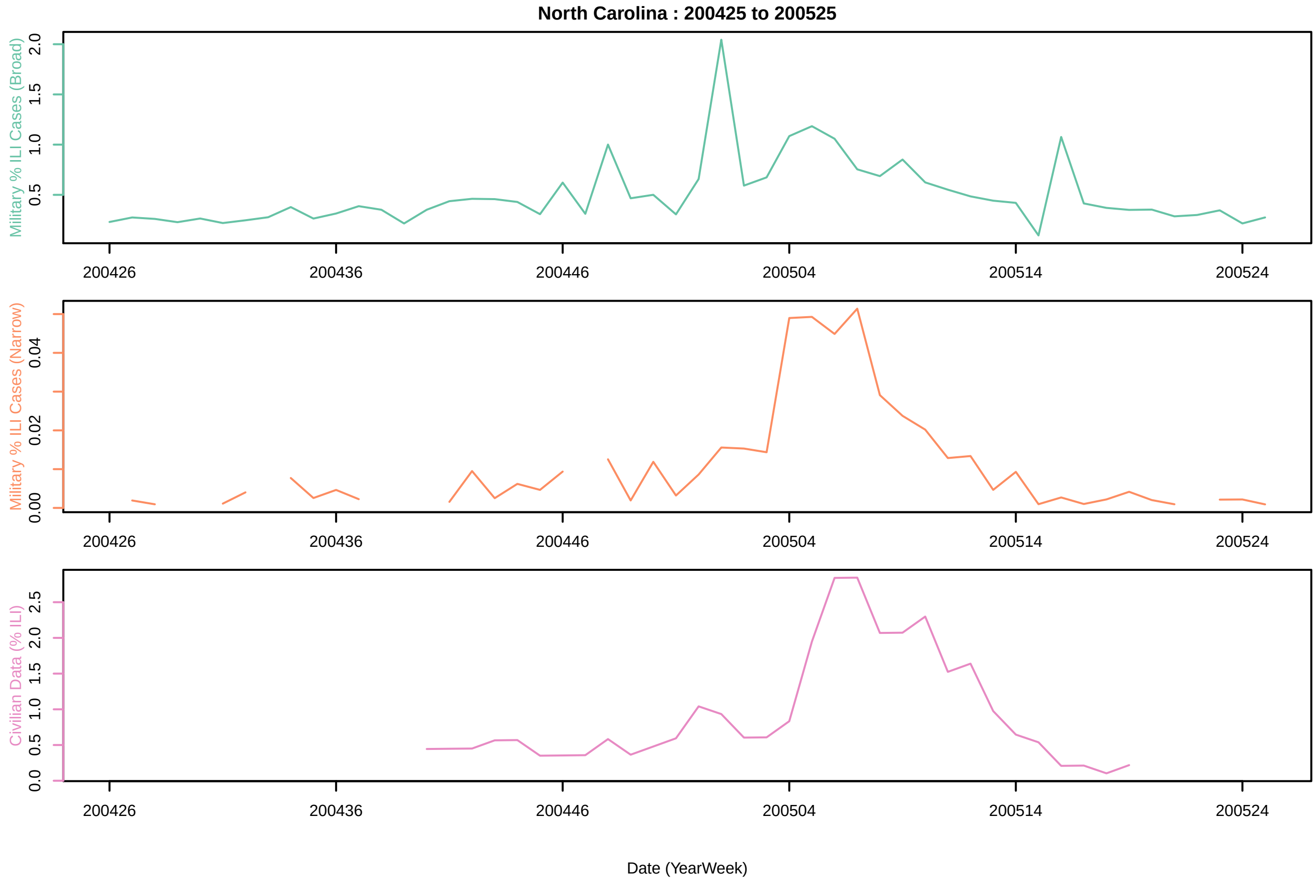

North Carolina : 200425 to 200525
Military % ILI Cases (Broad)
0.5
1.0
1.5
2.0
200426
200436
200446
200504
200514
200524
Military % ILI Cases (Narrow)
0.00
0.02
0.04
Civilian Data (% ILI)
0.0
0.5
1.0
1.5
2.0
2.5
Date (YearWeek)

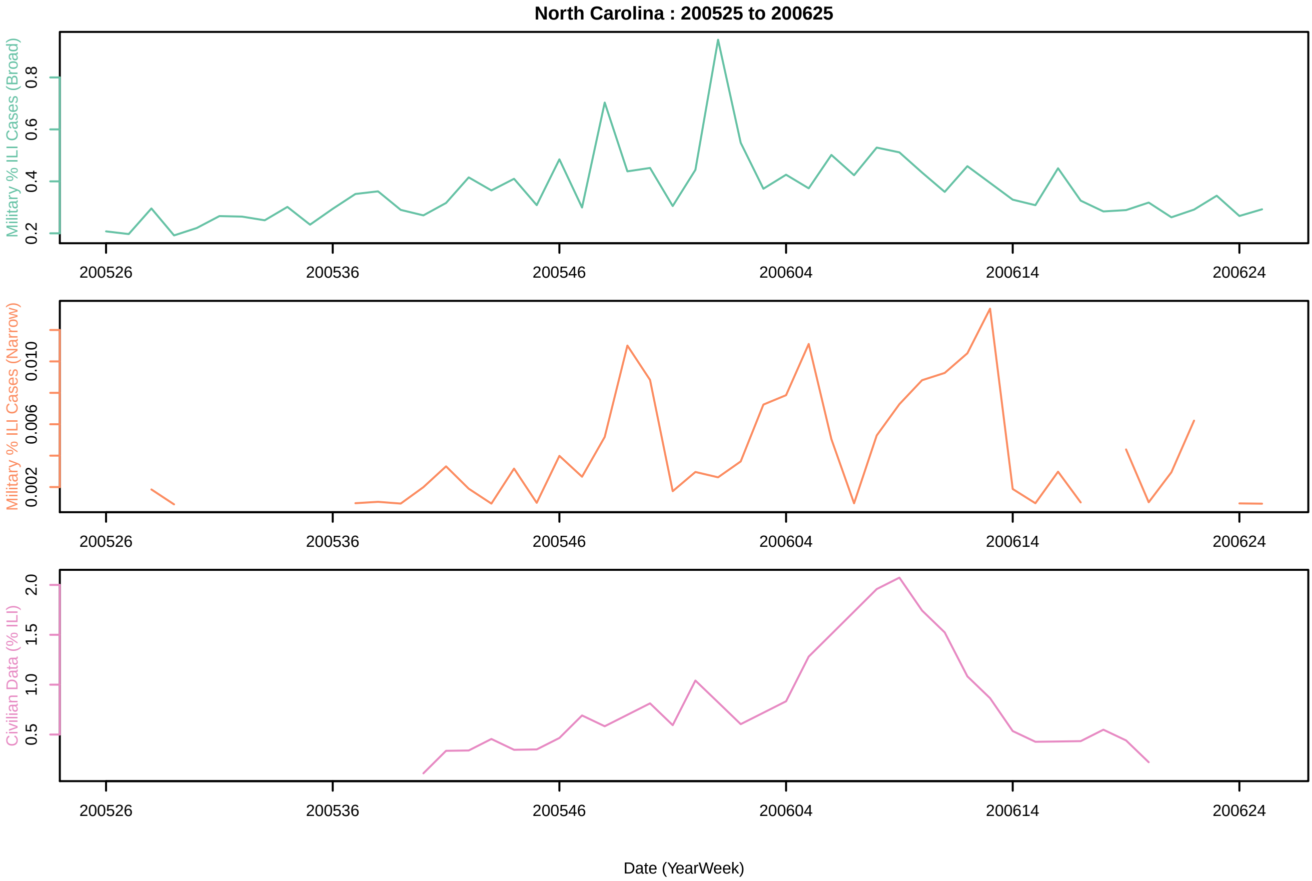

North Carolina : 200525 to 200625
Military % ILI Cases (Broad)
0.2
0.4
0.6
0.8
Military % ILI Cases (Narrow)
0.002
0.006
0.010
Civilian Data (% ILI)
0.5
1.0
1.5
2.0
200526
200536
200546
200604
200614
200624
Date (YearWeek)

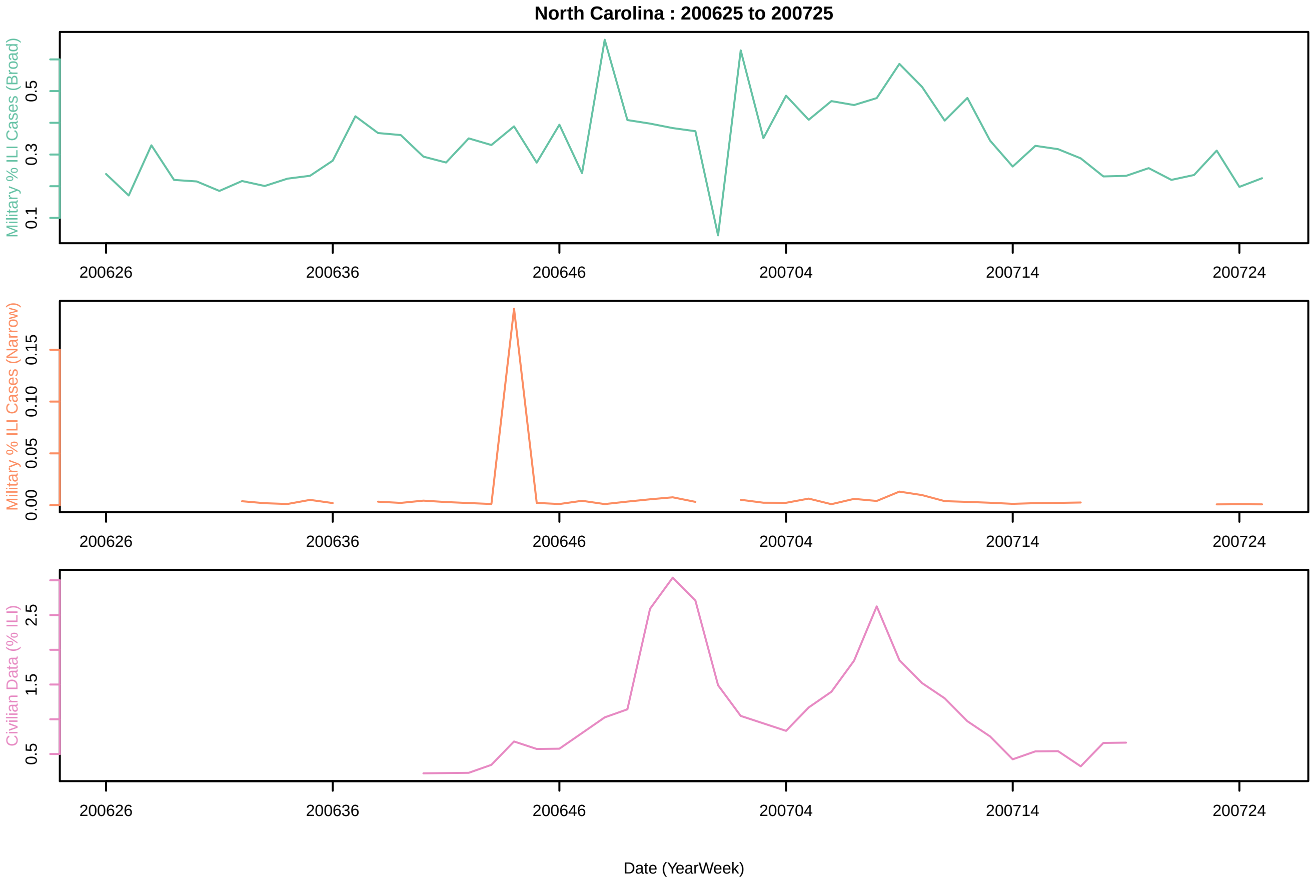
North Carolina : 200625 to 200725
Military % ILI Cases (Broad)
0.1
0.3
0.5
Military % ILI Cases (Narrow)
0.00
0.05
0.10
0.15
Civilian Data (% ILI)
0.5
1.5
2.5
200626
200636
200646
200704
200714
200724
Date (YearWeek)

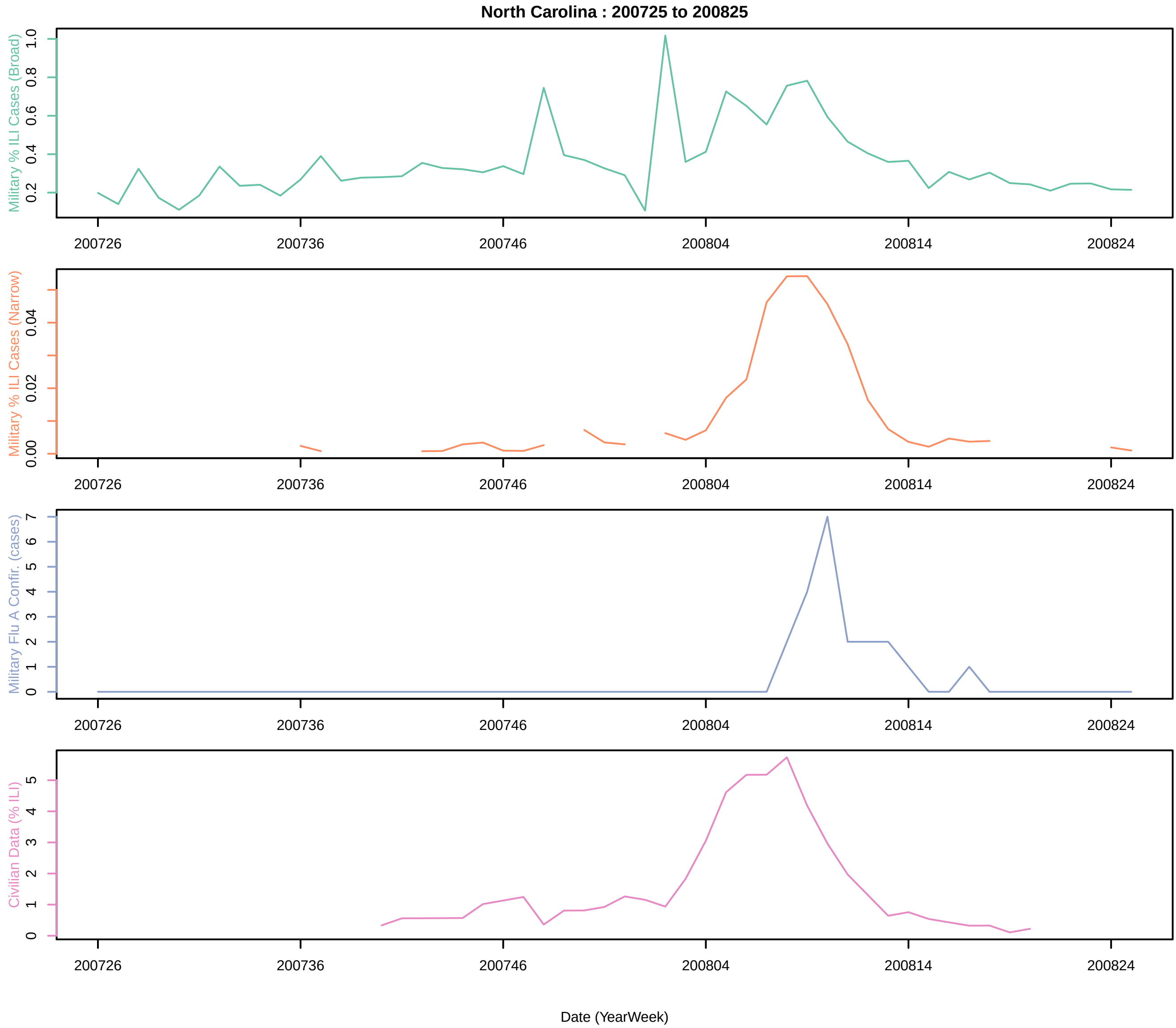
North Carolina : 200725 to 200825
Military % ILI Cases (Broad)
Military % ILI Cases (Narrow)
Military Flu A Confir. (cases)
Civilian Data (% ILI)
200726
200736
200746
200804
200814
200824
Date (YearWeek)

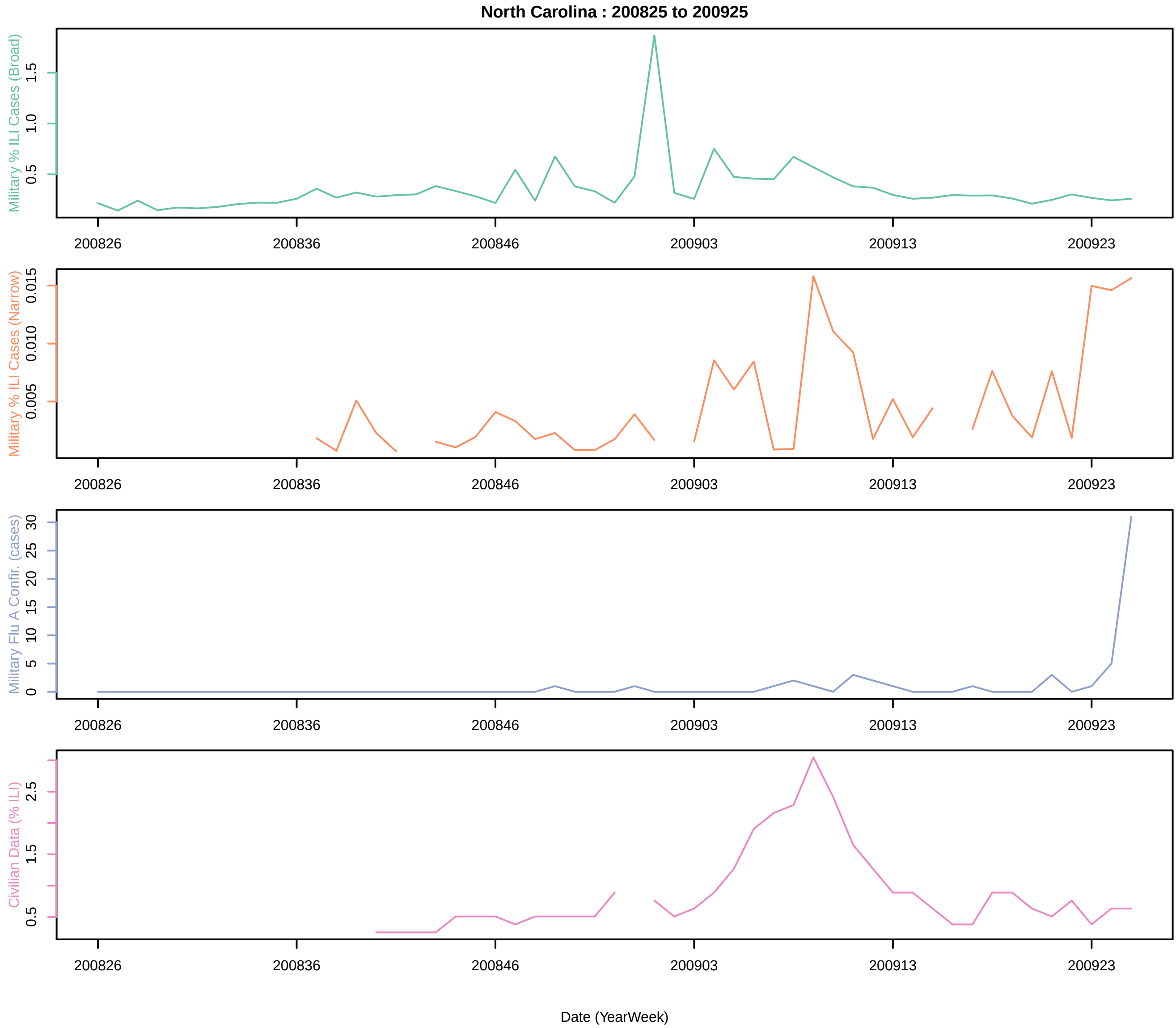
North Carolina : 200825 to 200925
Military % ILI Cases (Broad)
Military % ILI Cases (Narrow)
Military Flu A Confir. (cases)
Civilian Data (% ILI)
200826
200836
200846
200903
200913
200923
Date (YearWeek)

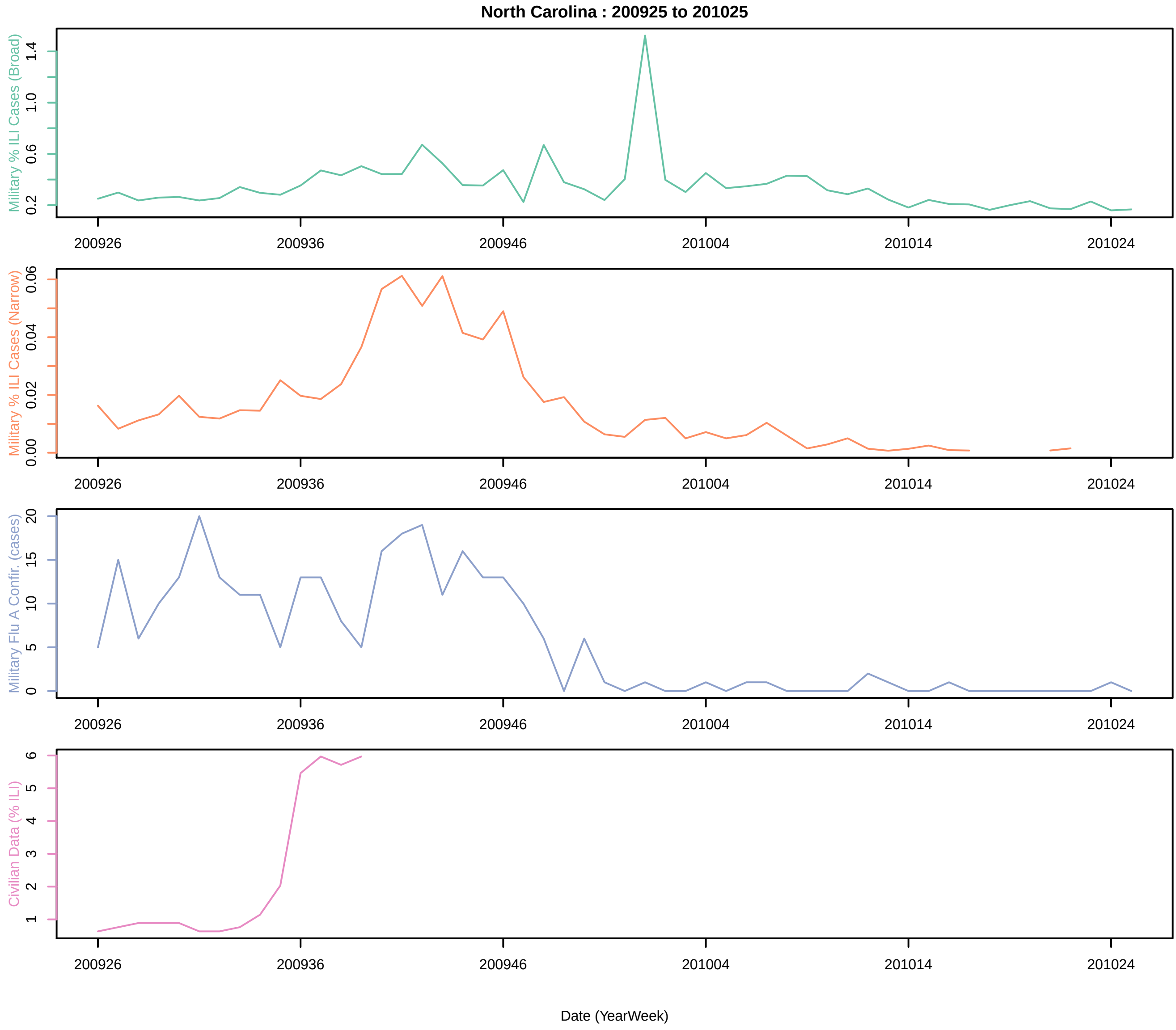
North Carolina : 200925 to 201025
Military % ILI Cases (Broad)
0.2
0.6
1.0
1.4
Military % ILI Cases (Narrow)
0.00
0.02
0.04
0.06
Military Flu A Confir. (cases)
0
5
10
15
20
Civilian Data (% ILI)
1
2
3
4
5
6
200926
200936
200946
201004
201014
201024
Date (YearWeek)

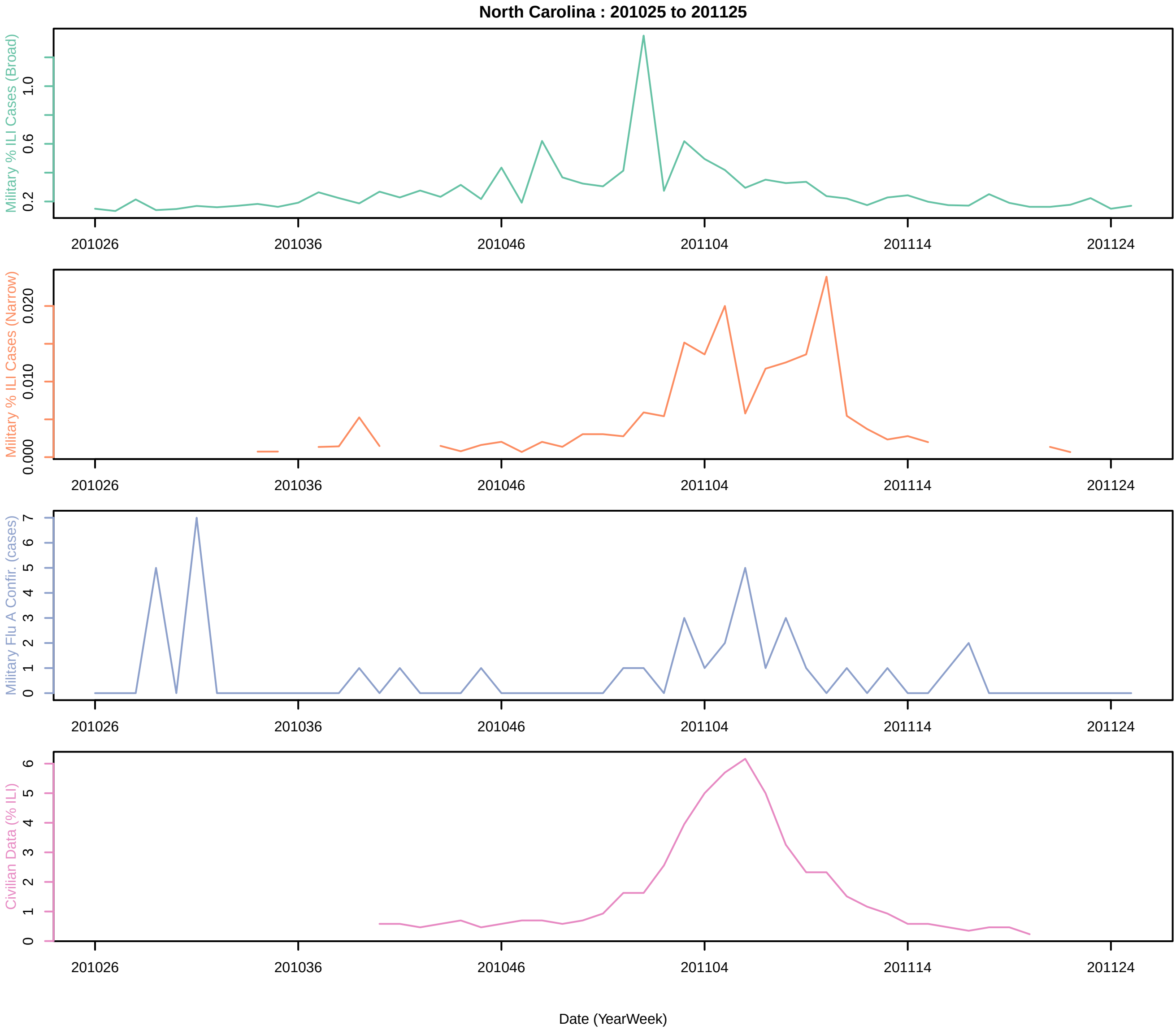

North Carolina : 201025 to 201125
Military % ILI Cases (Broad)
0.2
0.6
1.0
Military % ILI Cases (Narrow)
0.000
0.010
0.020
Military Flu A Confir. (cases)
0
1
2
3
4
5
6
7
Civilian Data (% ILI)
0
1
2
3
4
5
6
201026
201036
201046
201104
201114
201124
Date (YearWeek)

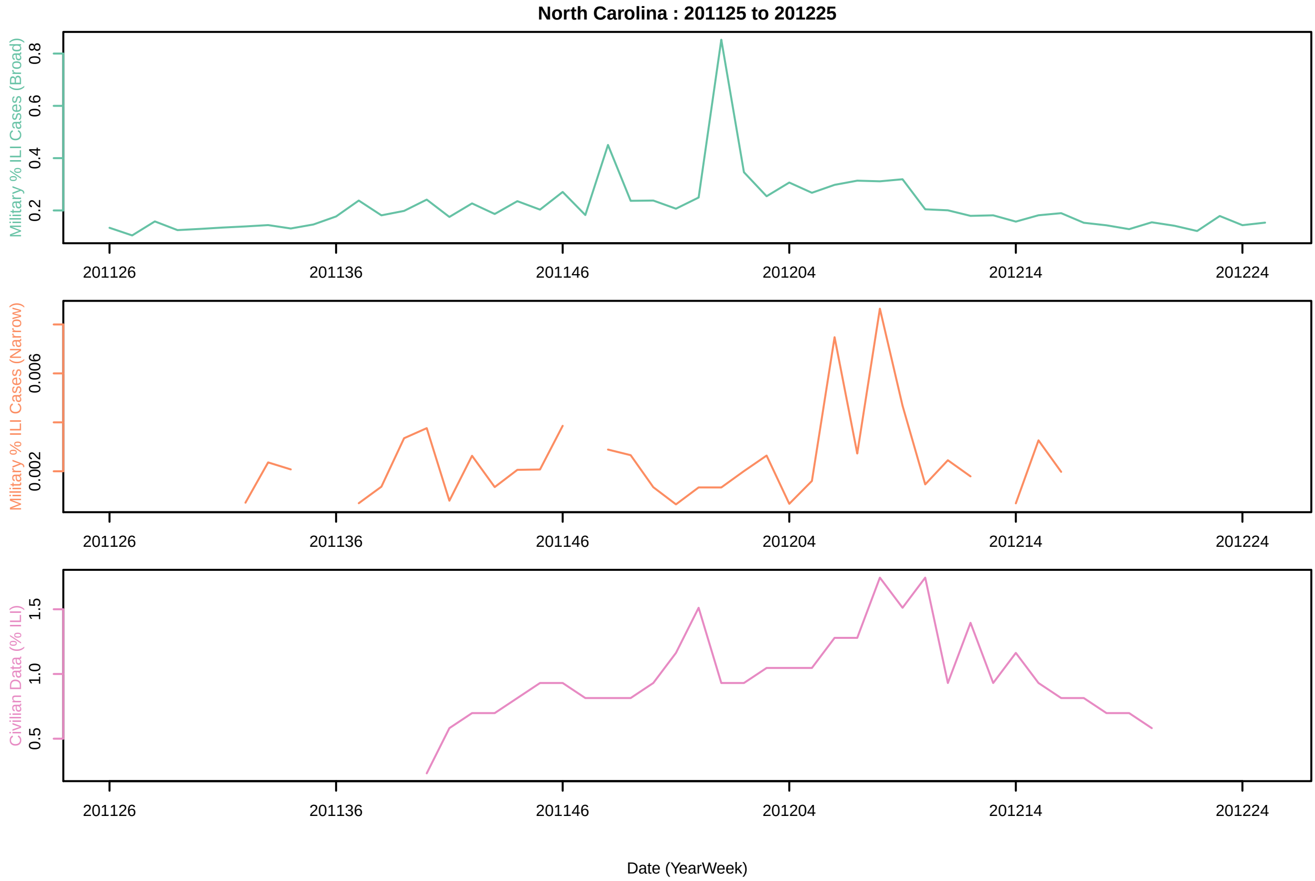

North Carolina : 201125 to 201225
Military % ILI Cases (Broad)
0.2
0.4
0.6
0.8
201126
201136
201146
201204
201214
201224
Military % ILI Cases (Narrow)
0.002
0.006
201126
201136
201146
201204
201214
201224
Civilian Data (% ILI)
0.5
1.0
1.5
201126
201136
201146
201204
201214
201224
Date (YearWeek)

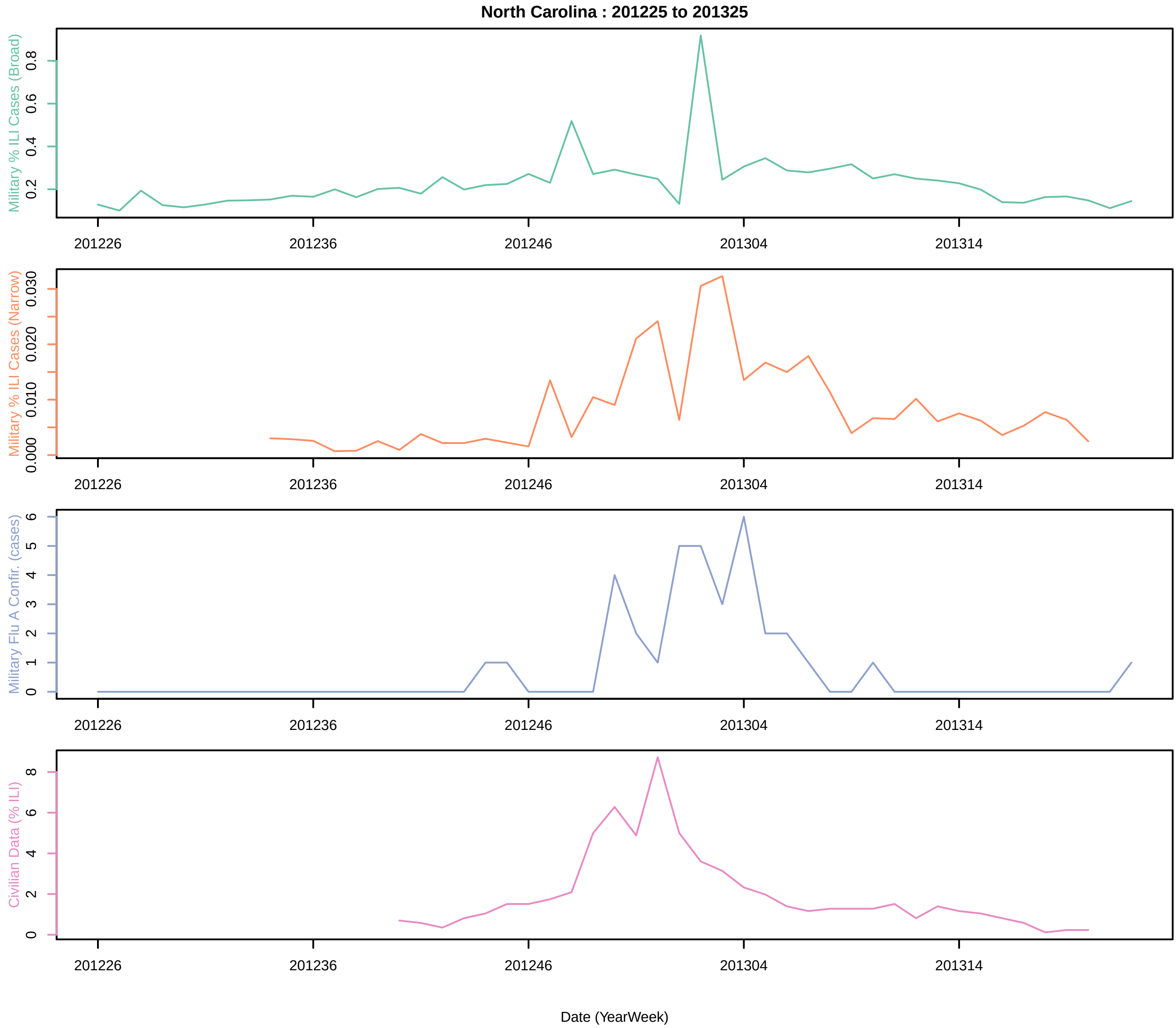
North Carolina : 201225 to 201325
Military % ILI Cases (Broad)
0.2
0.4
0.6
0.8
Military % ILI Cases (Narrow)
0.000
0.010
0.020
0.030
Military Flu A Confir. (cases)
0
1
2
3
4
5
6
Civilian Data (% ILI)
0
2
4
6
8
201226
201236
201246
201304
201314
Date (YearWeek)

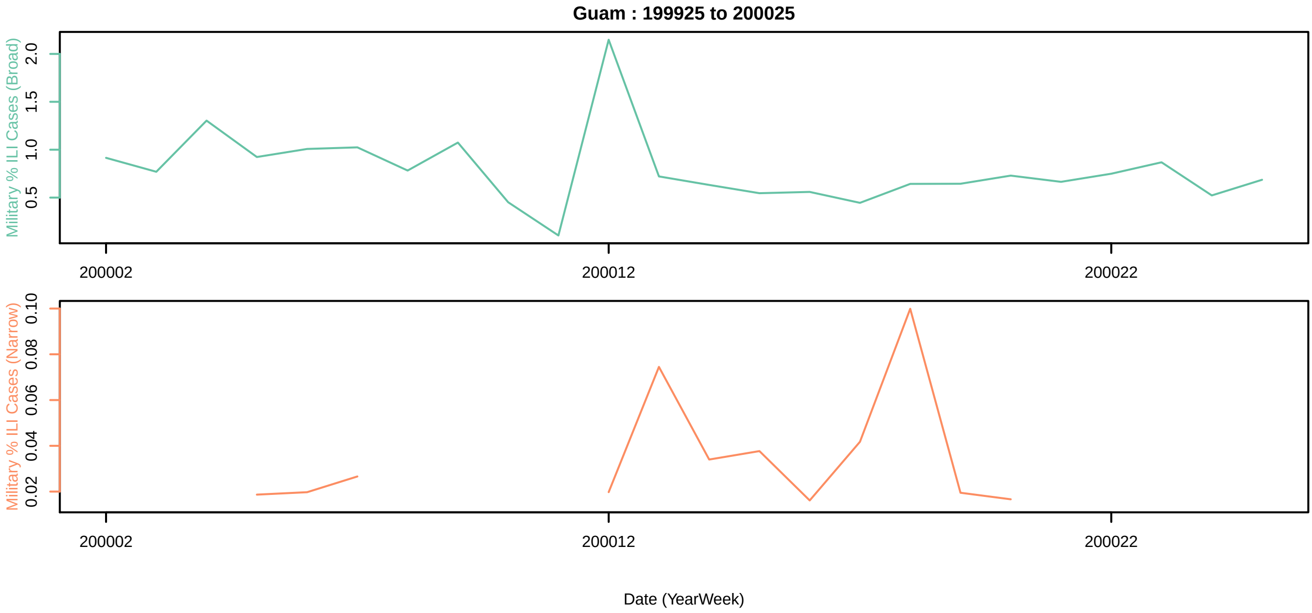
Guam : 199925 to 200025
Military % ILI Cases (Broad)
0.5
1.0
1.5
2.0
200002
200012
200022
Military % ILI Cases (Narrow)
0.02
0.04
0.06
0.08
0.10
200002
200012
200022
Date (YearWeek)

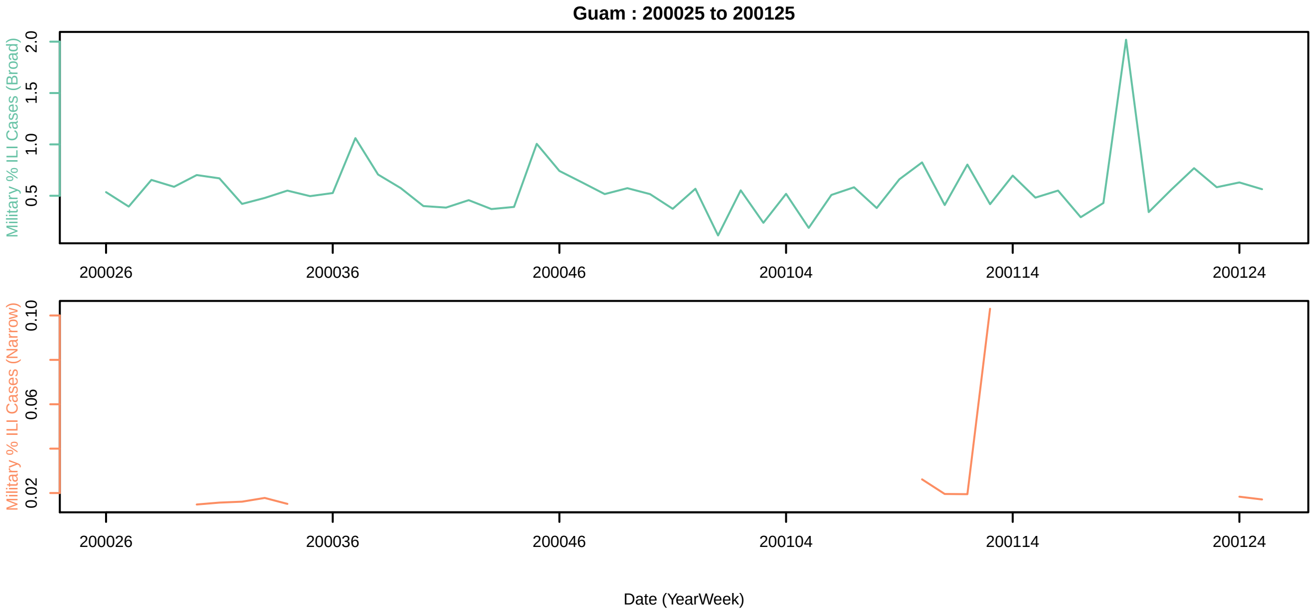
Guam : 200025 to 200125
Military % ILI Cases (Broad)
0.5
1.0
1.5
2.0
200026
200036
200046
200104
200114
200124
Military % ILI Cases (Narrow)
0.02
0.06
0.10
200026
200036
200046
200104
200114
200124
Date (YearWeek)

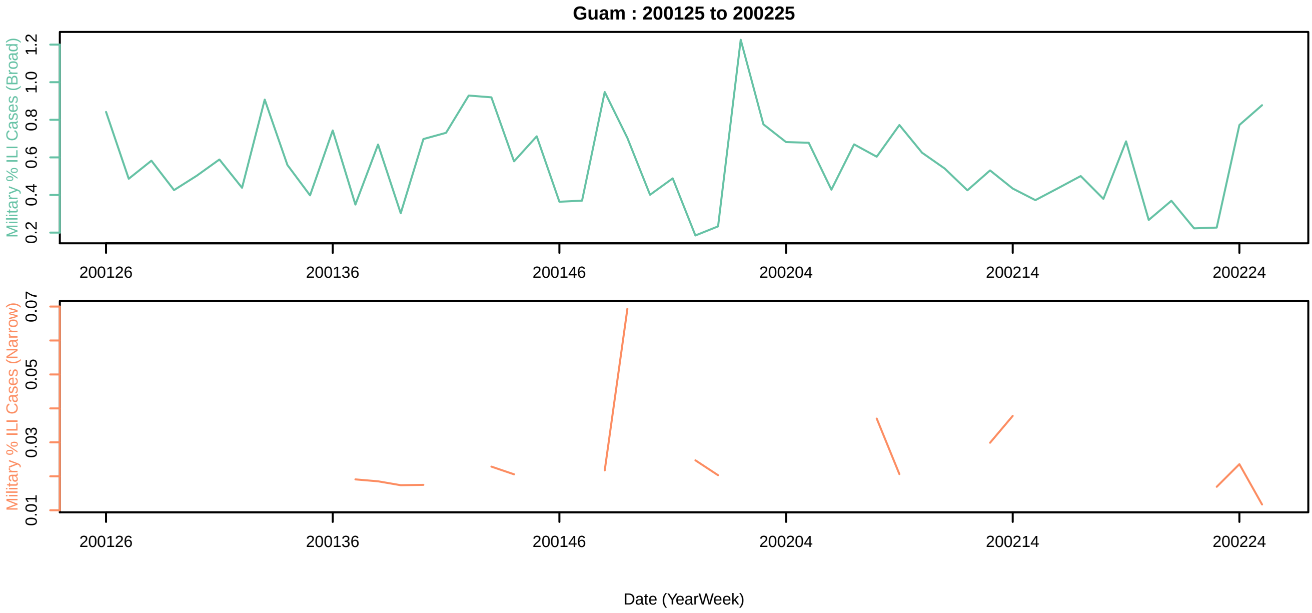

Guam : 200125 to 200225
Military % ILI Cases (Broad)
0.2
0.4
0.6
0.8
1.0
1.2
200126
200136
200146
200204
200214
200224
Military % ILI Cases (Narrow)
0.01
0.03
0.05
0.07
200126
200136
200146
200204
200214
200224
Date (YearWeek)

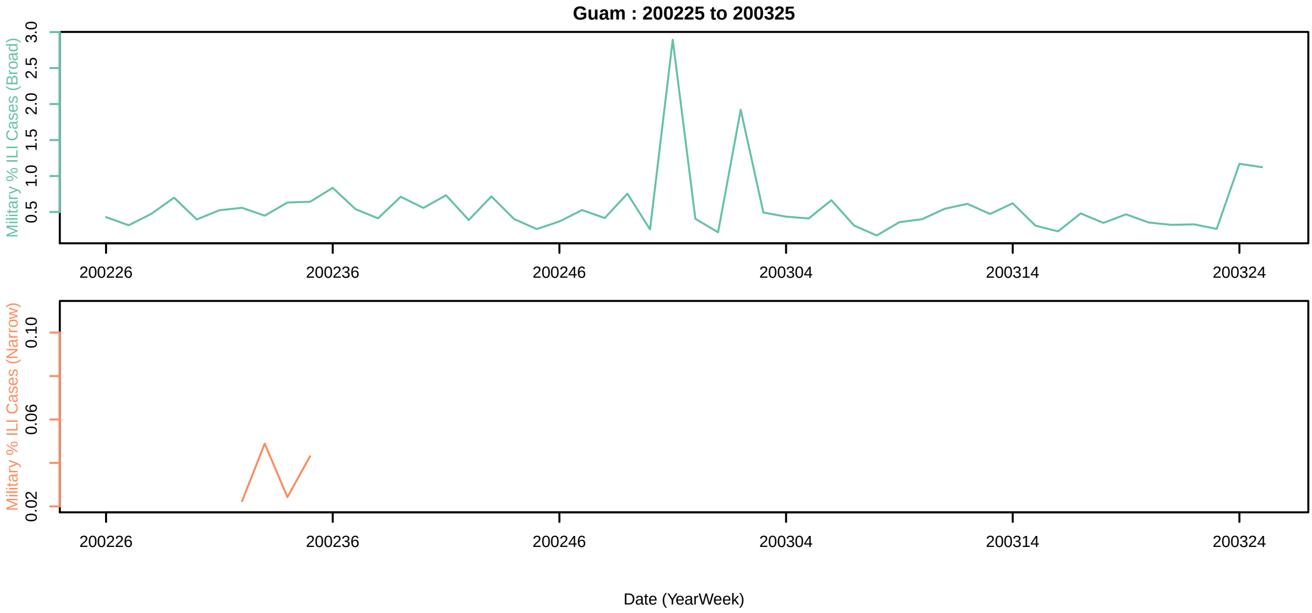

Guam : 200225 to 200325
Military % ILI Cases (Broad)
0.5
1.0
1.5
2.0
2.5
3.0
200226
200236
200246
200304
200314
200324
Military % ILI Cases (Narrow)
0.02
0.06
0.10
200226
200236
200246
200304
200314
200324
Date (YearWeek)

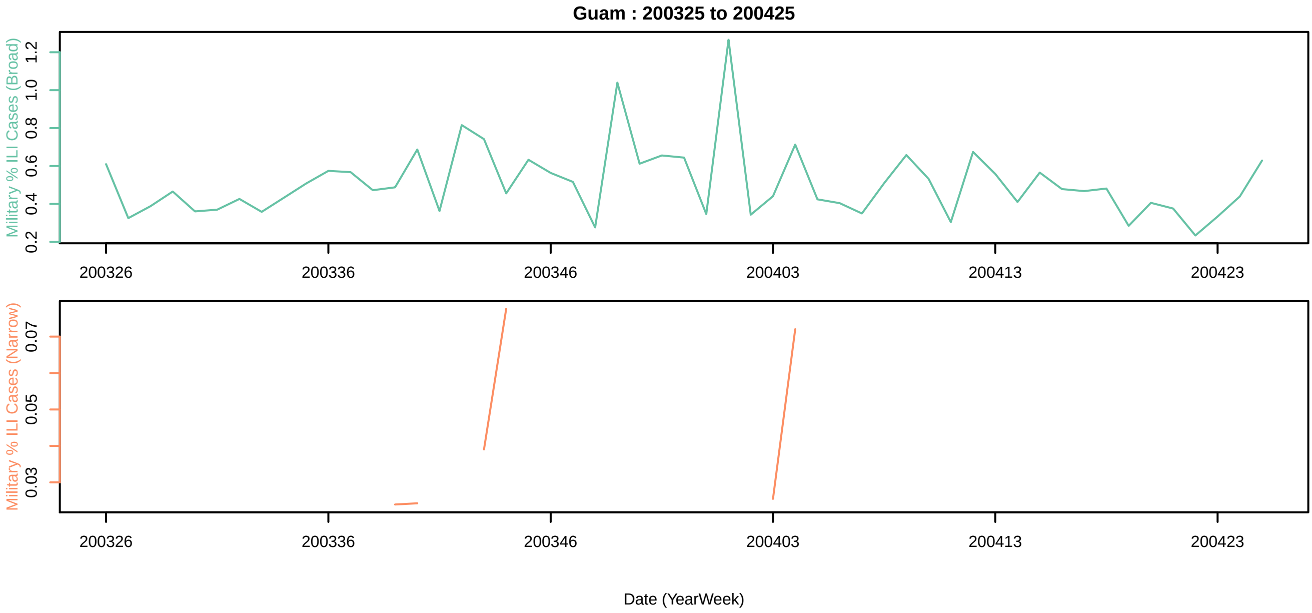
Guam : 200325 to 200425
Military % ILI Cases (Broad)
0.2
0.4
0.6
0.8
1.0
1.2
200326
200336
200346
200403
200413
200423
Military % ILI Cases (Narrow)
0.03
0.05
0.07
200326
200336
200346
200403
200413
200423
Date (YearWeek)

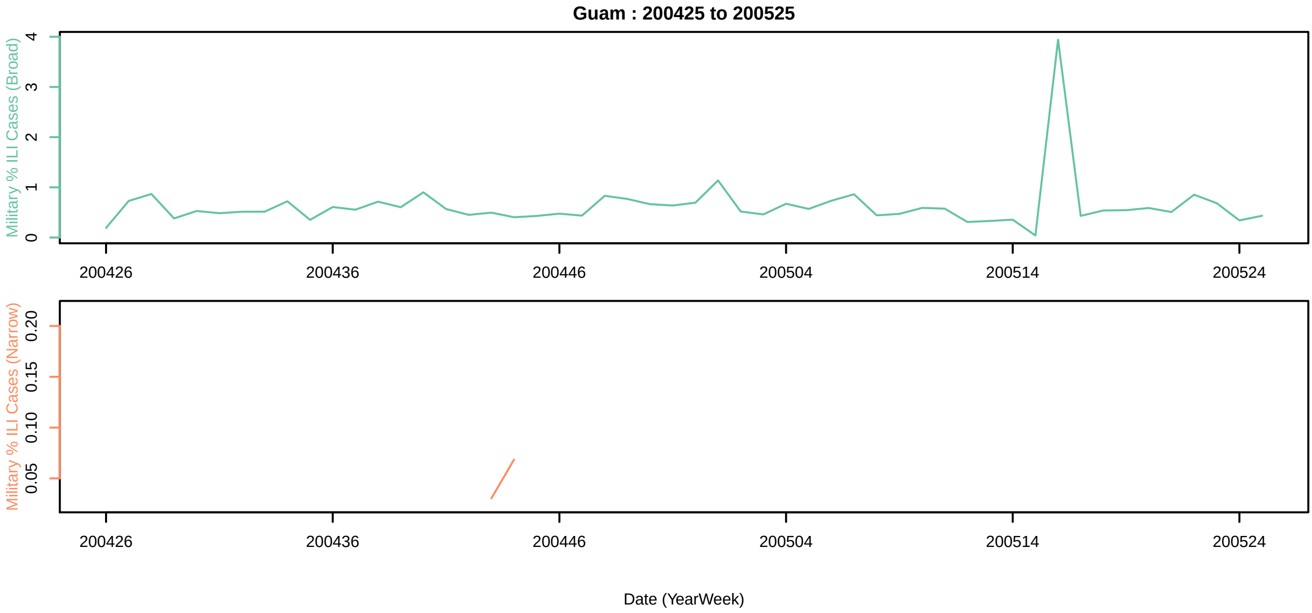
Guam : 200425 to 200525
Military % ILI Cases (Broad)
0
1
2
3
4
200426
200436
200446
200504
200514
200524
Military % ILI Cases (Narrow)
0.05
0.10
0.15
0.20
200426
200436
200446
200504
200514
200524
Date (YearWeek)

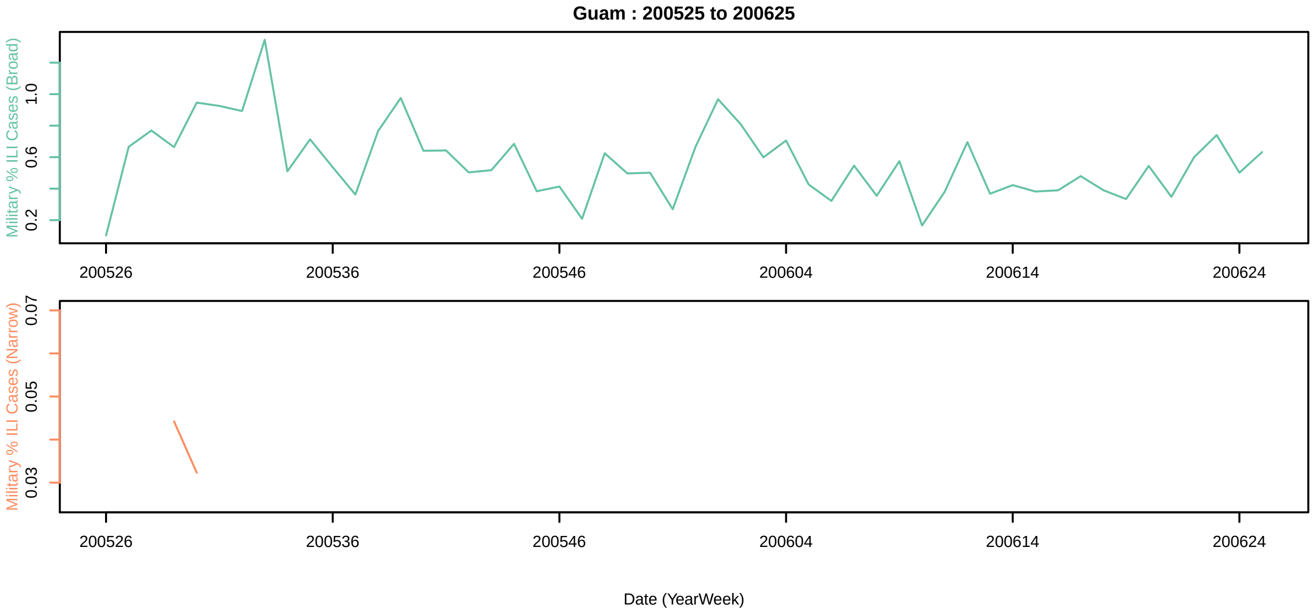

Guam : 200525 to 200625
Military % ILI Cases (Broad)
0.2
0.6
1.0
200526
200536
200546
200604
200614
200624
Military % ILI Cases (Narrow)
0.03
0.05
0.07
200526
200536
200546
200604
200614
200624
Date (YearWeek)

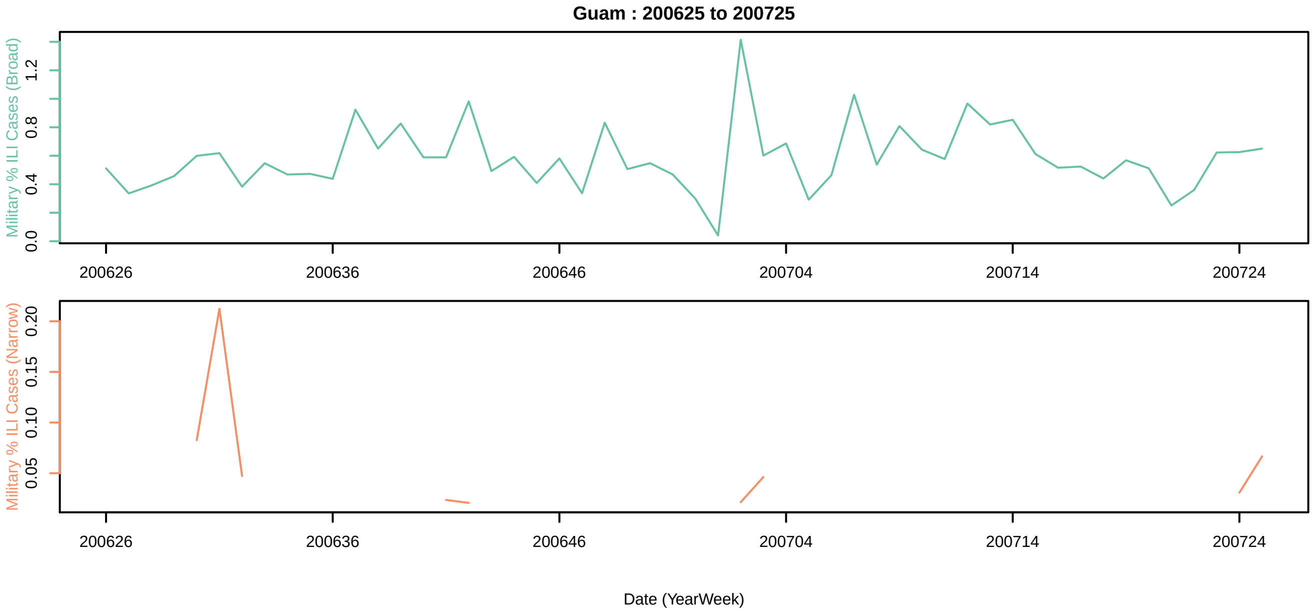
Guam : 200625 to 200725
Military % ILI Cases (Broad)
0.0
0.4
0.8
1.2
200626
200636
200646
200704
200714
200724
Military % ILI Cases (Narrow)
0.05
0.10
0.15
0.20
200626
200636
200646
200704
200714
200724
Date (YearWeek)

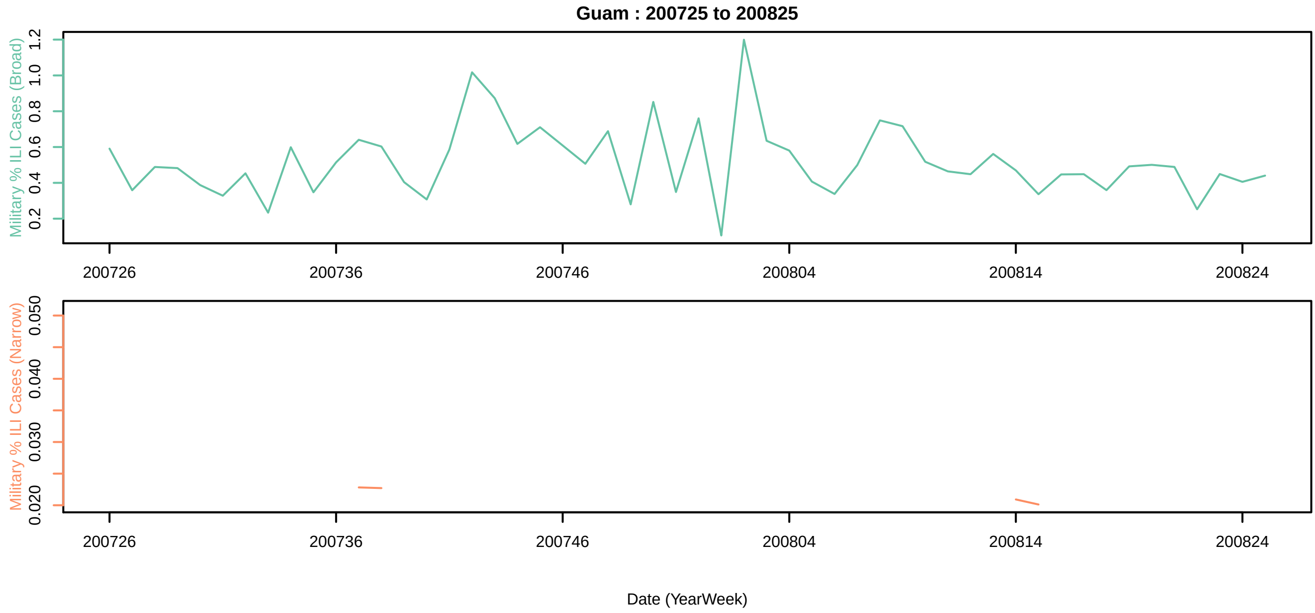

Guam : 200725 to 200825
Military % ILI Cases (Broad)
0.2
0.4
0.6
0.8
1.0
1.2
200726
200736
200746
200804
200814
200824
Military % ILI Cases (Narrow)
0.020
0.030
0.040
0.050
200726
200736
200746
200804
200814
200824
Date (YearWeek)

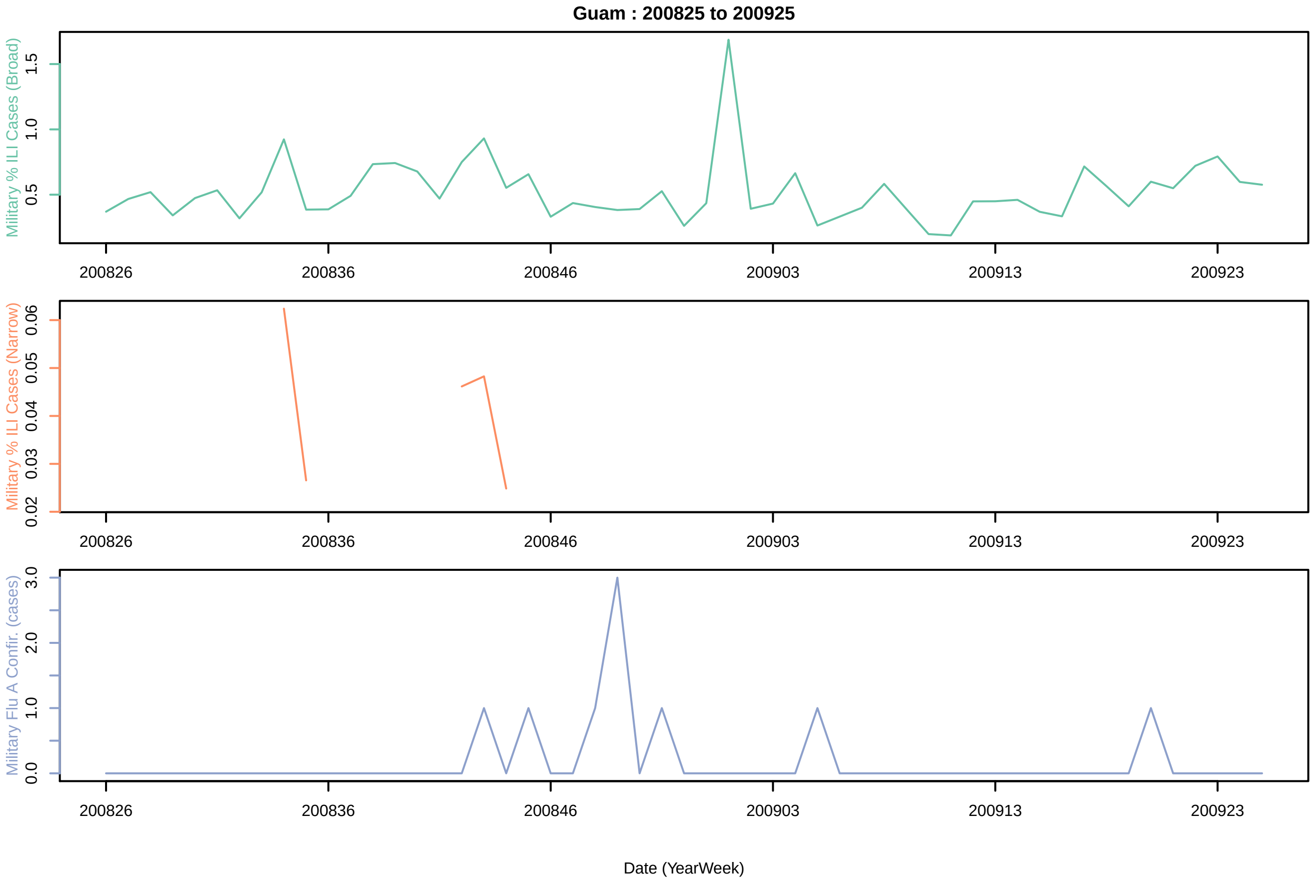

Guam : 200825 to 200925
Military % ILI Cases (Broad)
0.5
1.0
1.5
200826
200836
200846
200903
200913
200923
Military % ILI Cases (Narrow)
0.02
0.03
0.04
0.05
0.06
Military Flu A Confir. (cases)
0.0
1.0
2.0
3.0
Date (YearWeek)

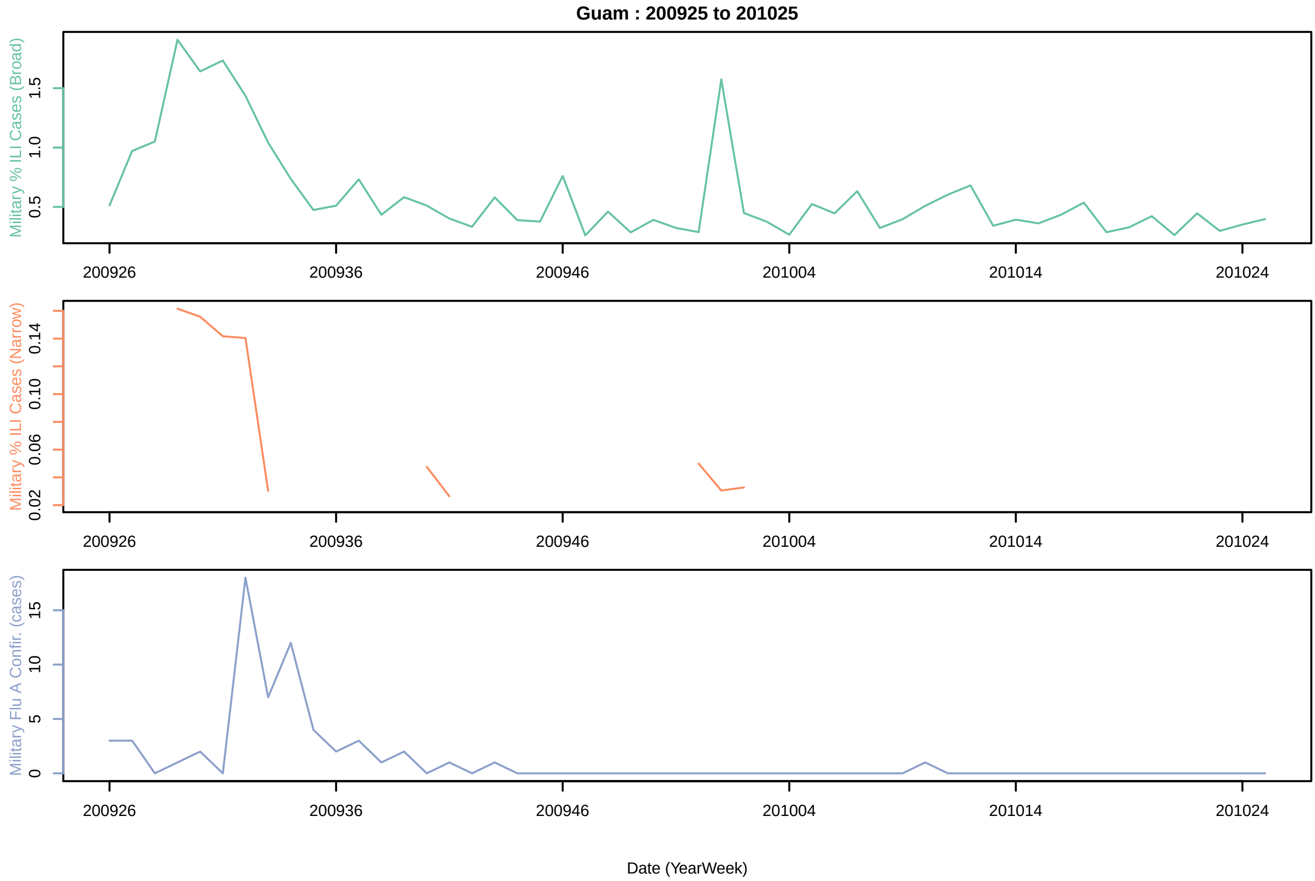
Guam : 200925 to 201025
Military % ILI Cases (Broad)
0.5
1.0
1.5
200926
200936
200946
201004
201014
201024
Military % ILI Cases (Narrow)
0.02
0.06
0.10
0.14
Military Flu A Confir. (cases)
0
5
10
15
Date (YearWeek)

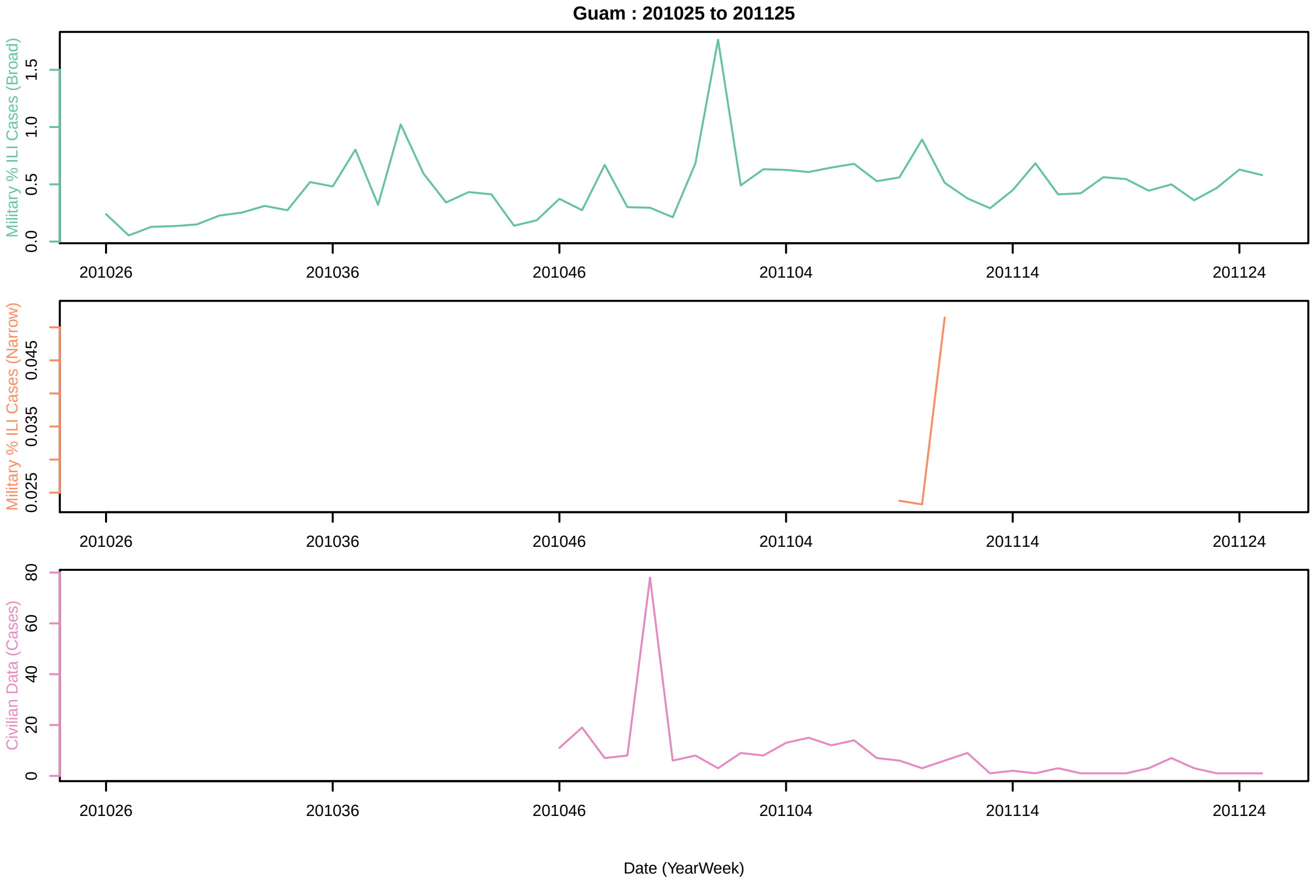

Guam : 201025 to 201125
Military % ILI Cases (Broad)
0.0
0.5
1.0
1.5
Military % ILI Cases (Narrow)
0.025
0.035
0.045
Civilian Data (Cases)
0
20
40
60
80
201026
201036
201046
201104
201114
201124
Date (YearWeek)

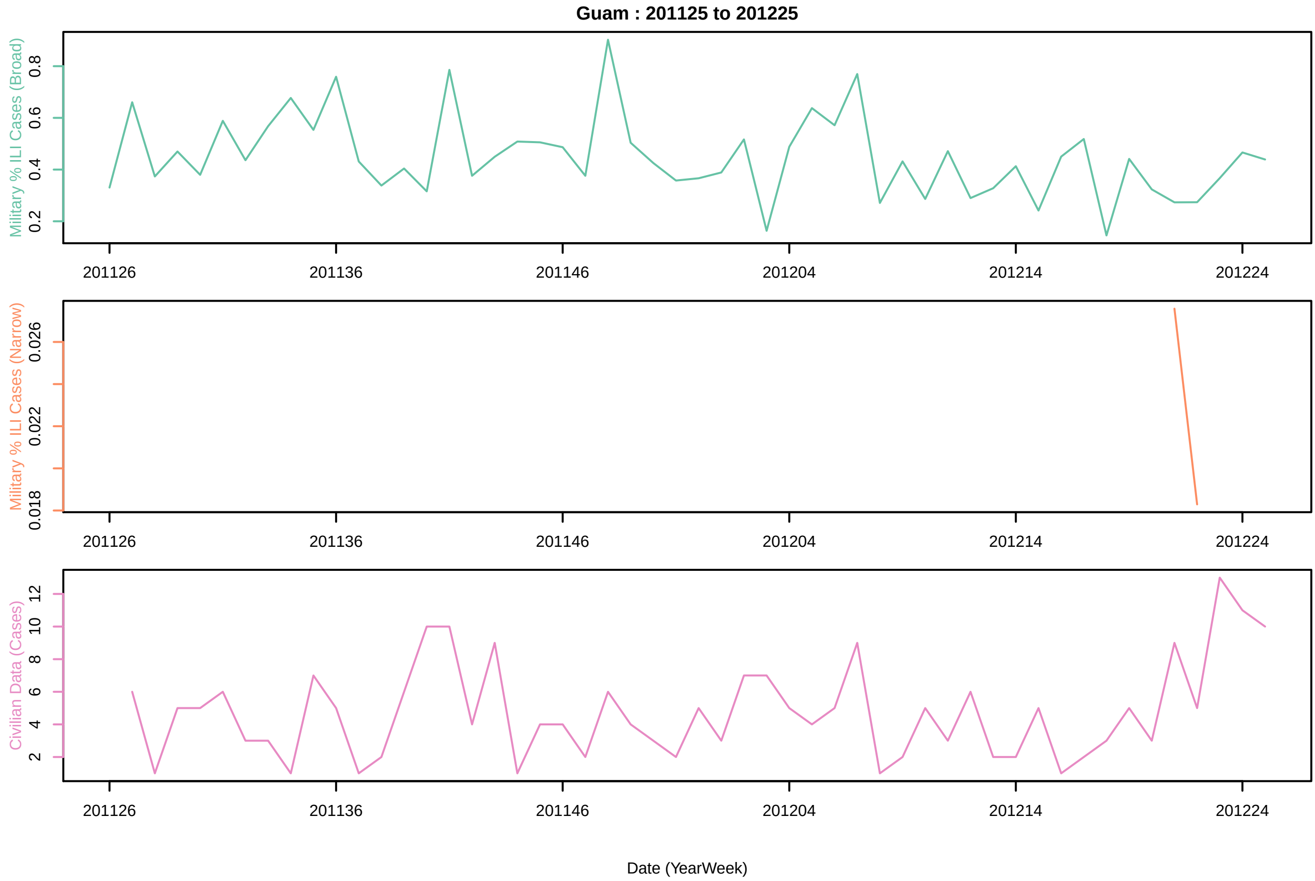

Guam : 201125 to 201225
Military % ILI Cases (Broad)
0.2
0.4
0.6
0.8
201126
201136
201146
201204
201214
201224
Military % ILI Cases (Narrow)
0.018
0.022
0.026
201126
201136
201146
201204
201214
201224
Civilian Data (Cases)
2
4
6
8
10
12
201126
201136
201146
201204
201214
201224
Date (YearWeek)

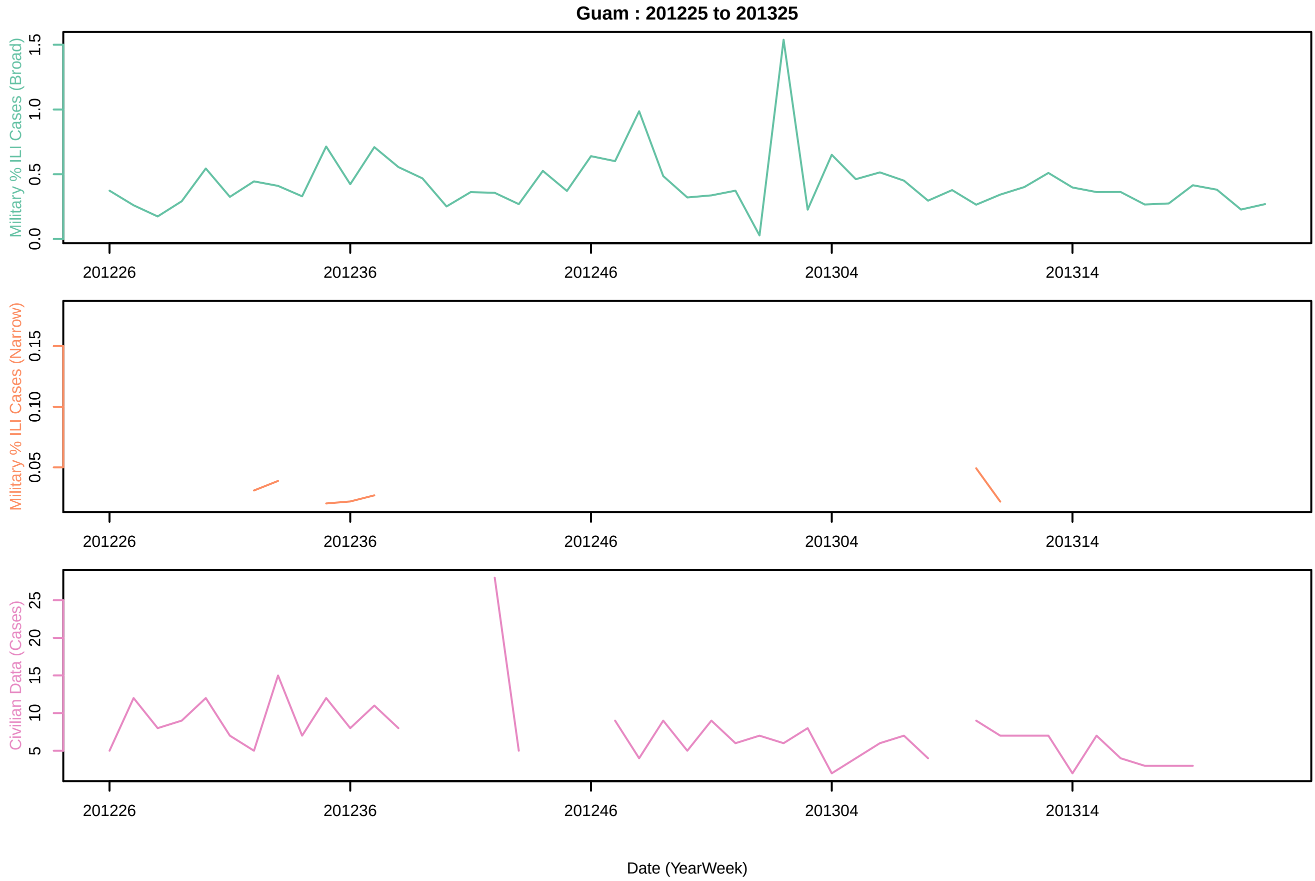
Guam : 201225 to 201325
Military % ILI Cases (Broad)
0.0
0.5
1.0
1.5
Military % ILI Cases (Narrow)
0.05
0.10
0.15
Civilian Data (Cases)
5
10
15
20
25
201226
201236
201246
201304
201314
Date (YearWeek)
